# A new giraffid (Mammalia, Ruminantia, Pecora) from the late Miocene of Spain, and the evolution of the sivathere-samothere lineage

**DOI:** 10.1371/journal.pone.0185378

**Published:** 2017-11-01

**Authors:** María Ríos, Israel M. Sánchez, Jorge Morales

**Affiliations:** 1 Departamento de Paleobiología, Museo Nacional de Ciencias Naturales-CSIC, Madrid, Spain; 2 Departmento de Faunas del Neógeno y Cuaternario, Institut Català de Paleontologia- Miquel Crusafont, Barcelona, Spain; Ecole normale superieure de Lyon, FRANCE

## Abstract

Giraffids include the only living giraffomorph ruminants and are diagnosed by the presence of bi-lobed canines and a special type of epiphyseal cranial appendages called ossicones. The family Giraffidae ranges from the latest early Miocene until today. However they are currently extant relics with only two living representatives, the African genera *Okapia* and *Giraffa*. Giraffids were much more diverse and widespread in the past, with more than 30 fossil species described. For the past decades a number of studies intended to resolve the phylogenetic relationships of the family, but due to the lack of really good cranial material no clear consensus was reached regarding the phylogenetic relationships amongst the different members of the group. The exceptionally complete remains of a new large giraffid from the late Miocene of Spain, *Decennatherium rex* sp. nov., allows us to improve and reassess giraffid systematics, offering a lot of new data, both anatomic and phylogenetic, on the large late Miocene giraffids of Eurasia. The results of our cladistic analysis show *Decennatherium* as a basal offshoot of a clade containing the gigantic samotheres and sivatheres, characterized by the presence of a *Sivatherium*-like ossicone-plan among other features. *Decennatherium* thus offers the most ancient evidence of this *Sivatherium*-plan and firmly establishes the early morphological patterns of evolution of a sivathere / samothere-clade that is defined as the less inclusive clade that contains *Decennatherium* and *Sivatherium*. Finally, this large group of four-ossiconed giraffids evolutionarily links Miocene Europe and Africa indicating vicariance / migration processes among the giraffid genetic pools separated by the Mediterranean Sea.

## Introduction

The Giraffidae is a clade of crown-pecorans that include the only extant survivors of the ancient Giraffomorpha [1]. Giraffids are diagnosed by the presence of a bilobed canine and a special type of epiphyseal and permanent cranial appendage called ossicone [1–3]. Ossicones are also present in palaeomerycids, thus they are found in at least two families within Giraffomorpha, the Palaeomerycoidea and the Giraffoidea [1]. Sánchez et al [4] hypothesized ossicone-like appendages as a possible basal trait for Giraffomorpha, however this has to be still fully corroborated by both the fossil record and a deep analysis of the anatomy and structure of the diverse giraffomorph cranial appendages.

Living giraffids are relics and represented by only two genera, the African *Okapia* [5] (monotypic) and *Giraffa* [6](could be four species) [7, 8], but it was very diverse during the Miocene, when it was widespread throughout the Old World. The earliest remains of giraffids come from the early and middle Miocene. These are the basal forms *Canthumeryx sirtensis* [9] (= *Zarafa zelteni*) from the lower Miocene Gebel Zelten (Libya), Wadi Moghara (Egypt), Kalodirr, Loperot, Buluk, Moruorot and Rusinga Island (Kenya), and Yeroham (Israel) [2, 9, 10] as well as from the middle Miocene of Maboko Island (Kenya) and Al-Sarrar (Saudi Arabia) [2]; and the form *Georgiomeryx georgalasi* [11] from Thymiana (Greece) [12]. Giraffid remains from the middle Miocene are scarce, but giraffid remains become very abundant from late Miocene deposits onwards, showing a high diversity of shapes and forms, found all over Eurasia and Africa [1, 13–15]. Thus, the late Miocene was a period of rapid diversification within the Giraffidae [16].

Two late Miocene giraffids from the Iberian Peninsula are currently described, the Vallesian *Decennatherium pachecoi* (MN9-10) and the early Turolian *Birgerbohlinia schaubi* (MN11) [15, 17, 18]. The purported close relationship of these two genera was recently rejected through a phylogenetic analysis [15]. However, some nodes of the tree are poorly supported and major politomies remain unresolved [15]. This is probably due to the plesiomorphic dentition and post-cranial skeleton of the Giraffidae, as well as its high morphological stability [10, 19], with the ossicones and metapodials as the most significant characters to analyze the variability within the Giraffidae.

**Fig 1:**
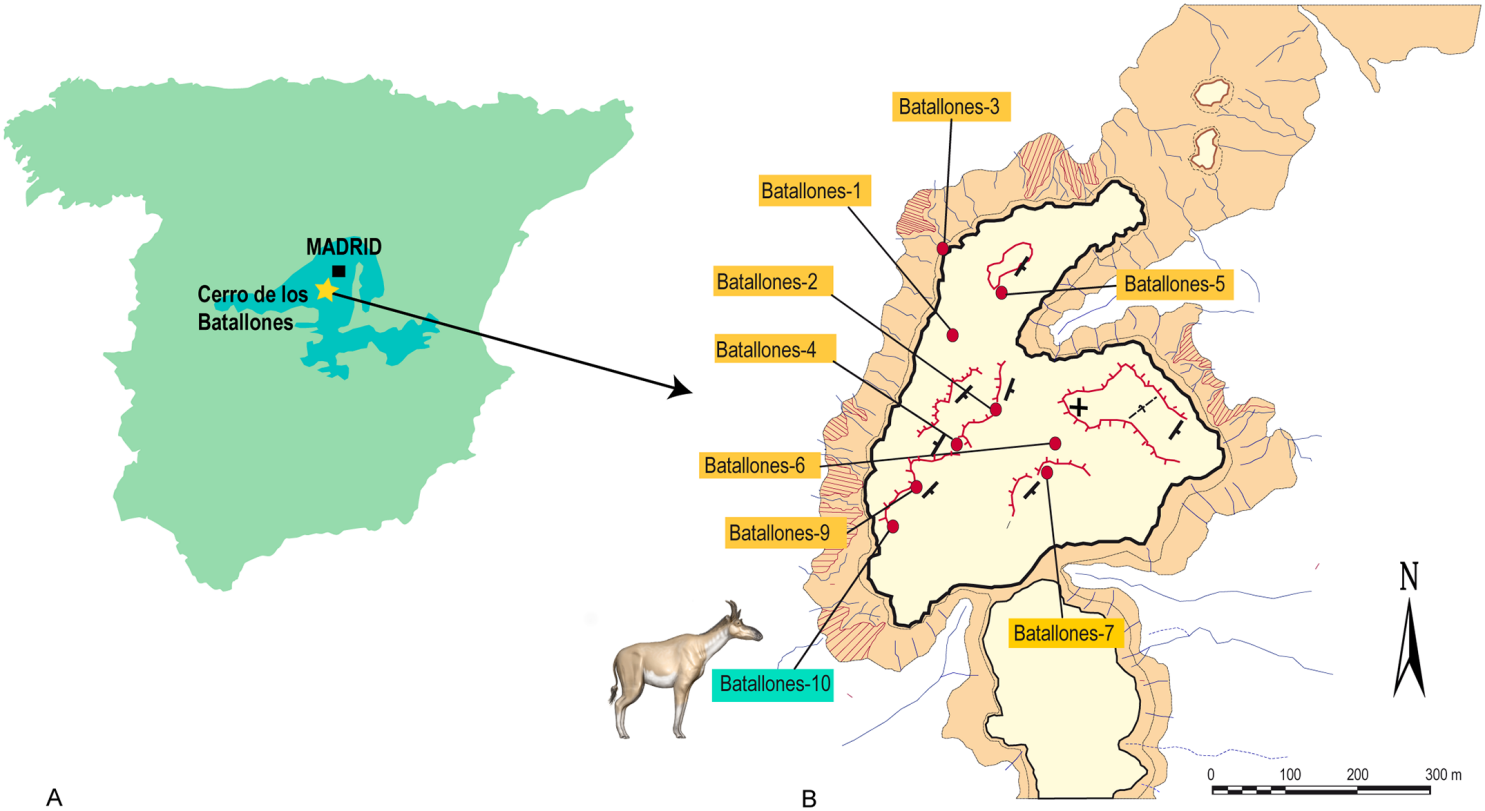
Location of Cerro de los Batallones. A, Location of Cerro de los Batallones (yellow star) within Spain (Modified from [20]; B, map of Cerro de los Batallones and location of the fossil sites (Modified from [20]).

The late Miocene (Vallesian; MN10) site of Batallones-10 (Madrid Basin, Madrid province, Spain; Fig 1) yielded abundant fossils of several individuals of a large giraffid that include beautifully preserved cranial, dental and postcranial remains of several individuals representing almost all skeletal elements. The sample includes several well-preserved skulls, making Batallones-10 one of the best collections of Miocene giraffids worldwide. This material provides important new data for testing previous hypotheses about the phylogenetic relationships of *Decennatherium* to other large Miocene Eurasian giraffids [15]. In this work, we describe the new giraffid from Batallones-10 and test its phylogenetic position within the Giraffidae, its relationships with the other two forms found in Spain, and also on the relationship hypothesis between *Decennatherium* and the subfamily Sivatherinae.

## Locality and geological setting

**Fig 2:**
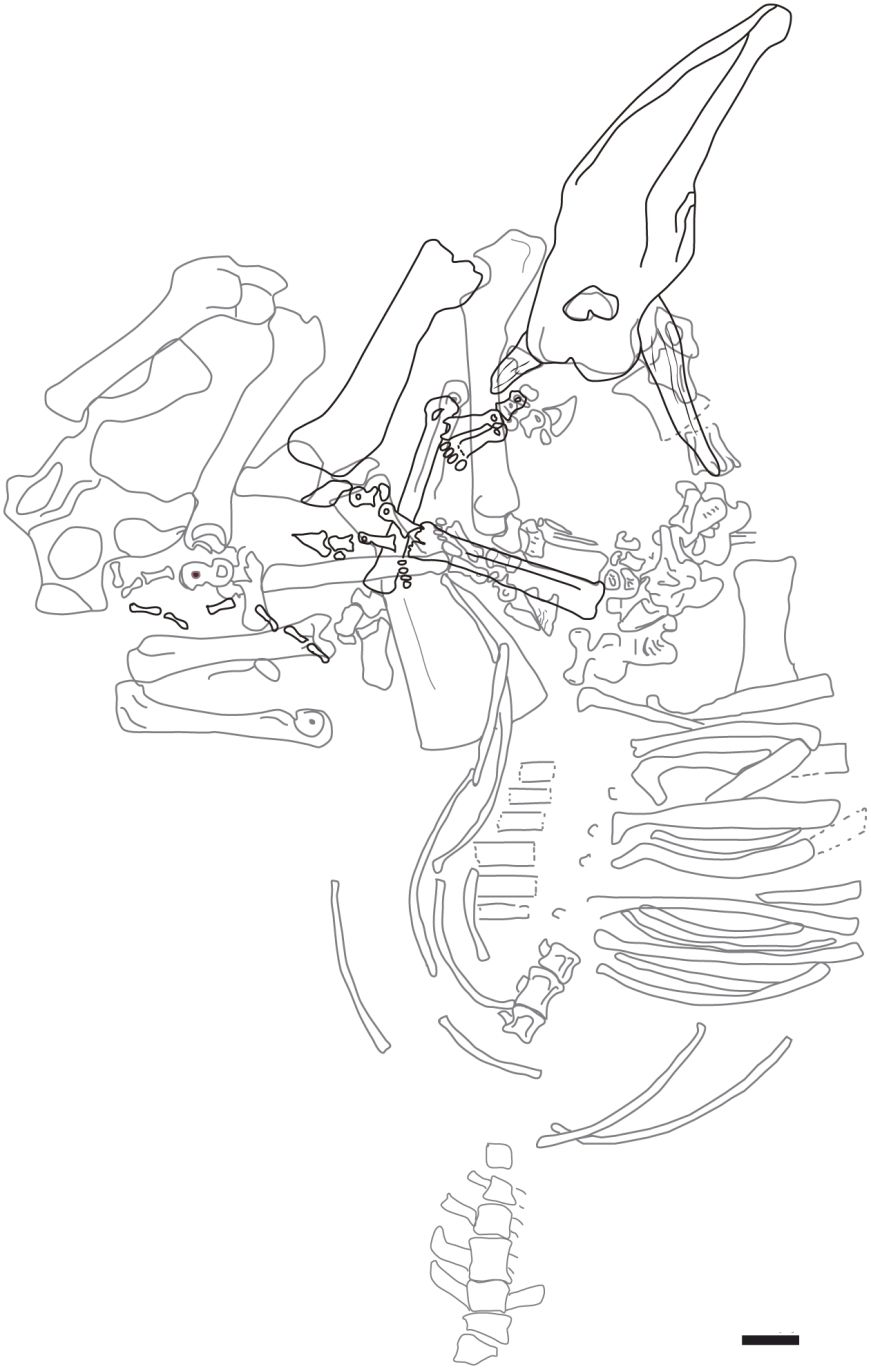
Articulated giraffid specimen from BAT10. Postcranial skeleton articulated with BAT10’13.E2-69 skull. Black, recovered during the 2013 campaign; grey, reverend during the 2014–16 campaigns. Scale bar equals 10 cm.

The fossil site-complex of Cerro de los Batallones (Batallones butte) is located near the town of Torrejón de Velasco (Madrid province), between the Jarama River Valley and the Prados-Guatén Depression in the central area of the Cenozoic Madrid Basin [20–24]. The butte towers in a zone of olive groves that extends near the town of Torrejón de Velasco (Madrid province, Spain). The fossil sites were found during mining operations at the butte (1991–2008) for the extraction of sepiolite. The fossil site-complex of Batallones comprises a system of nine, non-interconnected distinct sites located on the structural butte (Fig 1). The butte constitutes of a near horizontal sedimentary sequence of three units, all of which were deposited in terrestrial environments during the early Vallesian (early late Miocene, ca. 9 Ma). Fossil remains were accumulated in cavities formed in clay by a process of subsurface hydraulic erosion known as piping [20–24]. We differentiate two types of fossil accumulations in some Batallones localities, according to their internal stratigraphy and taxonomic composition. Lower level assemblages overwhelmingly dominated by carnivoran remains, while upper level assemblages mainly consist of fossils of mammalian herbivores [20–24]. Batallones-10 (BAT10 hereafter), discovered in 2007, was the last fossil locality discovered in Cerro de los Batallones (Fig 1). Though all sites are Vallesian in age, there are slight differences in composition of micro- and macromammals among the different fossil deposits that were attributed to minor temporal differences, indicating that BAT10 is older than BAT1, BAT3 and BAT5 [25]. The fossil assemblage of BAT10 is extraordinarily rich and totally different from the lower levels of BAT1 and BAT3 (the carnivore-traps). Apart from very large terrestrial tortoises, it is composed principally of mammalian macro-herbivores, mainly giraffids and small-sized hipparionine equids (from colts to fully grown individuals), and also proboscideans, rhinocerotids, moschids, medium-sized bovids and suids, as well as various micromammals, squamates and birds. Carnivoran remains are scarce [21, 22, 26] while giraffid fossils are very abundant, including the exceptional finding in 2013 of a complete articulated giraffe individual (Fig 2).

## Materials and methods

### Material

The description is based on the complete fossil sample from BAT10 publicly curated by the Paleobiology Department at the Museo Nacional de Ciencias Naturales-CSIC (Madrid, Spain). We list the extensive specimen numbers in S1 and S2 Tables. The comparative material of *Decennatherium pachecoi* [17] comprises the entire sample of fossils from Los Valles de Fuentidueña (MNCN-CSIC, Madrid, Spain). Other *Decennatherium pachecoi* data includes the bibliography on the material of Nombrevilla, Relea, Pedrajas de San Esteban, Saldaña [17], Campisalabos[27], La Roma and Masia del Barbo [15, 17, 28, 29].

Comparative crania and postcrania material of *Birgerbohlinia schaubi* [17] come from Piera (Torrent dels Traginers, Piera, MN11) and from Crevillente-2 (Crevillente, Alicante, MN11), and was curated by the Institut Català de Paleontologia (Sabadell, Spain) and the Museo de Geologia de la Universidad de Valencia (Burjasot, Spain).

We collected data regarding the middle Miocene basal giraffid *Injanatherium arabicum* [30] from Al Jadidah (Saudi Arabia) from the casts at the MNCN-CSIC (Madrid, Spain).

Other cranial and postcranial data were collected from material curated by the AMNH (New York, USA), the NHM (London, UK) and MNHN (Paris, France). This includes the early Miocene *Canthumeryx sirtensis* [9] from Gebel Zelten (Libya). It also includes the middle Miocene *Giraffokeryx punjabiensis* [31], from Bhandar, Chinji, Hasnot, Kashmir, Nathot, Phadial and Ramnagar (Pakistan) and Chakrana (India), and *Giraffokeryx primaevus* from Fort Ternan (Kenya) [32]. From the same collections we also collected data of the Greek-Iranian late Miocene giraffids, including *Samotherium major* [33], and *Samotherium boissieri* [34] from the early Turolian to late Pliocene of Samos (Greece); as well as *Palaeotragus rouenii* [35] from Pikermi (Greece); *Bohlinia attica* [36] from Pikermi, Thessaloniki and Samos (Greece) and Maragha, (Iran); *Helladotherium duvernoyi* [35] from Pikermi, Thessaloniki, Samos (Greece), and Cucuron (France); *Alcicephalus neumayri* from Maragha (Iran) [37], and *Palaeotragus coelophrys* from Maragha (Iran). The data of the Chinese *Schansitherium tafeli* [38] and *Samotherium sinense* [39] from the late Miocene and early Pliocene of Shansi (China) also were collected at the same collections. Finally, we studied the late Miocene giraffids of India and Pakistan *Bramatherium perimense* [40] from Perim Island (Yemen) and *Bramatherium megacephalum* [41], from Dhok Pathan (Pakistan) as well as the early Pleistocene *Sivatherium giganteum* [42], from the upper Siwaliks near Kalka, Kharian, Nahun, Nàhan, Pinjaur, Siswan, and Chandigarh (India); and the Plio-pleistocene *Giraffa jumae* [43] from Çalta (Turkey) and Rawe (Kenya), also curated in the collections mentioned above. We collected additional anatomical data of osteological material of the extant giraffids *Giraffa camelopardalis*[6] and *Okapia johnstoni* [5] from the collections of comparative anatomy of the MNCN-CSIC (Madrid, Spain) and the AMNH (New York, USA).

The rest of the morphological and biometrical information in this paper was collected from several publications, including *Canthumeryx sirtensis* [9] (= *Zarafa zelteni*) from Gebel Zelten (Libya), Moruorot and Rusinga (Kenya)[9, 10]; *Georgiomeryx georgalasi* [11] from Thymiana (Greece) [12]; *Giraffokeryx primaevus [32]* (= *Palaeotragus primaevus*) from Fort Ternan (Kenya) [32], and *Injanatherium hazimi* [44] from Gebel Hamrim (Iraq) [44, 45]. We collected bibliographic data on other Miocene and Pliocene giraffids including: *Schansitherium tafeli* [38] from Shansi (China) [33, 39, 46]; *Alcicephalus neumayri* [37], from Maragha (Iran) [37, 47, 48]; *Samotherium major* [33] from Samos (Greece) and Kemiklitepe (Turkey) [33, 49, 50]; *Palaeotragus coelophrys* [37], from Maragha (Iran) and Ravin de la Pluie (Greece) [33, 47, 51, 52]; *Palaeotragus microdon* [33] from Gansu and Shansi (China) [33] and *Helladotherium duvernoyi* [35] from Pikermi, Samos and Nikiti (Greece), Vozarci and Karaslari (Macedonia), Hadjidimovo and Kalimantsi (Bulgaria), Maragha (Iran) and Cucuron (France) [31, 35, 50, 53–55]. We also collected bibliographic data of the larger giraffids *Sivatherium maurusium* [56], from Koobi Fora (Kenya) and Laetoli (Tanzania) [57, 58], *Sivatherium olduvaiense*[59] from Olduvai and Garussi Korongo (Tanzania) [60–62] and *Sivatherium hendeyi* [63] from Cape Province (South Africa). Among the more recent giraffids we include data of *Giraffa jumae* [43] from East Rudolf and Rawe (Kenya) and Olduvai (Tanzania) [64] and *Giraffa stillei* [61] (= *Okapia stillei*, *Giraffa gracilis*) from Laetoli (Tanzania)[61, 64–66].

### Measurements

We mostly follow the measurements proposed by Quiralte [67] but we created new ones for elements that needed new measurements (Figures A-J in S1 Text). All measurements are presented in the Supporting Information (S1, S2 and S3 Tables) in order to facilitate complete descriptions. We took all measurements with digital calipers.

### Nomenclature

For anatomical nomenclature of the cranial and postcranial skeleton we follow Barone [68], and for nomenclature of the dentition Azanza [69] and Sánchez and Morales[70]. We use the vertebral nomenclature of Danowitz et al. [68, 71–73].

### Institutional / Technical abbreviations

AMNH, AM, American Museum of Natural History, New York, USA; ICP, Institut Català de Paleontologia- Miquel Crusafont, Barcelona, Spain; MGUV, Museu de Geologia de la Universitat de València; MNCN-CSIC, Museo Nacional de Ciencias Naturales-CSIC, Madrid, Spain; MNHN, PIK, Musèum national d’Histoire naturelle, Paris, France; NHM, BM, M, Natural History Museum, London, UK; SAM, PQ, South Arican Museums.

### Phylogenetic analysis

Reconstructing the phylogenetic relationships within the Giraffidae has been historically difficult due to the lack of really complete cranial material and the high morphological stability of most of dental and postcranial elements, meaning that there is little change amomg taxa over time. Rios et al. [15] performed a phylogenetic analysis of the Giraffidae that showed the first cladistic evidence of the relationship between *Decennatherium* and samotheres. The exceptional remains from BAT10 allow us to present here a more complete phylogenetic analysis with more morphological characters and the best-known giraffid species, with the inclusion of *Decennatherium rex* sp. nov. adding very useful information to the morphological dataset. We chose the living cervid *Capreolus capreolus* [6] as the outgroup. The ingroup is composed of the Miocene palaeomerycids *Xenokeryx amidalae* [4] and *Ampelomeryx ginsburgi* [74], plus the giraffids *Afrikanokeryx leakeyi*, *Canthumeryx sirtensis*, *Georgiomeryx georgalasi*, *Injanatherium hazimi*, *Injanatherium arabicum*, *Giraffokeryx punjabiensis*, *Giraffokeryx primaevus*, *Bohlinia attica*, *Giraffa stillei*, *Giraffa jumae*, *Giraffa camelopardalis*, *Palaeotragus rouenii*, *Palaeotragus microdon*, *Palaeotragus coelophrys*, *Okapia johnstoni*, *Samotherium sinense*, *Schansitherium tafeli*, *Deccenatherium pachecoi*, *Decennatherium rex* sp. nov. *Samotherium major*, *Samotherium boissieri*, *Alcicephalus neumayri*, *Birgerbohlinia schaubi*, *Sivatherium hendeyi*, *Helladotherium duvernoyi*, *Sivatherium maurusium/olduvaiense*, *Sivatherium giganteum*, *Bramatherium perimense* and *Bramatherium megacephalum*. We compiled and transformed the data matrices using Mesquite 3.04 (Windows version).

#### Maximum Parsimony analysis

The data matrices and the lists of characters are presented as Supporting Information (S2 Text, S1 File, S4 Table). We perform a Maximum Parsimony analysis to check the position of the giraffid from BAT10 within the Giraffidae, exploring provisionally the phylogenetic structure of the group. We use a morphological dataset of 111 characters (cranial, dental and postcranial), with 31 taxa including those in the outgroup. We used TNT v1.5-beta software [75] to analyze the dataset. All characters are non-additive and un-weighted, and we analyzed the trees using a Traditional Search method (heuristic algorithm) with Tree Bisection Reconnection (TBR) and 1000 replicates (holding 10 most parsimonious trees for each replicate). We used Bremer (BREMER.RUN) to assess branch support.

### Nomenclatural acts

The electronic edition of this article conforms to the requirements of the amended International Code of Zoological Nomenclature, and hence the new names contained herein are available under that Code from the electronic edition of this article. This published work and the nomenclatural acts it contains have been registered in ZooBank, the online registration system for the ICZN. The ZooBank LSIDs (Life Science Identifiers) can be resolved and the associated information viewed through any standard web browser by appending the LSID to the prefix “http://zoobank.org/”. The LSID for this publication is: urn:lsid:zoobank.org:pub:68375E3F-6C6F-45F9-9F89-8B10D3608C6A. The electronic edition of this work was published in a journal with an ISSN, and has been archived and is available from the following digital repositories: PubMed Central, LOCKSS.

## Systematic paleontology

MAMMALIA Linnaeus, 1758 [6]

CETARTIODACTYLA Montgelard, Catzeflis and Douzery, 1997 [76]

RUMINANTIA Scopoli, 1777 [77]

PECORA sensu Webb and Taylor, 1980 [78]

GIRAFFIDAE Gray, 1821[79]

*Decennatherium* Crusafont, 1952 [17]

### Type species

*Decennatherium pachecoi* [19]

Genus *Decennatherium* Crusafont, 1952 [17]

#### Emended diagnosis

Large giraffid with two pairs of ossicones. Smaller anterior pair located above the orbits and anteriorly oriented. Larger posterior pair located posterior to the orbits and posteriorly oriented. Posterior ossicones curved, showing a high number of ridges on their surface. Elongated dp3 and dp4. The dp3 has a posterolingual conid lingually attached to the posterior stylid, mesolingual conid absent, and anterior conid and anterior stylid separated. Elongated DP3 with the anterior lobe almost twice as long as the posterior one, presence of a strong and very wide anterior style, strong buccal cone, and unfolded lingual cone. p2 very simple, without mesolingual conid and with a coniform mesobuccal conid. p3 with posterobuccal conid always attached to the mesobuccal conid, and absence of a mesolingual conid. p4 of variable morphology, with continuous lingual wall, and posterobuccal conid always attached to the mesobuccal conid. Lower molars sporting weak metastylid that fades with wear, and no buccal or lingual cingulids. The m3 has a well-developed third lobe, a semicircular hypoconulid and a strong entoconulid. Upper molars are more longer than wide, with parallel and weak styles, poorly developed buccal cingulum, separated lingual cone and metaconule, occlusally exposed dentine area that fuses late with wear, and no basal pillars. Postcranial skeleton with limbs of medium length and robustness. Metacarpal III–IV with a shallow to medium posterior trough.

### *Decennatherium rex* sp. nov.

(Figs 2–41)

**Fig 3:**
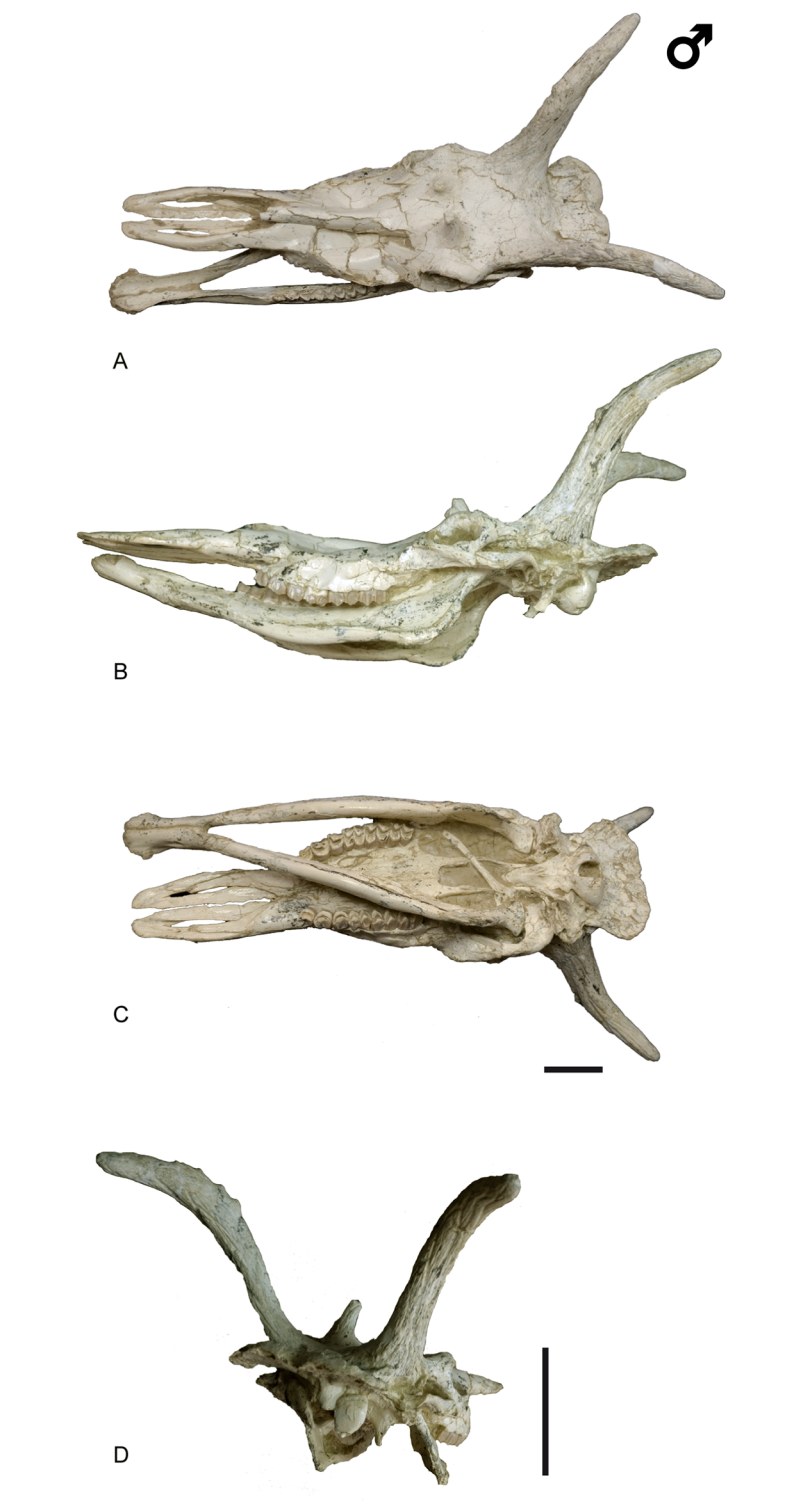
Cranial remains of *Decennatherium rex* sp. nov. from BAT10. A-C, BAT10’13.E2-69, skull in A, dorsal view; B, medial/lateral view; C, ventral view; D, caudal view. Scale bar equals 10 cm.

**Fig 4:**
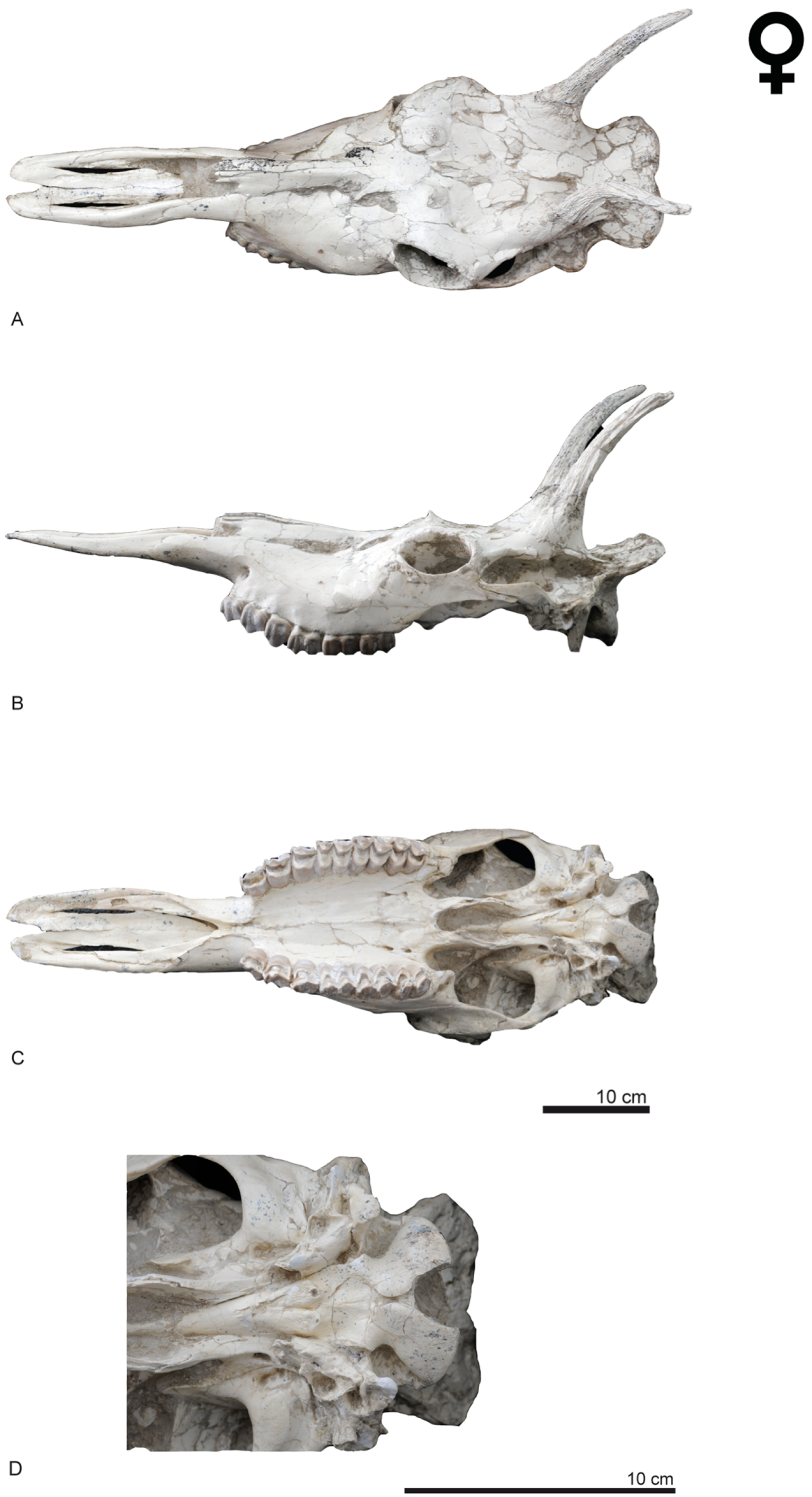
Cranial remains of *Decennatherium rex* sp. nov. from BAT10. A-C, BAT10’08.G3-91, skull in A, dorsal view; B, medial/lateral view; C, ventral view; D, ventral view of the basioccipital region. Scale bar equals 10 cm.

**Fig 5:**
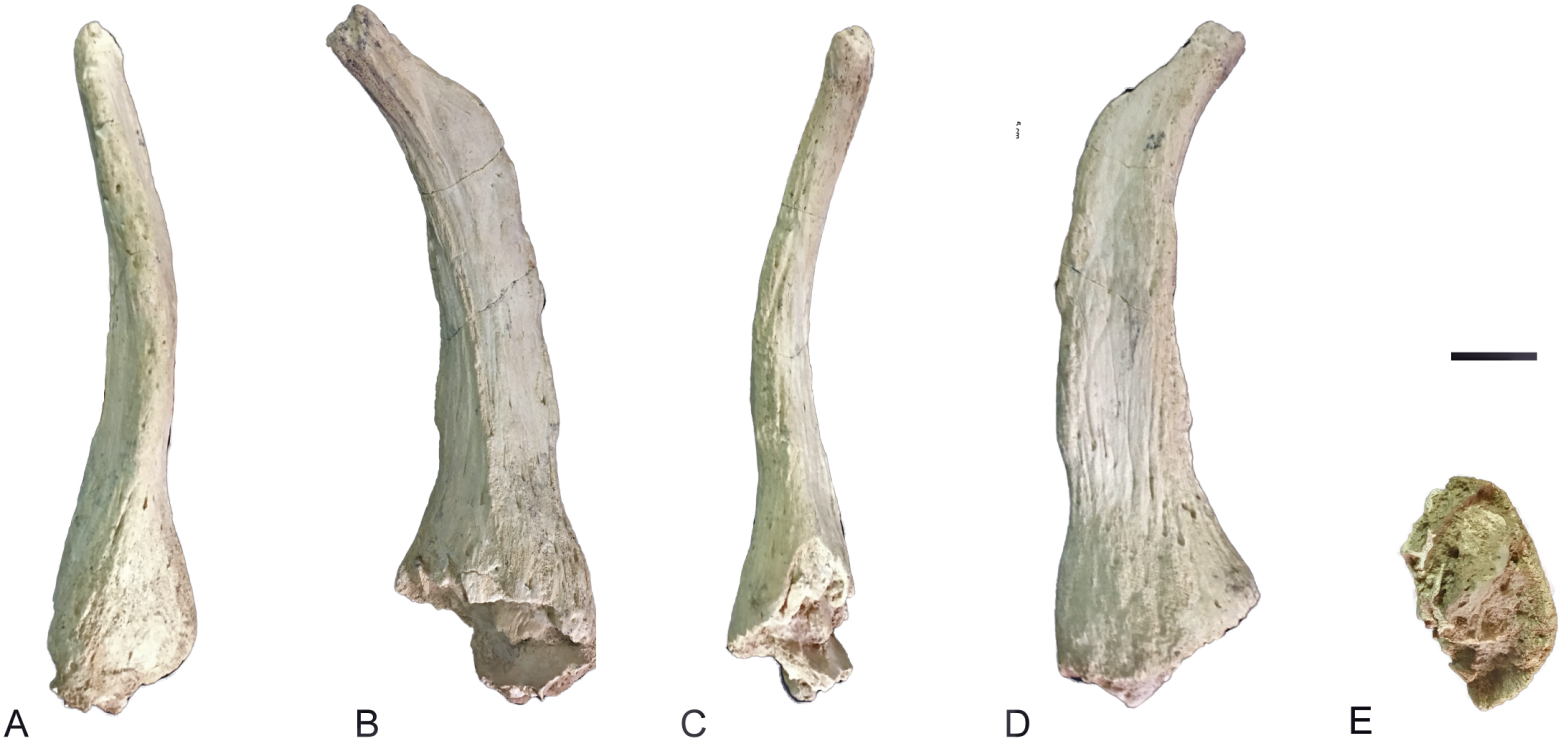
Posterior ossicone of *Decennatherium rex* sp. nov. from BAT10. A-E, BAT10’16.D4-20, right posterior ossicone in A, cranial view; B, lateral view; C, caudal view; D, medial view; E, proximal view. Scale bar equals 5 cm.

**Fig 6:**
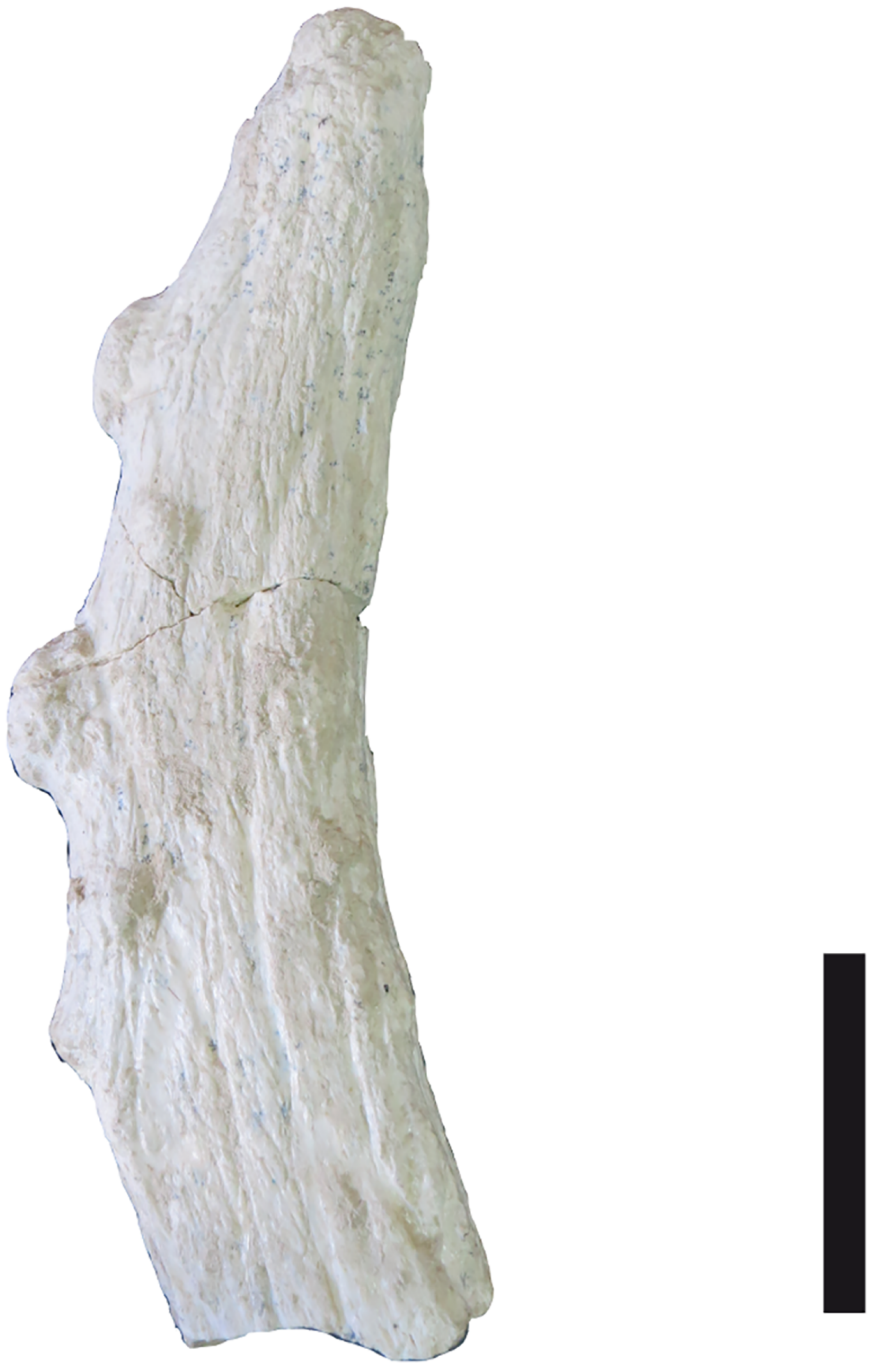
Fragment of a posterior ossicone with bumps of *Decennatherium rex* sp. nov. from BAT10. BAT10’07.G3-9. Scale bar equals 5 cm.

**Fig 7:**
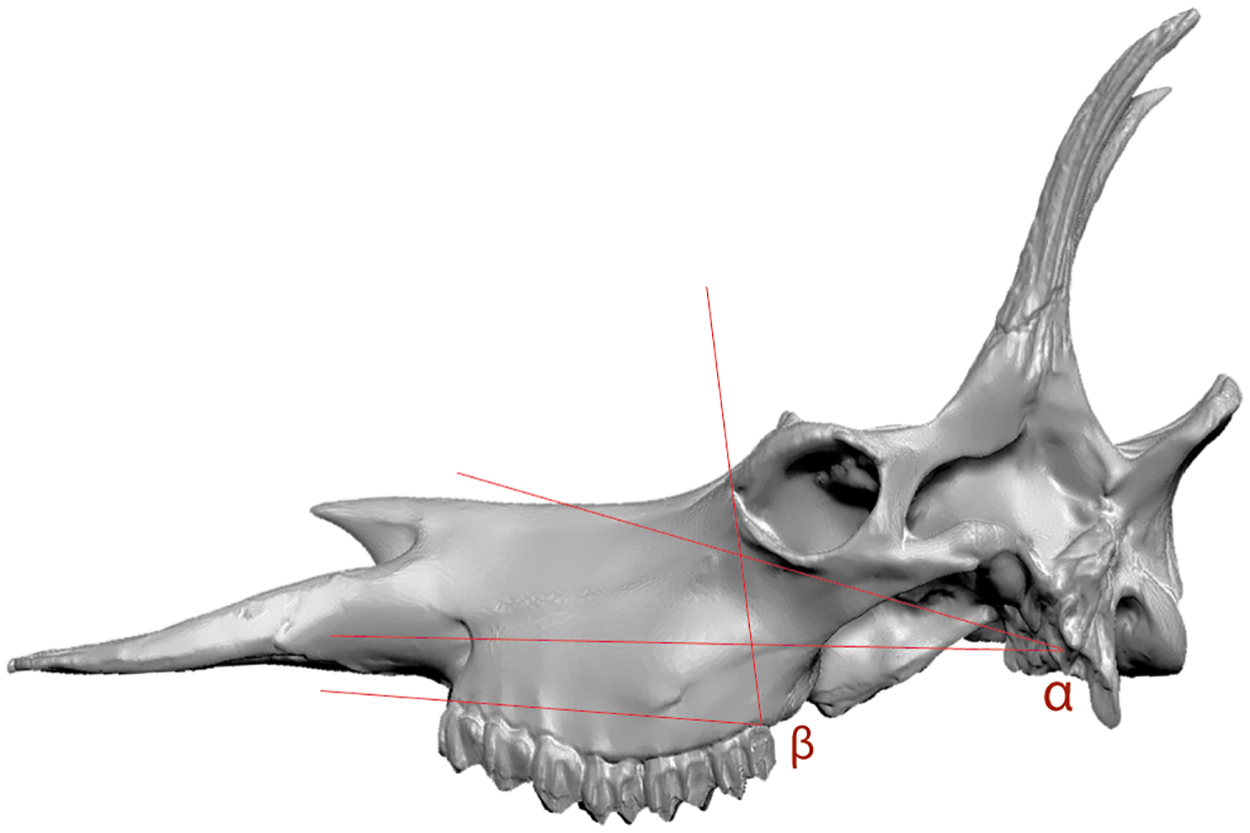
Skull angles (characters 52–53) of *Decennatherium rex* sp. nov. from BAT10. Defined by (8). Scale bar equals 10 cm. 3D correction by Oscar Sanisidro using ZBrush software.

**Fig 8:**
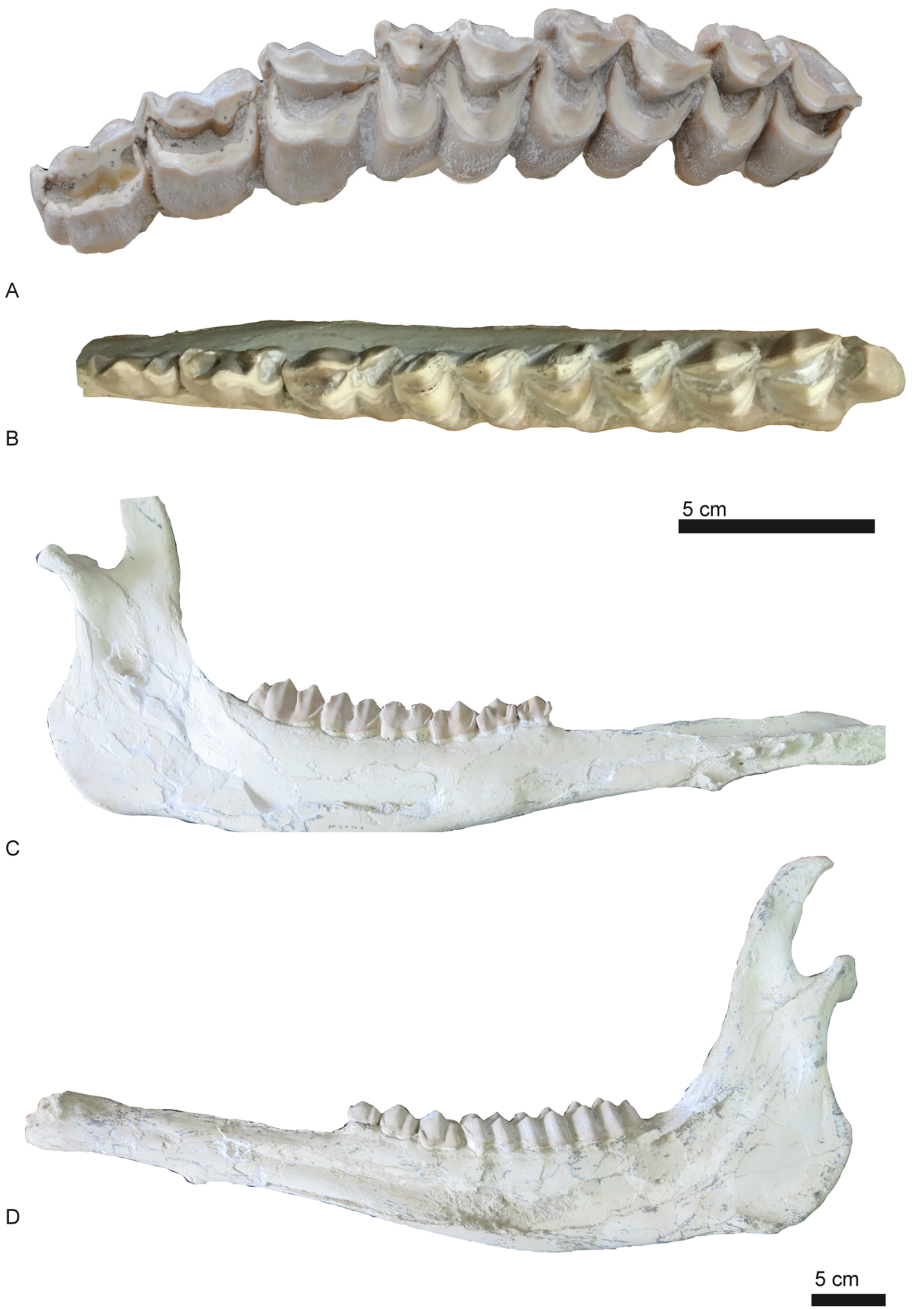
Dental remains of *Decennatherium rex* sp. nov. from BAT10. A, BAT10’08.G3-91, left P2-M3 in occlusal view; B-D, BAT10’10.F2-18, left p2-m3 in B, occlusal view; C, lingual view; D, buccal view. Scale bar equals 5 cm.

**Fig 9:**
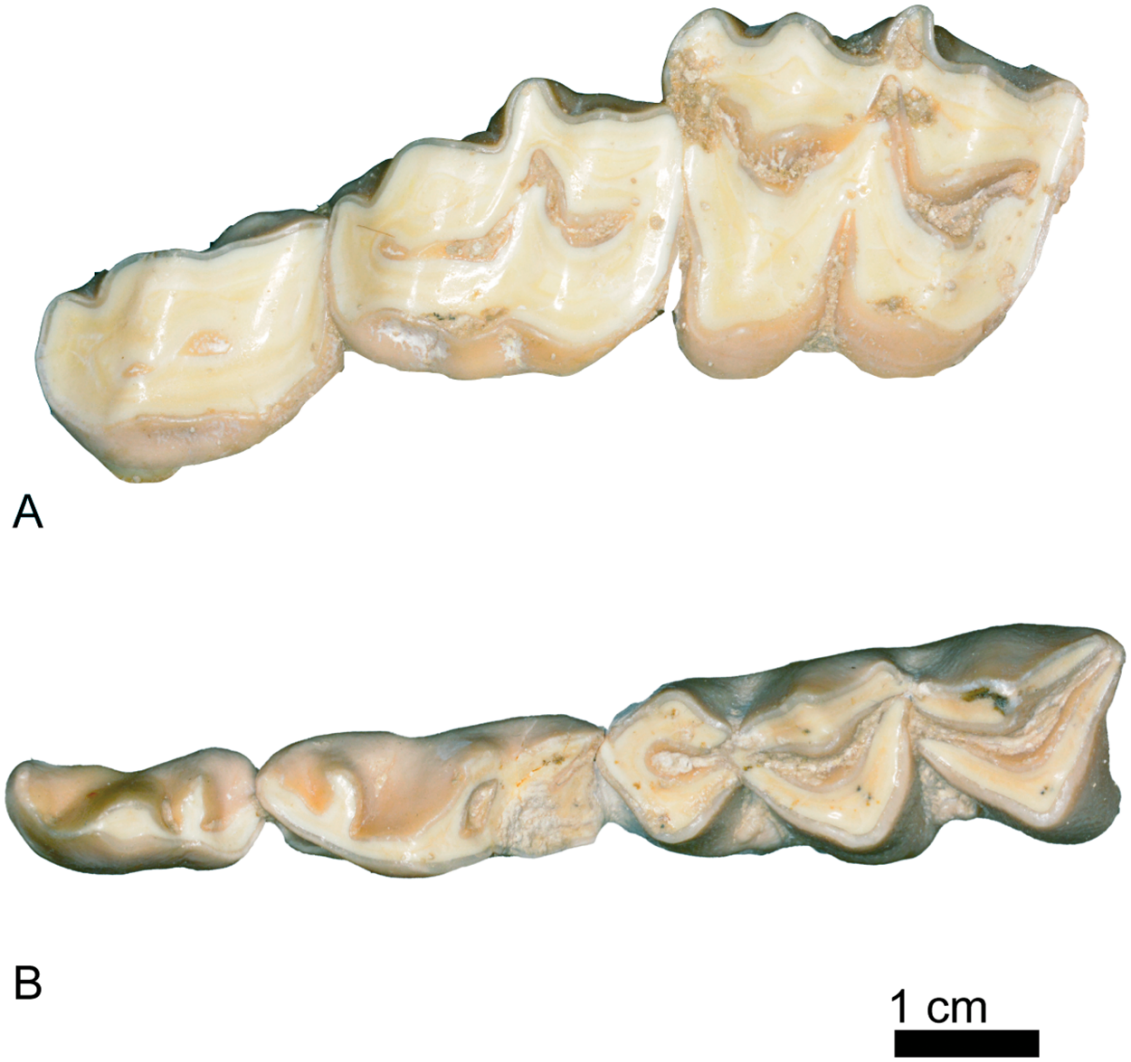
Deciduous teeth rows of *Decennatherium rex* sp. nov. from BAT10. A, BAT10’09.G2-177, left DP2-DP4 in occlusal view; B, BAT10’09.F4-22, left dp2-dp4 in occlusal view. Scale bar equals 1 cm.

**Fig 10:**
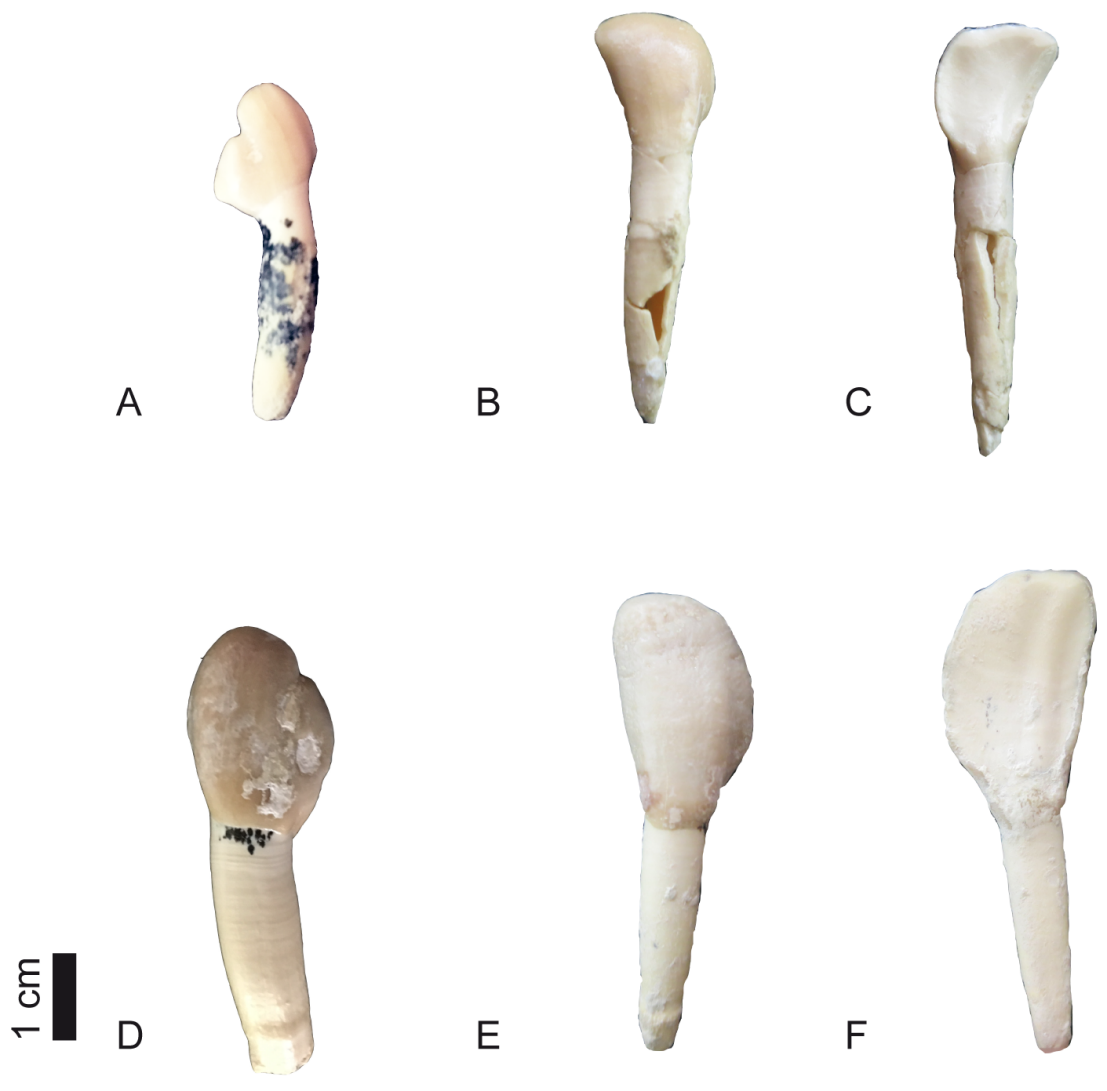
Dental remains of *Decennatherium rex* sp. nov. from BAT10. A, BAT10’07.G3-21, deciduous canine in buccal view; B-C, BAT10’12.D6-12, deciduous incisor in B, buccal view; C, lingual view; BAT10’15.D4-63, canine in buccal view; D, BAT10’07.G2-12, buccal view. Scale bar equals 1 cm.

**Fig 11:**
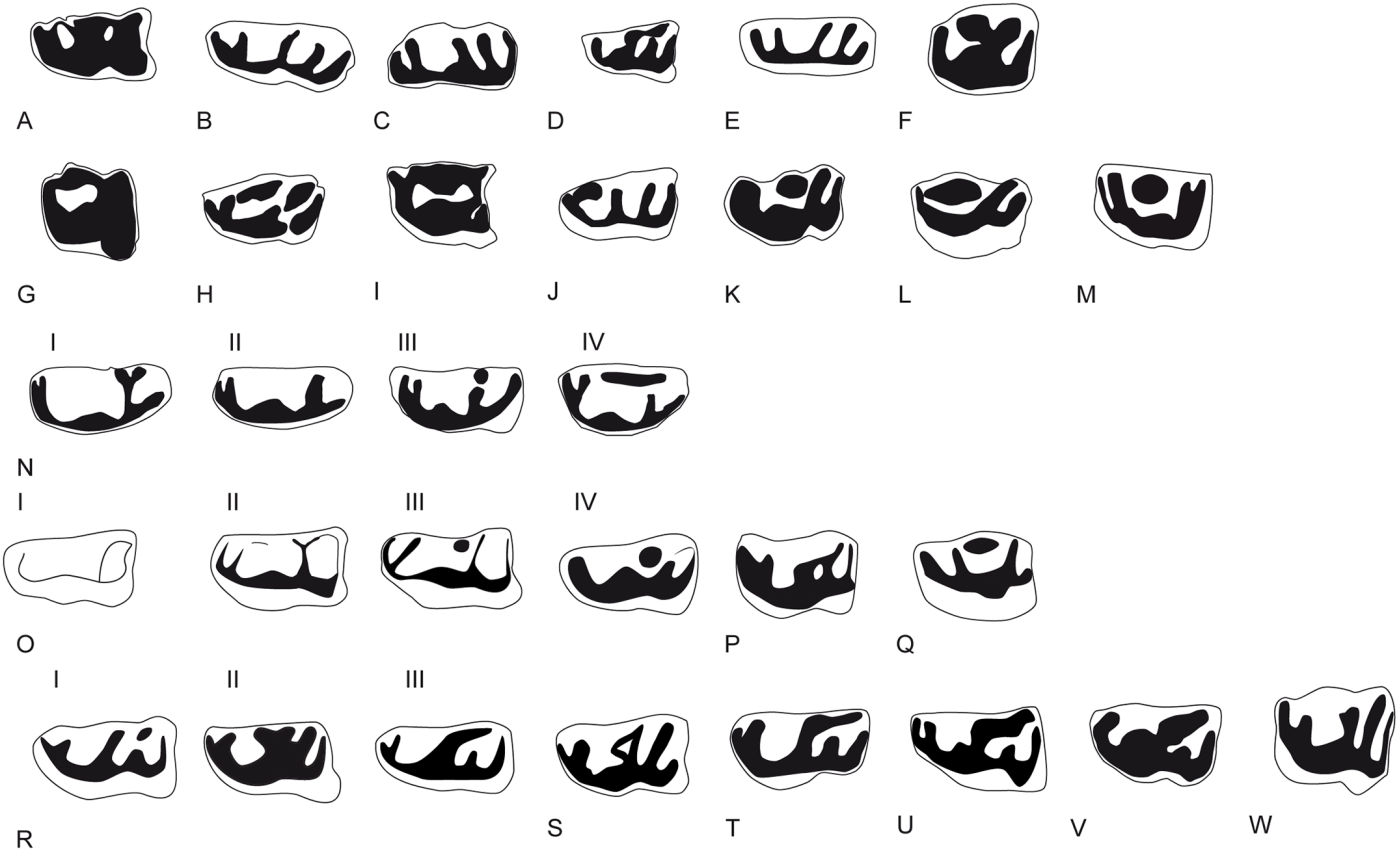
Occlusal views of the p3 of the giraffid species included in this work. A, M26675-P80, *C*. *sirtensis*; B, from de Bonis et al., 1997, Teftfig. 3c, *G*. *georgalasi*; C, AM-127634, *I*. *arabicum*; D, AM-29946, *G*. *punjabiensis*; E, M30159, *G*. *primaevus*; F, MNCN-3439, *G*. *camelopardalis*; G, M21466, *G*. *jumae*; H, M8367, *P*. *rouenii*; I, AM-26360, *P*. *microdon*; J, MAR-669, *P*. *coelophrys*; K, MNCN-5226, *O*. *johnstoni*; L, from Schlosser, 1903 Tafel 9.10; *S*. *sinense*; M, from Hamilton, 1978, Fig 5a, *S*. *tafeli*; N, *D*. *rex*; I, BAT10’10.F2-18; II, BAT10’11.E2-21; III, BAT10’12.D6-85; IV; BAT10’11.C5-6; O, *D*. *pachecoi*, I, MNCN-43462; II, MNCN-43463; III, MNCN-43461; IV; MNCN-43465; P, from Hamilton, 1978, Fig 5, *S*. *boisieri*; Q, from Hamilton, 1978, Fig 5, *A*. *neumayri*; R, *B*. *schaubi*, I, CR2-215; II, CR2-879; III, VP-868; S, SAM-PQ-L31137 from Harris, 1976 Fig 4b, *S*. *hendeyi*; T, MNHN-1911-34, *H*. *duvernoyi*; U, KNM-ER-777 from Harris, 1991 Fig 4.22, *S*. *maurusium*; V, NHM-16163, *S*. *giganteum*; W, AM-19670, *B*. *megacephalum*. Images not to scale and modified so they have the same orientation.

**Fig 12:**
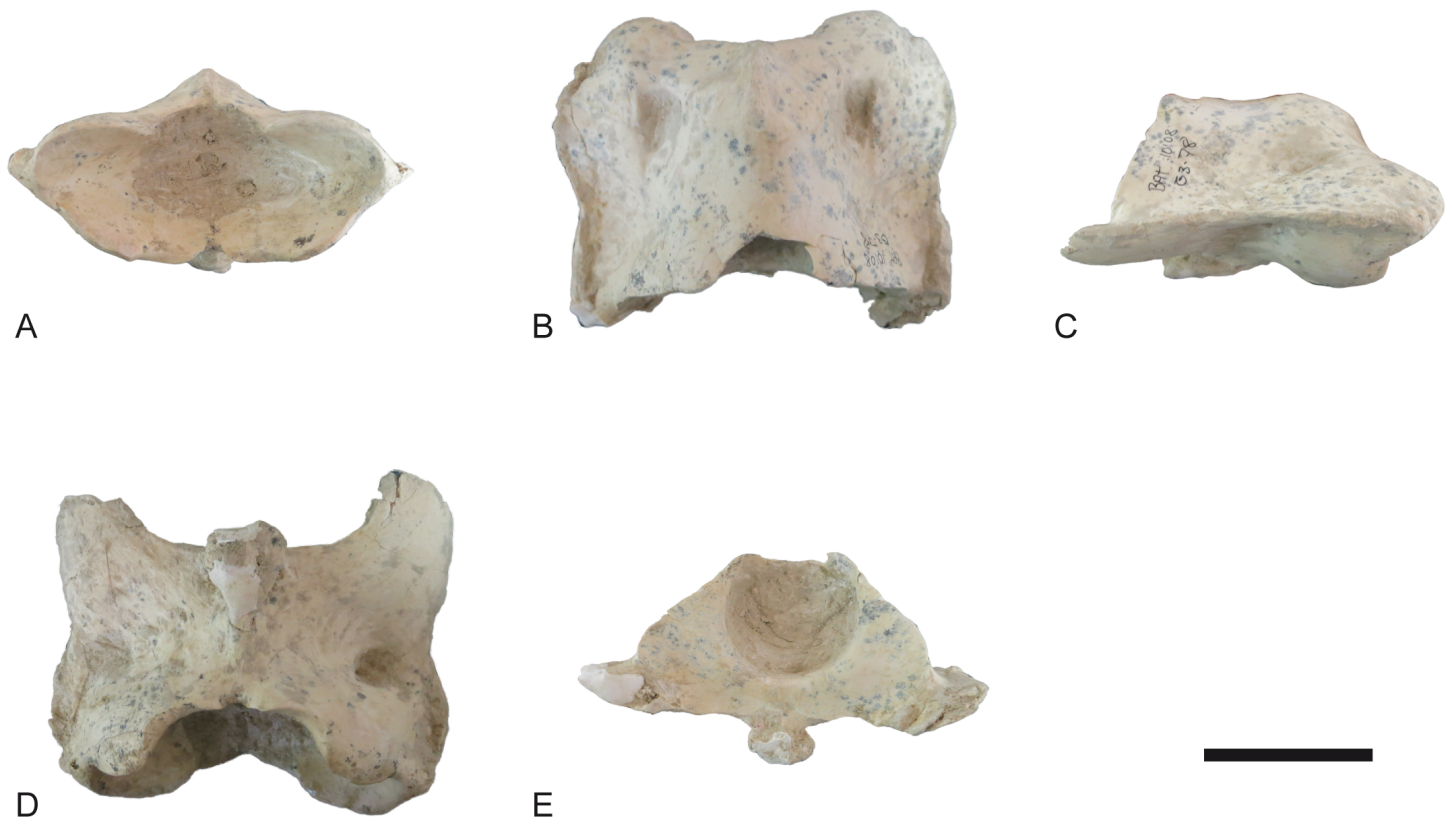
Atlas of *Decennatherium rex* sp. nov. from BAT10. A-E, BAT10’08.G3-79, in A, cranial view; B, dorsal view; C, medial/lateral view; D, ventral view; E, caudal view. Scale bar equals 5 cm.

**Fig 13:**
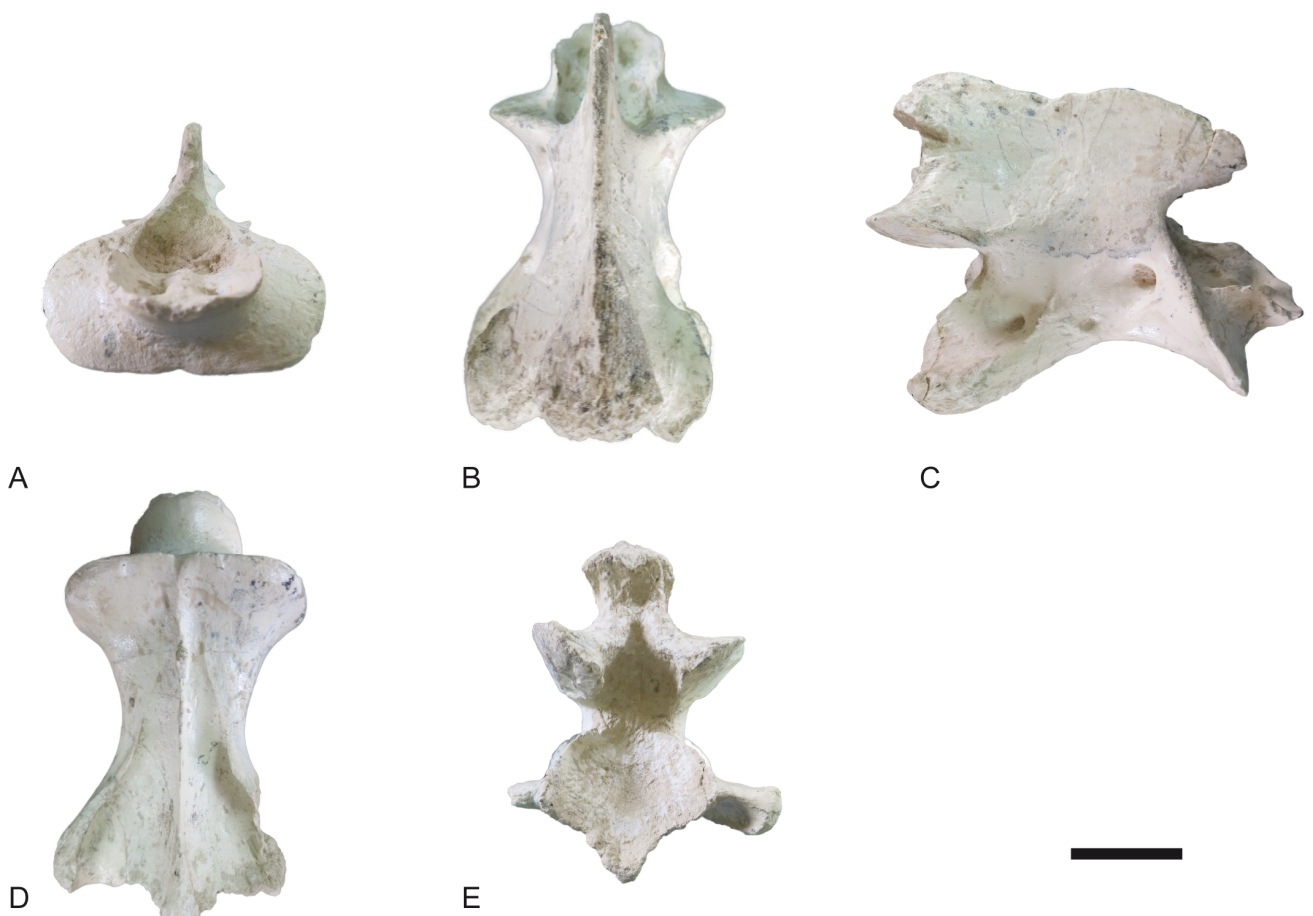
Axis of *Decennatherium rex* sp. nov. from BAT10. A-E, BAT10’14.D4-32, in A, cranial view; B, dorsal view; C, medial/lateral view; D, ventral view; E, caudal view. Scale bar equals 5 cm.

**Fig 14:**
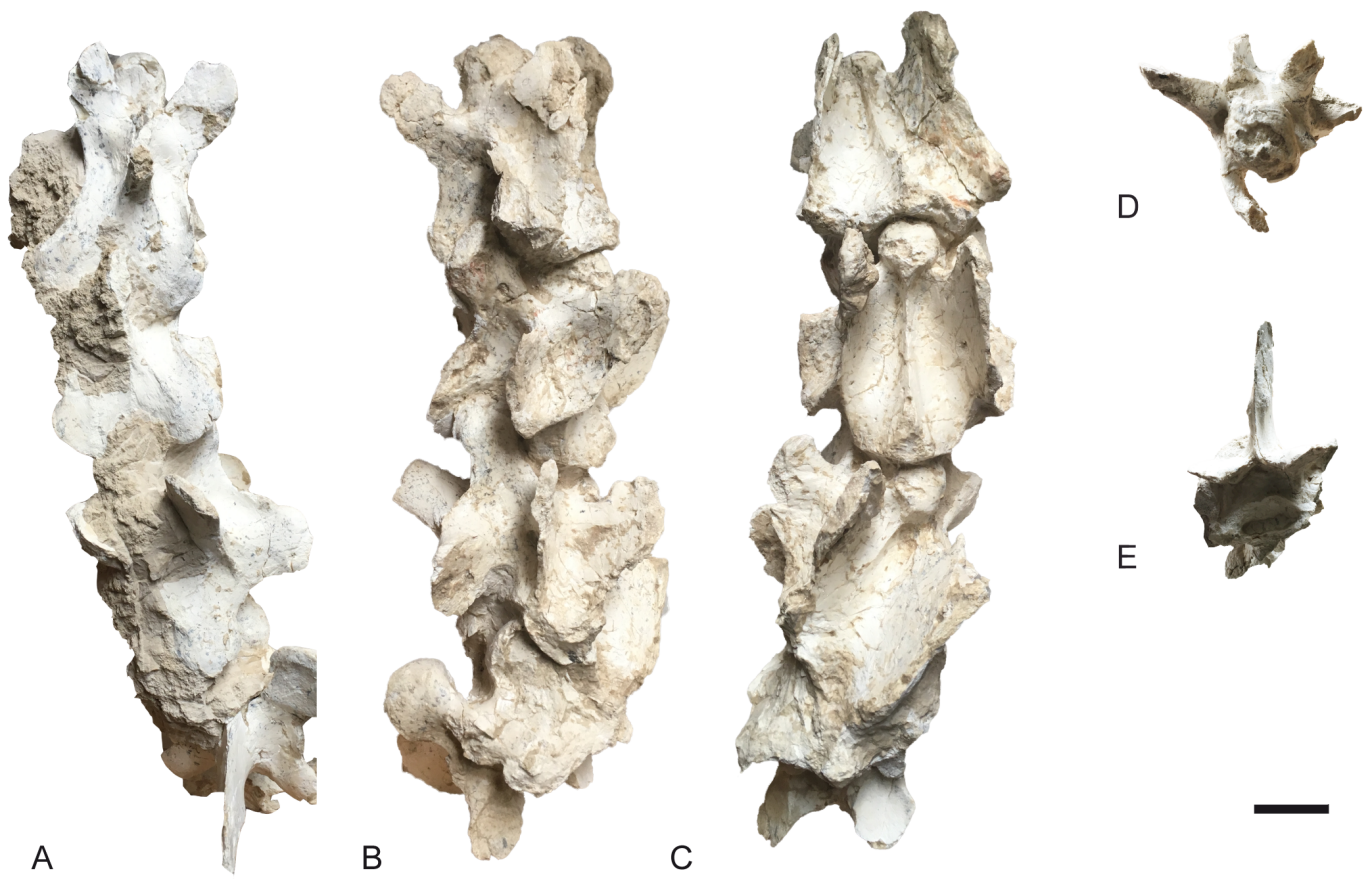
Neck of *Decennatherium rex* sp. nov. from BAT10. A-E, BAT10’14.E2-71-74, C3-C6 in A, dorsal view; B, medial/lateral view; C, ventral view; D, cranial view; E, caudal view. Scale bar equals 5 cm.

**Fig 15:**
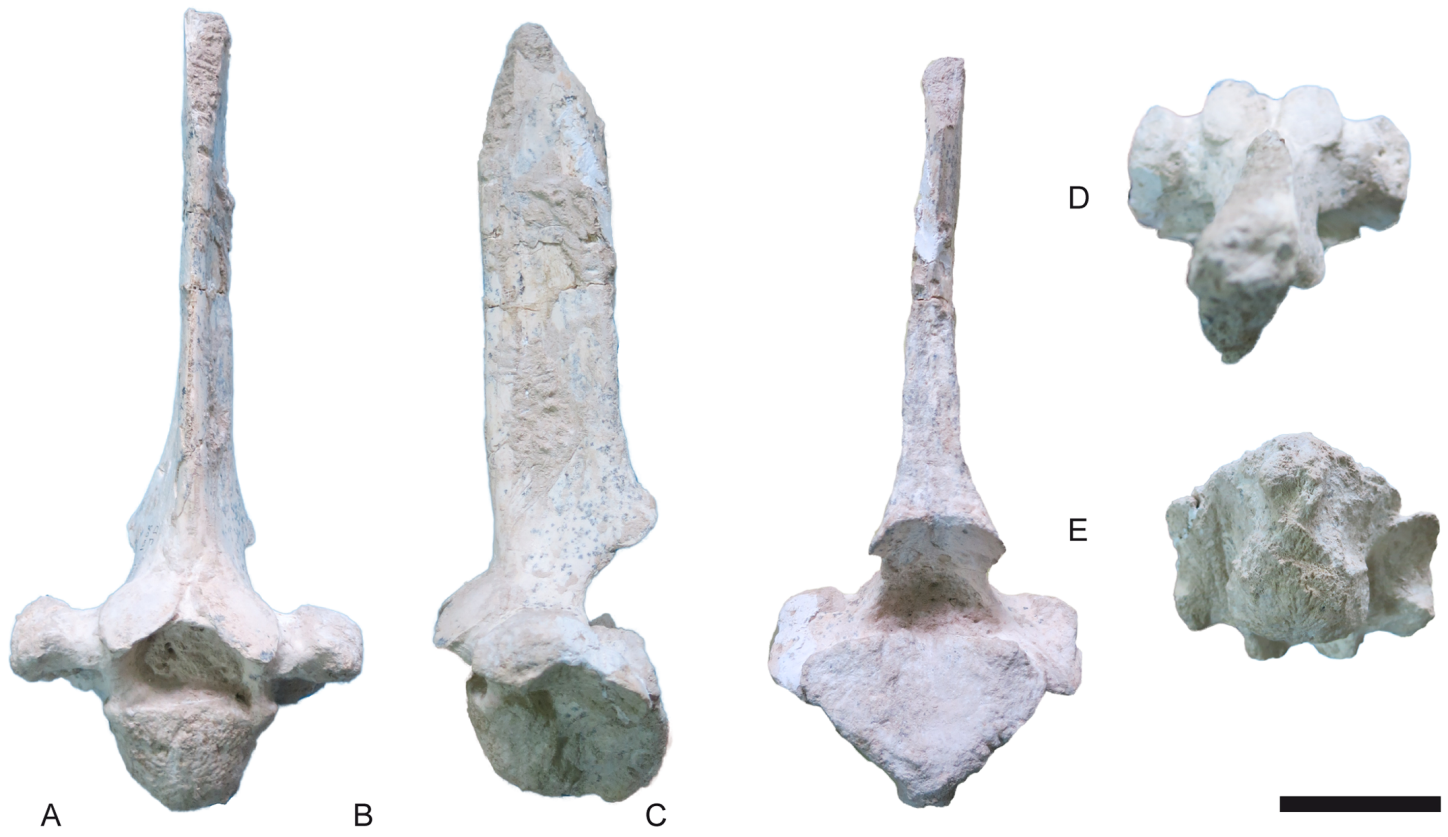
Thoracic vertebrae of *Decennatherium rex* sp. nov. from BAT10. A-E, BAT10’08.D5-10, in A, cranial view; B, medial/lateral view; C, caudal view; D, dorsal view; E, ventral view. Scale bar equals 5 cm.

**Fig 16:**
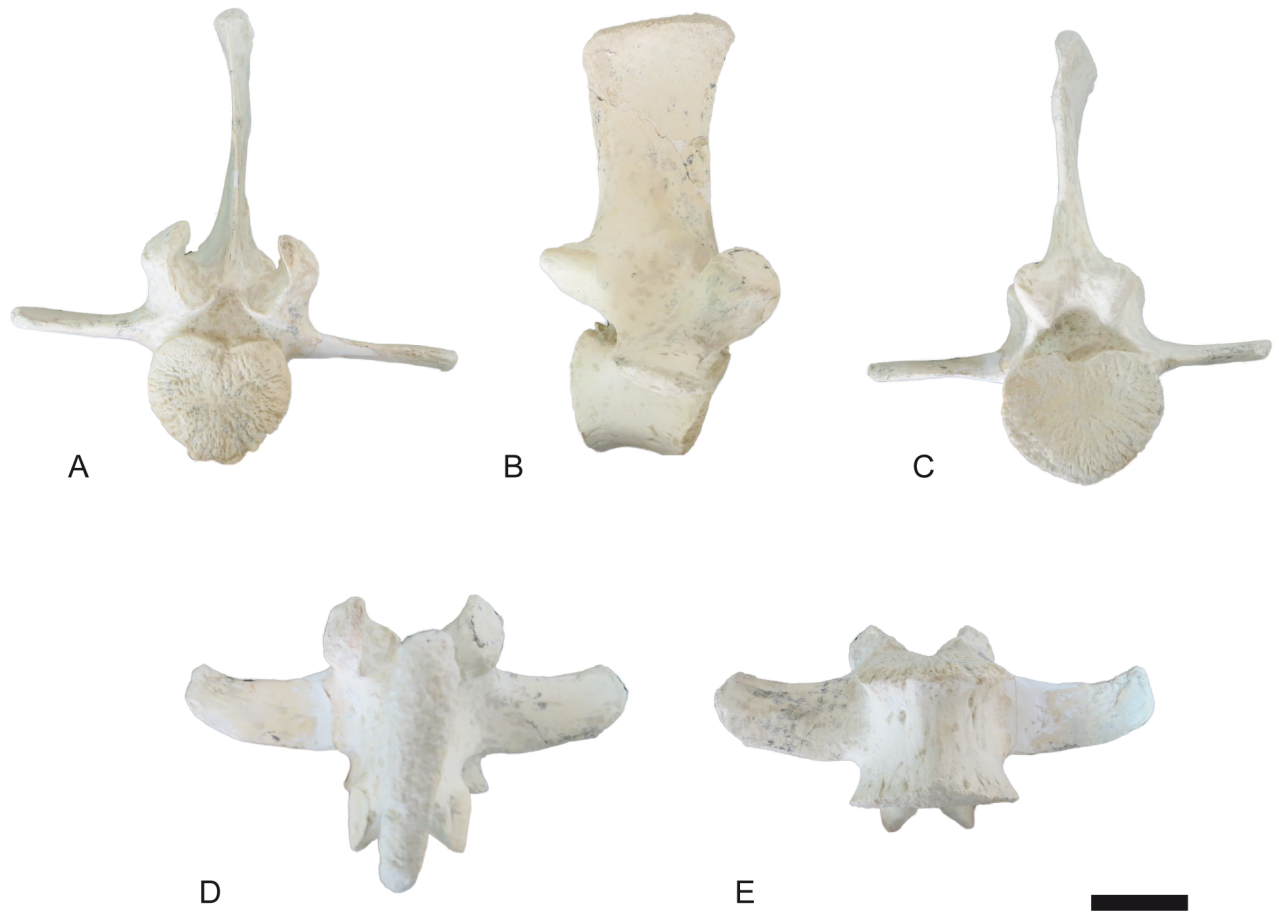
Lumbar vertebrae of *Decennatherium rex* sp. nov. from BAT10. A-E, BAT10’13.D4-40, in A, cranial view; B, medial/lateral view; C, caudal view; D, dorsal view; E, ventral view. Scale bar equals 5 cm.

**Fig 17:**
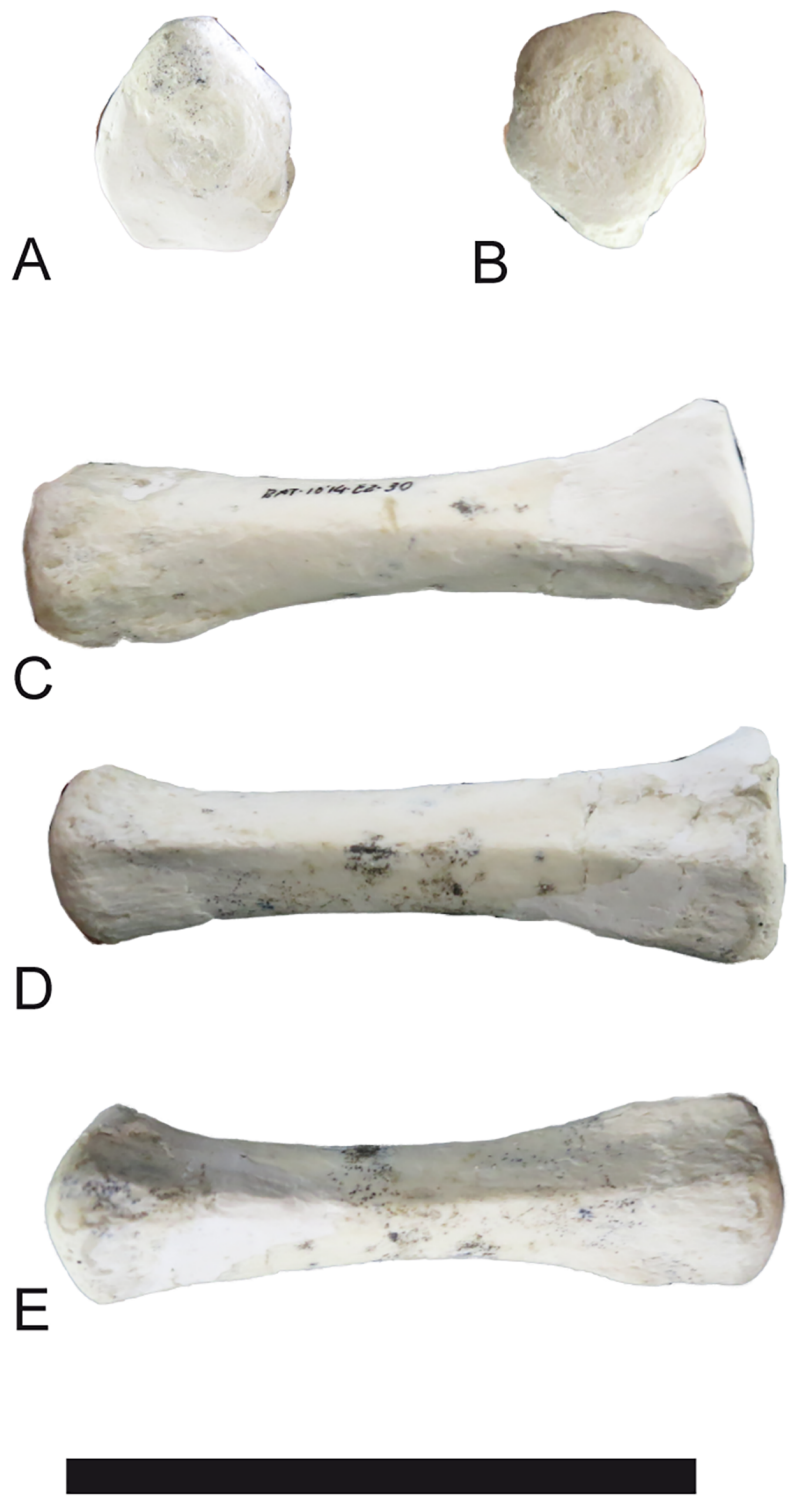
Caudal vertebrae of *Decennatherium rex* sp. nov. from BAT10. A-E, BAT10’14.E2-30, in A, cranial view; B, caudal view; C, medial/lateral view; D, dorsal view; E, ventral view. Scale bar equals 5 cm.

**Fig 18:**
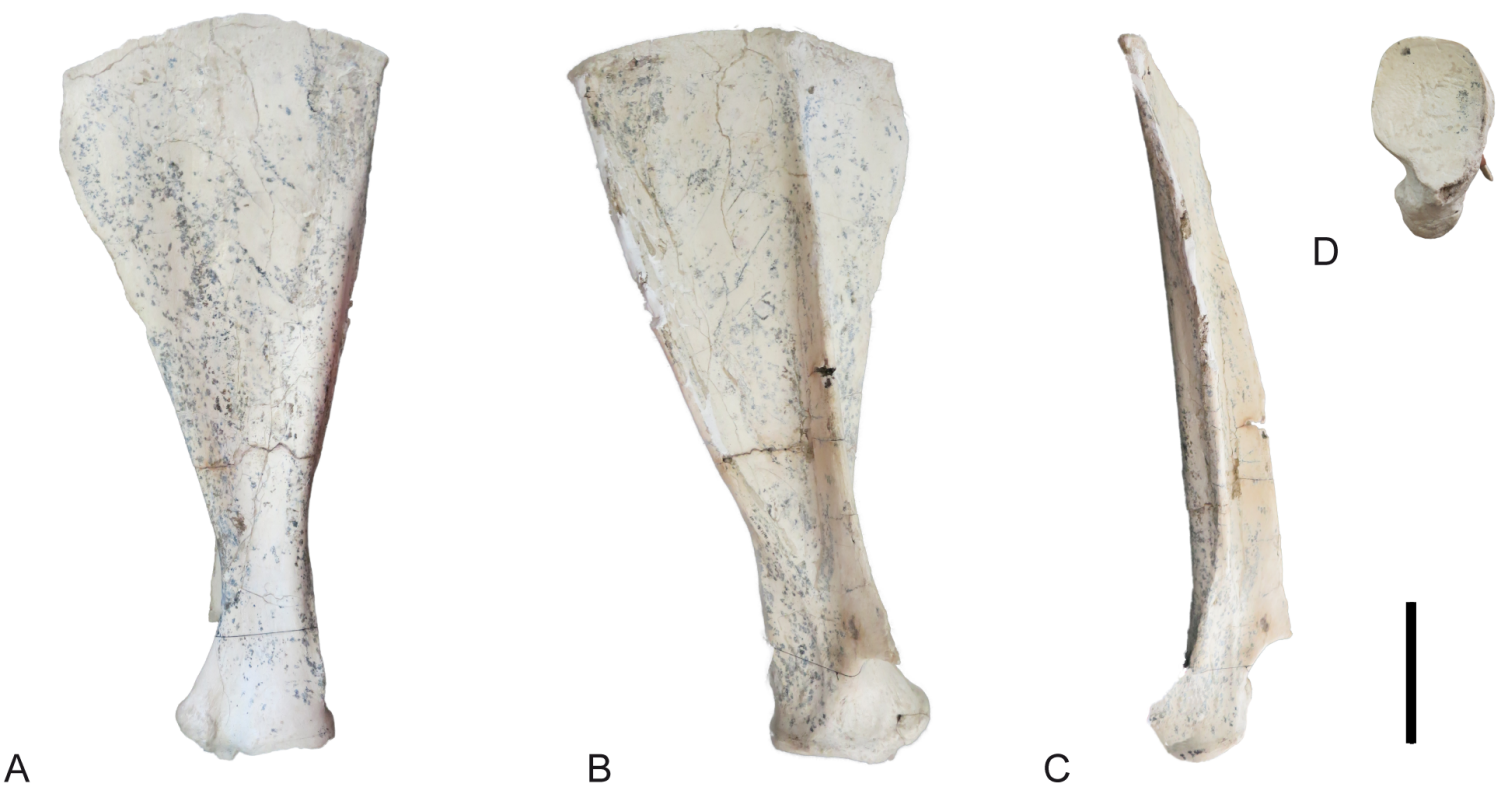
Scapula of *Decennatherium rex* sp. nov. from BAT10. A-D, BAT10’08.F2-51, right scapula in A, medial view; B, lateral view; C, dorsal view; D, distal view; Scale bar equals 10 cm.

**Fig 19:**
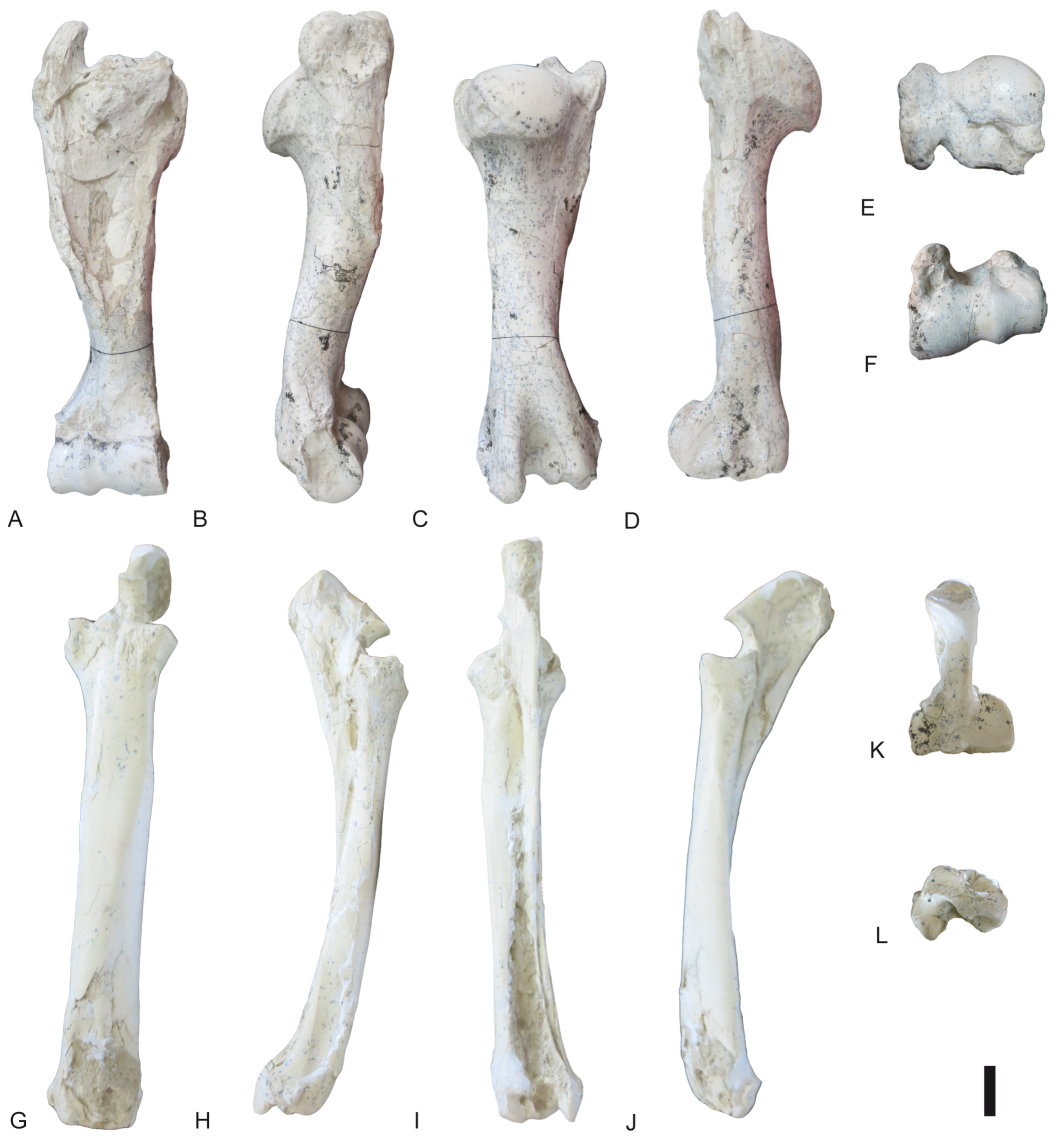
Forelimb of *Decennatherium rex* sp. nov. from BAT10. A-F, BAT10’13.E2-67, right humerus in A, dorsal view; B, lateral view; C, palmar view; D, medial view; E, proximal view; F, distal view; G-L, BAT10’09.G2-104, right radius-ulna in G, dorsal view; H, lateral view; I, palmar view; J, medial view; K, proximal view; L, distal view. Scale bar equals 5 cm.

**Fig 20:**
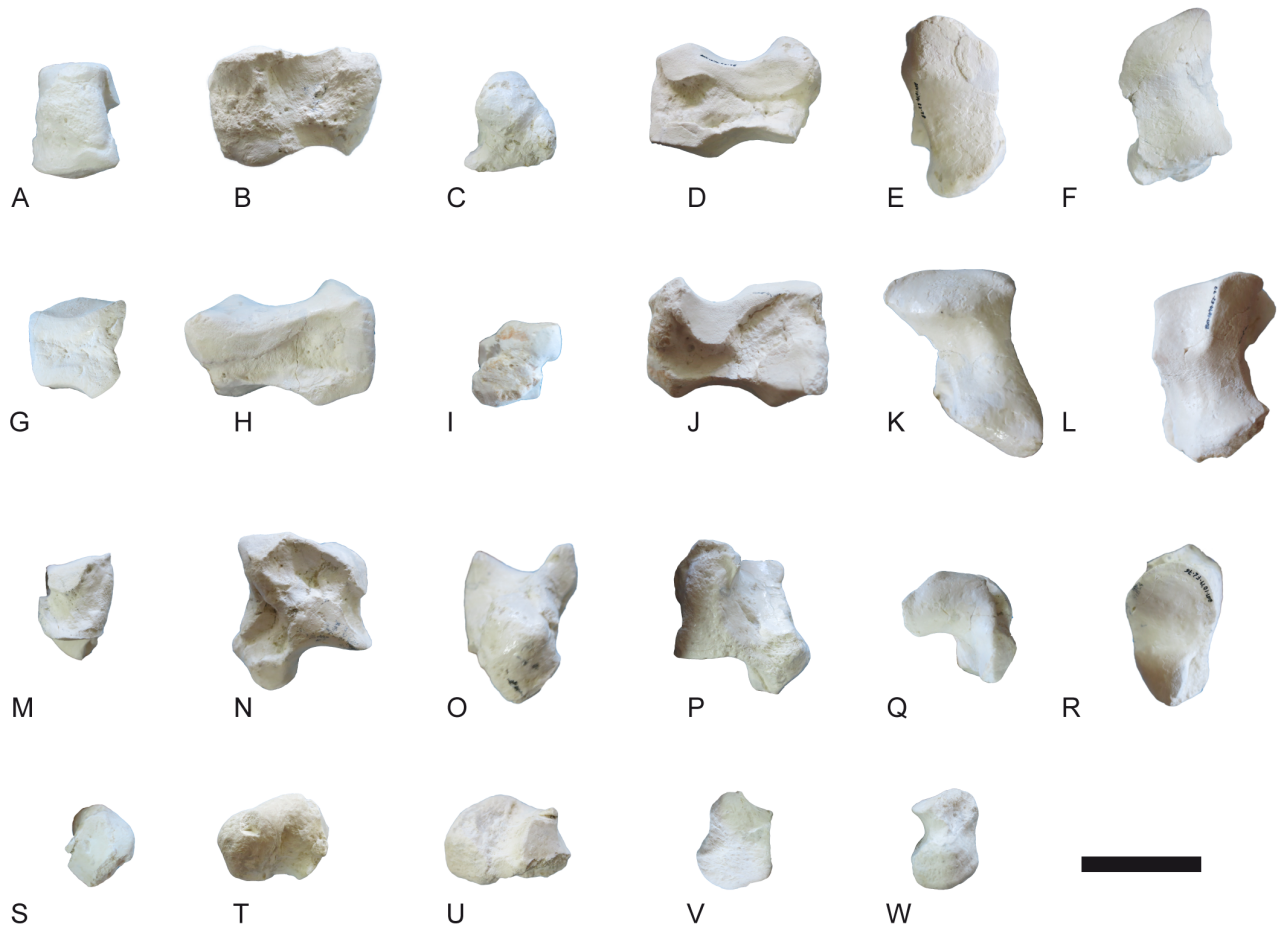
Carpals of *Decennatherium rex* sp. nov. from BAT10. A-F, BAT10’14.E2-78, left scaphoid in in A, dorsal view; B, medial view; C, palmar view; D, lateral view; E, proximal view; F, distal view; G-L, BAT10’14.E2-77, left semilunate in G, dorsal view; H, medial view; I, palmar view; J, lateral view; K, proximal view; L, distal view; M-R, BAT10’14.E2-76, left triquetrum in M, dorsal view; N, medial view; O, palmar view; P, lateral view; Q, proximal view; R, distal view; S-W, BAT10’14.E2-46, left pisiform in S, dorsal view; T, medial view; U, lateral view; V, proximal view; W, distal view. Scale bar equals 5 cm.

**Fig 21:**
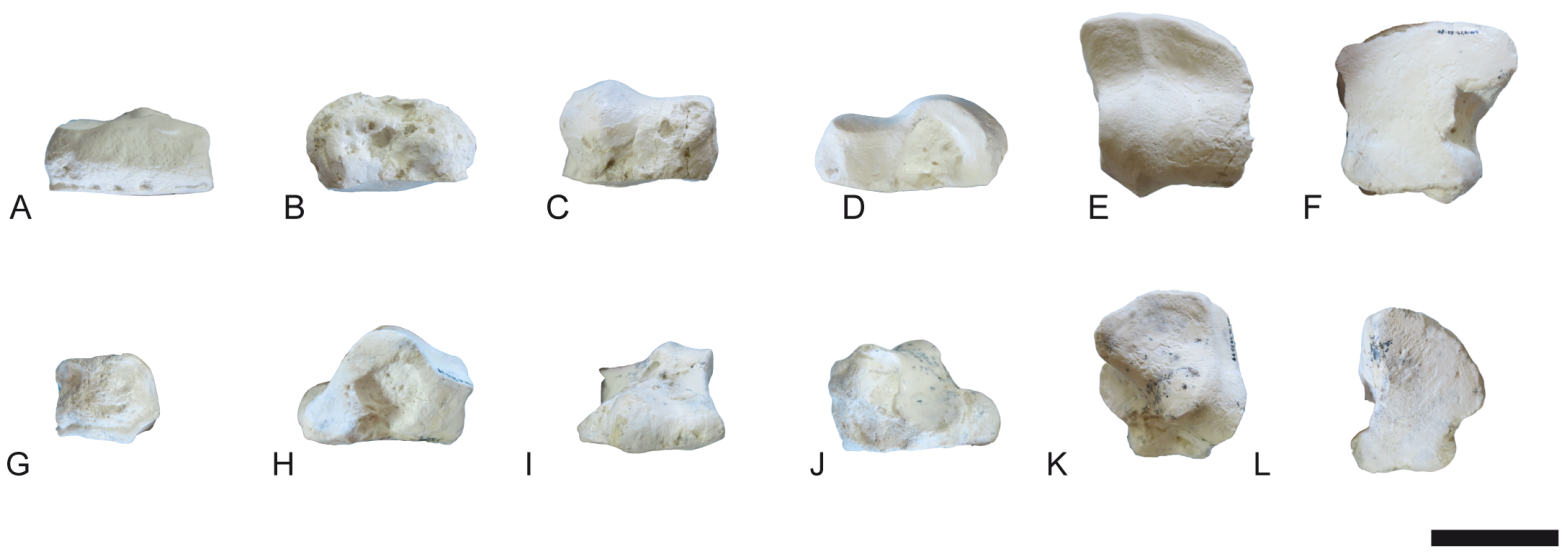
Carpals of *Decennatherium rex* sp. nov. from BAT10. A-F, BAT10’14.E2.80–74, left magnotrapezoid in in A, dorsal view; B, medial view; C, palmar view; D, lateral view; E, proximal view; F, distal view; G-L, BAT10’14.E2-80, left hamatum in G, dorsal view; H, medial view; I, palmar view; J, lateral view; K, proximal view; L, distal view. Scale bar equals 5 cm.

**Fig 22:**
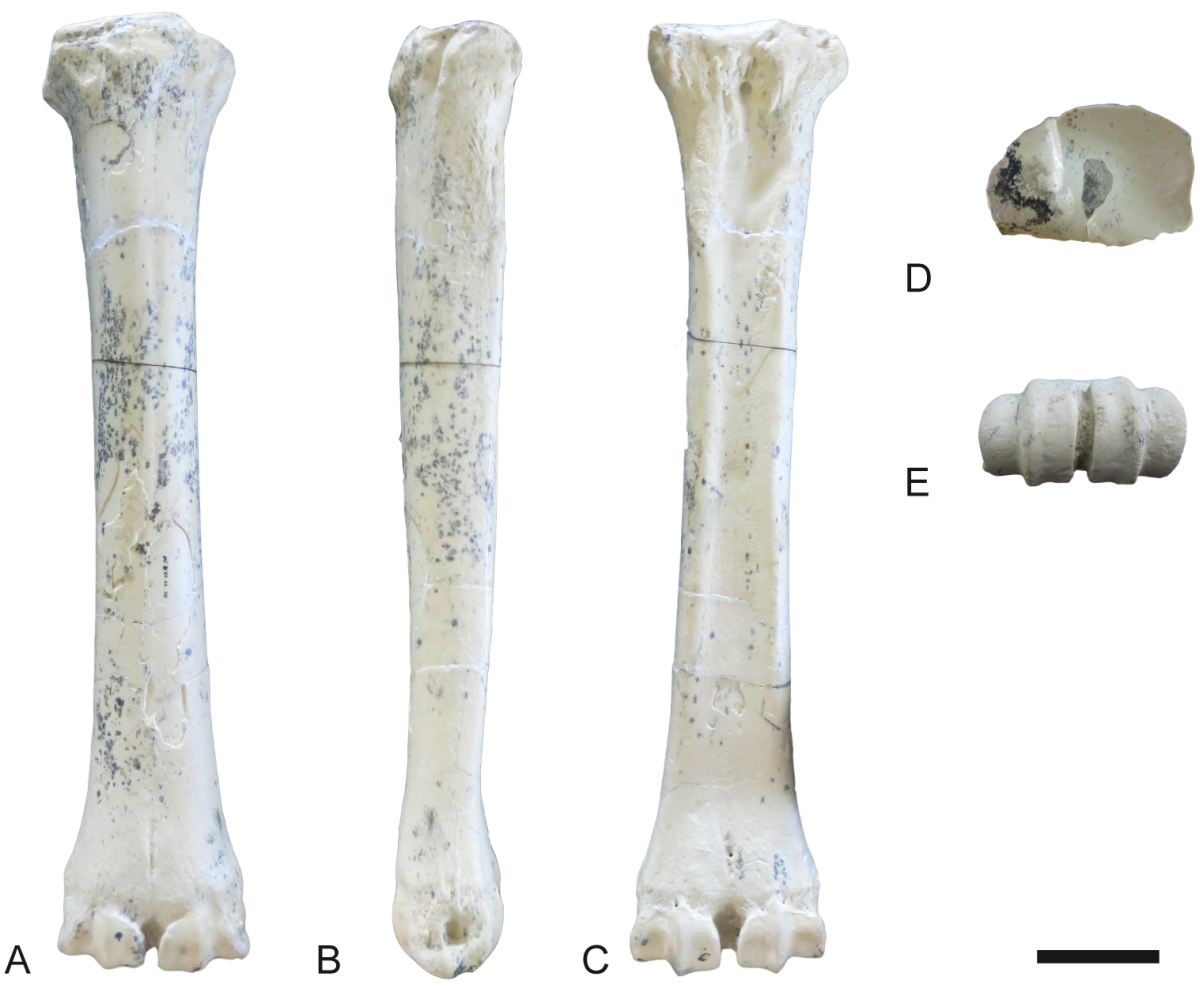
Metacarpal III-IV of *Decennatherium rex* sp. nov. from BAT10. A-E, BAT10’07.G2-28, left metacarpal III-IV in A, dorsal view; B, medial view; C, plantar view; D, proximal view; E, distal view. Scale bar equals 5 cm.

**Fig 23:**
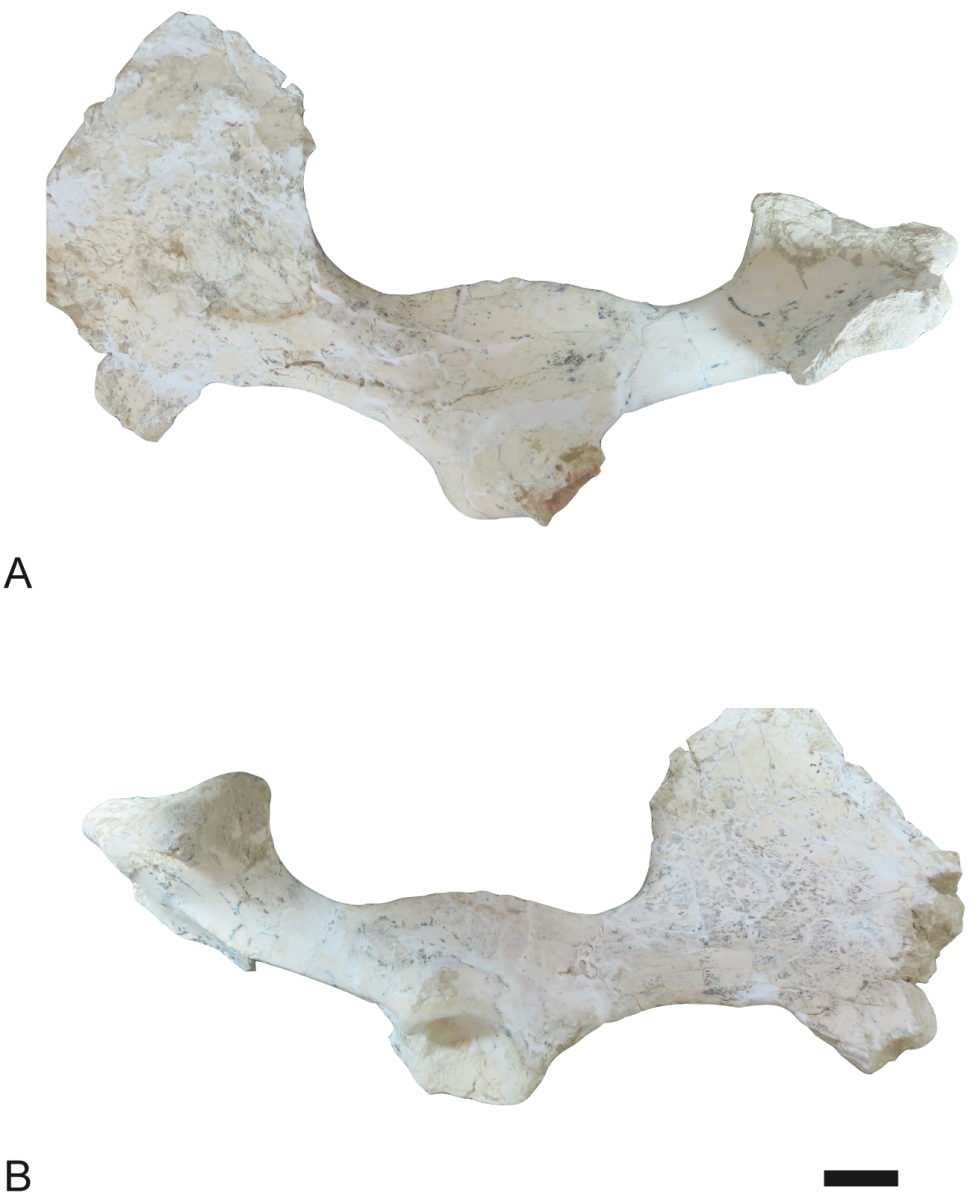
Pelvis of *Decennatherium rex* sp. nov. from BAT10. A-B, BAT10’09.G1-1, right pelvis fragment in A, medial view; B, lateral view. Scale bar equals 5 cm.

**Fig 24:**
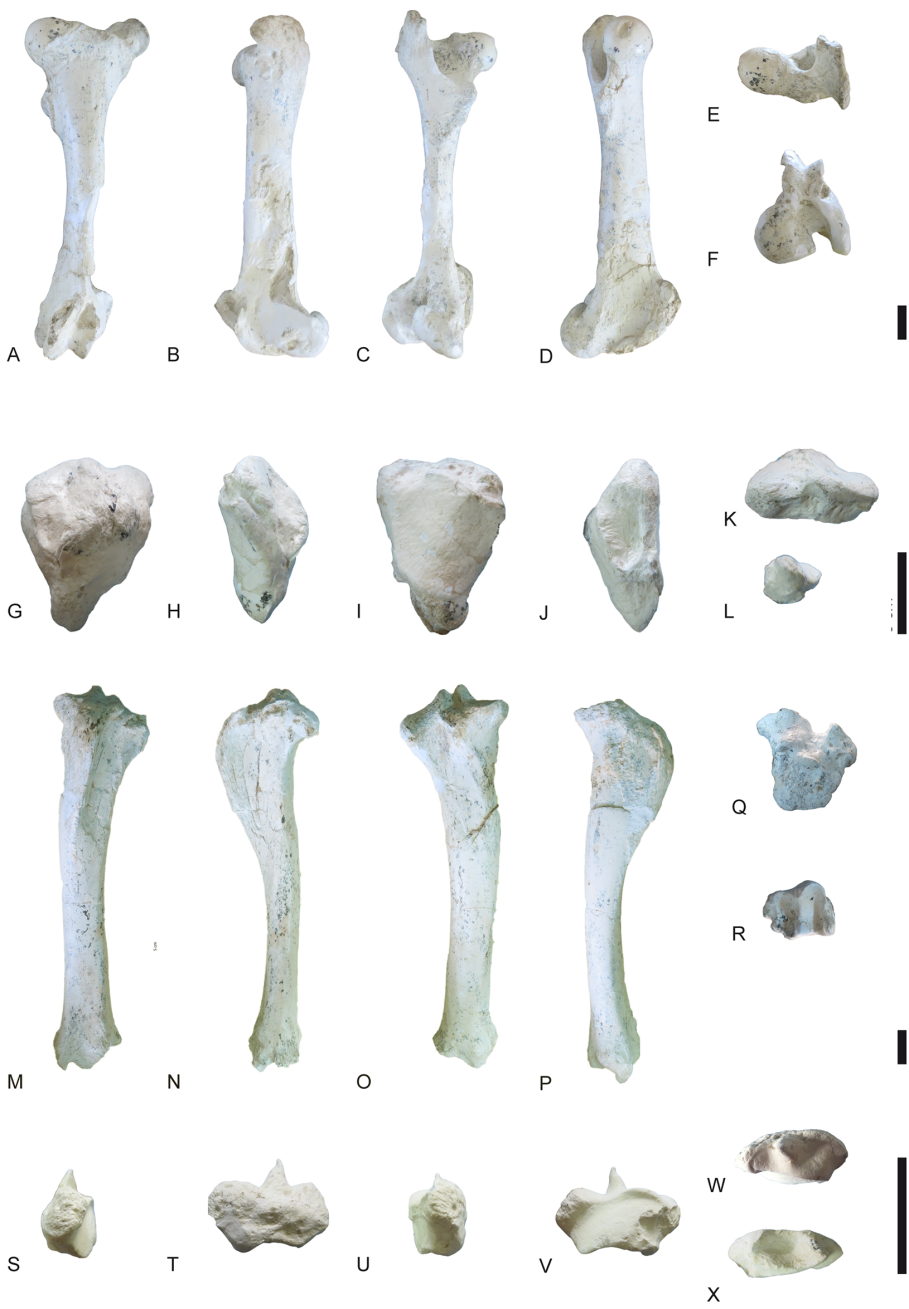
Hindlimb of *Decennatherium rex* sp. nov. from BAT10. A-F, BAT10’14.E2-10, left femur in in A, dorsal view; B, lateral view; C, plantar view; D, medial view; E, proximal view; F, distal view; G-L, BAT10’14.E2-15, right patella in G, dorsal view; H, lateral view; I, plantar view; J, medial view; K, proximal view; L, distal view; M-R, BAT10’14.E2-26, left tibia in M, dorsal view; N, lateral view; O, plantar view; P, medial view; Q, proximal view; R, distal view; S-W, BAT10’14.E2-27, right fibula in S, dorsal view; T, lateral view; U, plantar view; V, medial view; W, proximal view; X, distal view. Scale bar equals 5 cm.

**Fig 25:**
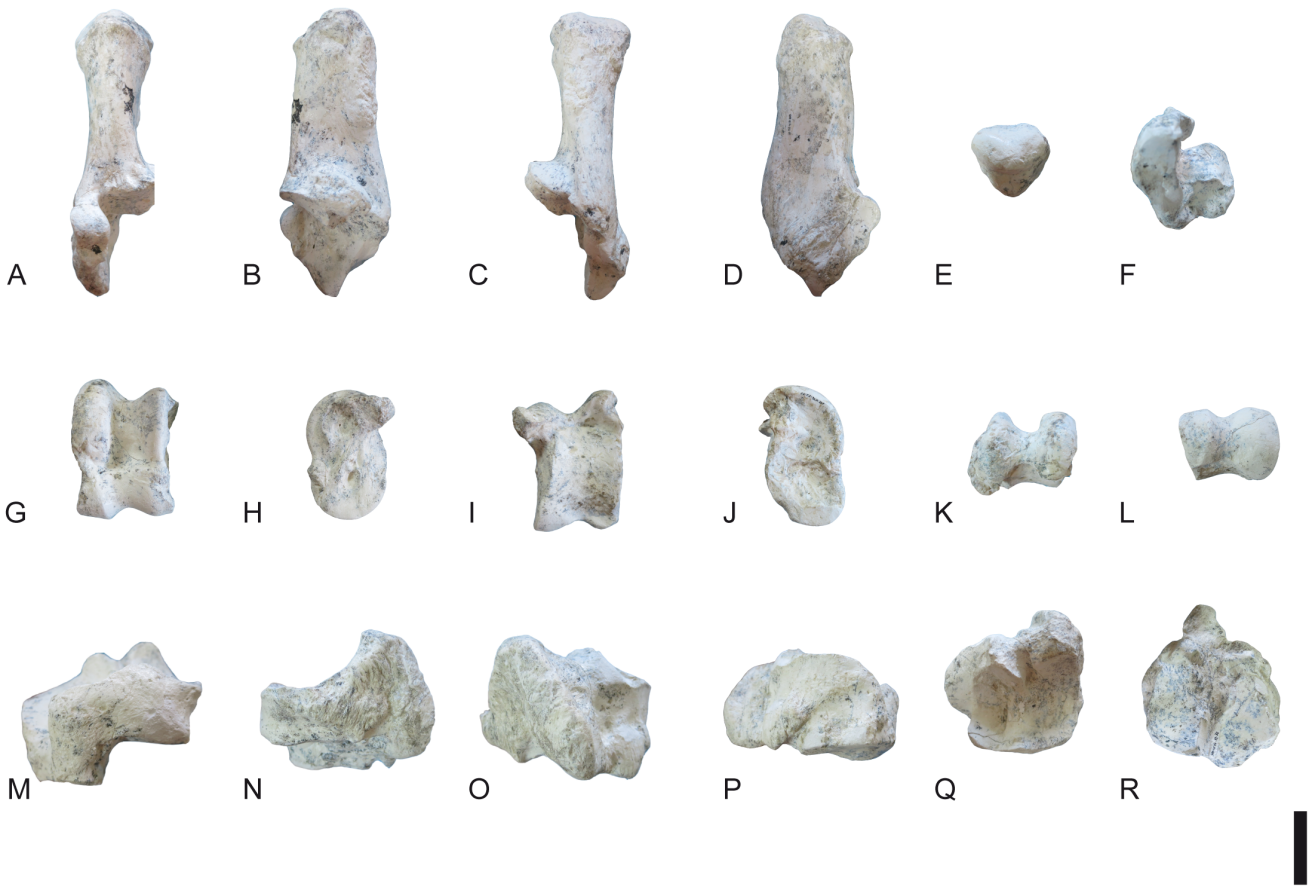
Tarsals of *Decennatherium rex* sp. nov. from BAT10. A-F, BAT10’14.E2-17, right calcaneus in in A, dorsal view; B, medial view; C, plantar view; D, lateral view; E, proximal view; F, distal view; G-L, BAT10’14.E2-23, right astragalus in G, dorsal view; H, medial view; I, plantar view; J, lateral view; K, proximal view; L, distal view; M-R, BAT10’14.E2-32, rightnavicular-cuboid in M, dorsal view; N, medial view; O, plantar view; P, lateral view; Q, proximal view; R, distal view. Scale bar equals 5 cm.

**Fig 26:**
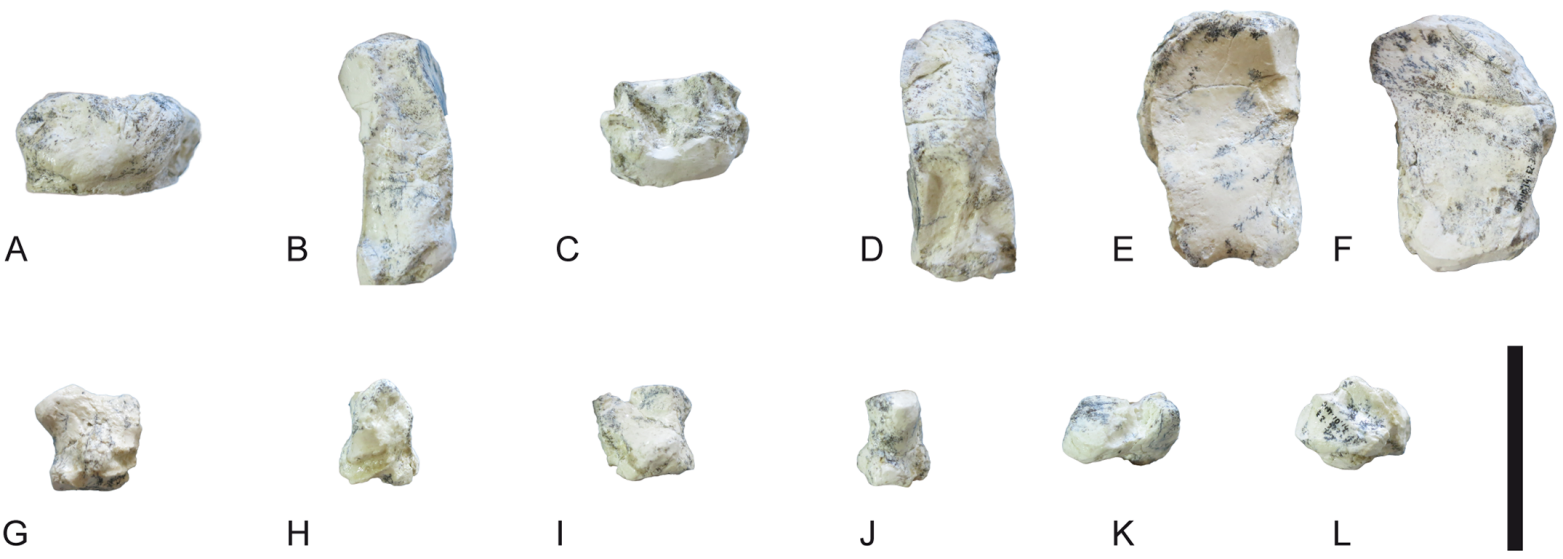
Tarsals of *Decennatherium rex* sp. nov. from BAT10. A-F, BAT10’14.E2-38, right ectomesocuneiform in in A, dorsal view; B, medial view; C, plantar view; D, lateral view; E, proximal view; F, distal view; G-L, BAT10’14.E2-37, right entocuneiform in G, dorsal view; H, medial view; I, plantar view; J, lateral view; K, proximal view; L, distal view. Scale bar equals 5 cm.

**Fig 27:**
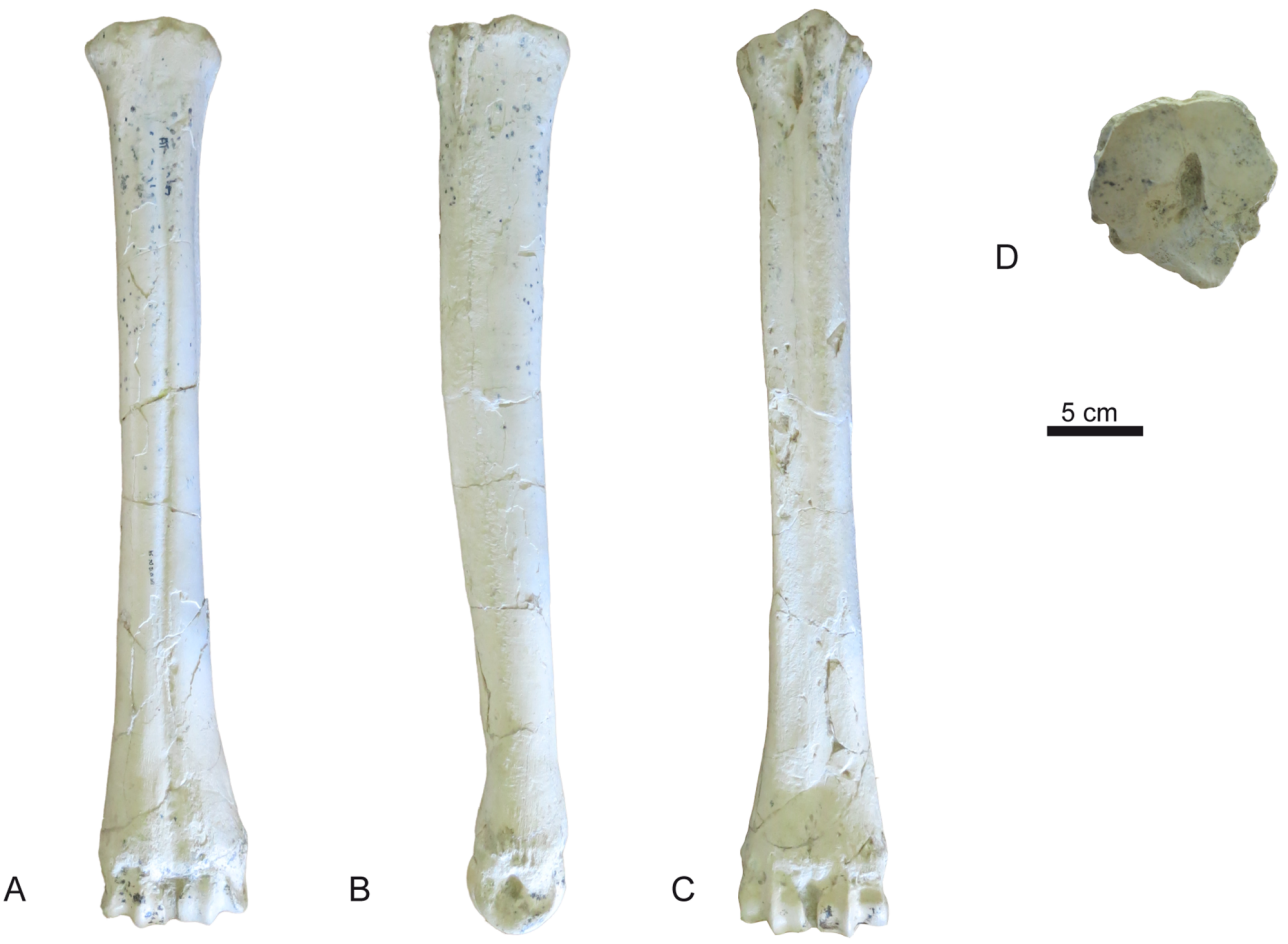
Metatarsal III-IV of *Decennatherium rex* sp. nov. from BAT10. A-D, BAT10’12.D6-54, right metatarsal III-IV in A, dorsal view; B, medial view; C, plantar view; D, proximal view. Scale bar equals 5 cm.

**Fig 28:**
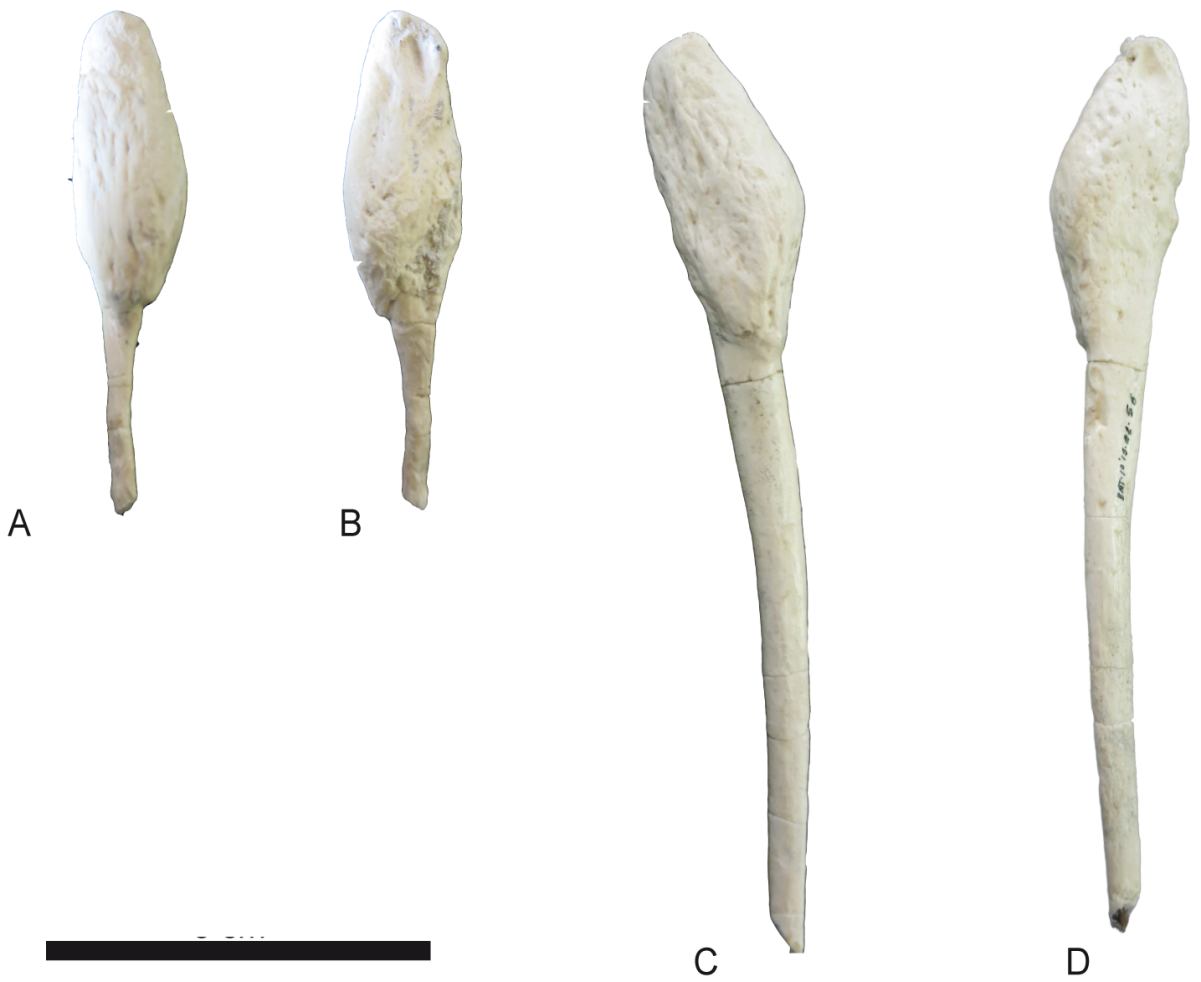
Lateral metapodials of *Decennatherium rex* sp. nov. from BAT10. A-B, BAT10’12.D6-108, in A, dorsal view; B, plantar view; C-D, BAT10’13.E2-56 in A, lateral view; B, medial view. Scale bar equals 5 cm.

**Fig 29:**
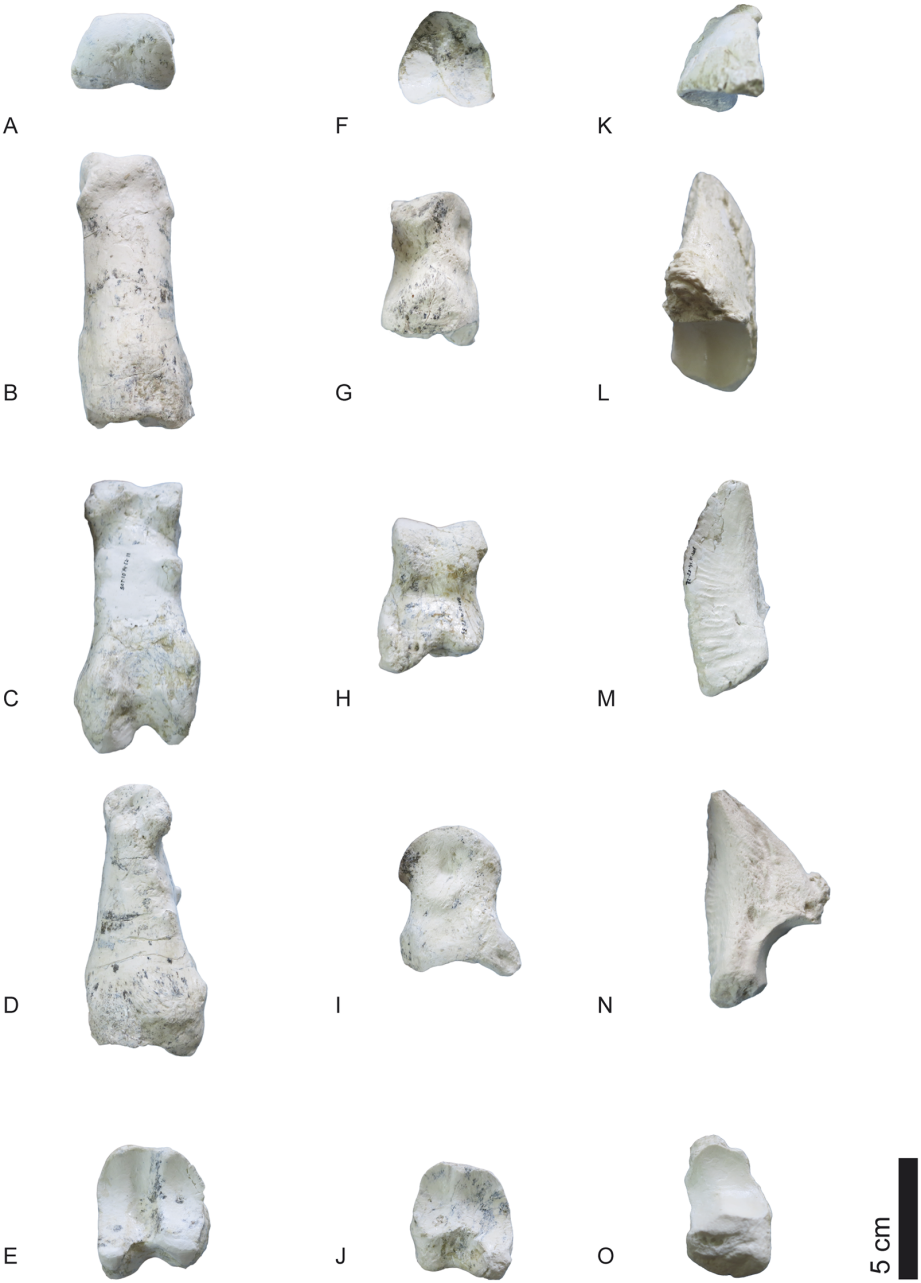
Phalanges of *Decennatherium rex* sp. nov. from BAT10. A-E, BAT10’14.E2-11, first phalanx of the right digit in A, distal view; B, dorsal view; C, palmar/plantar view; D, exterior view; E, proximal view; F-J, BAT10’14.E2-33, second phalanx of the right digit in F, distal view; G, dorsal view; H, palmar/plantar view; I, exterior view; J, proximal view; K-O, BAT10’14.E2-22, third phalanx of the right digit in K, distal view; L, dorsal view; M, palmar/plantar view; N, exterior view; O, proximal view; Scale bar equals 5 cm.

**Fig 30:**
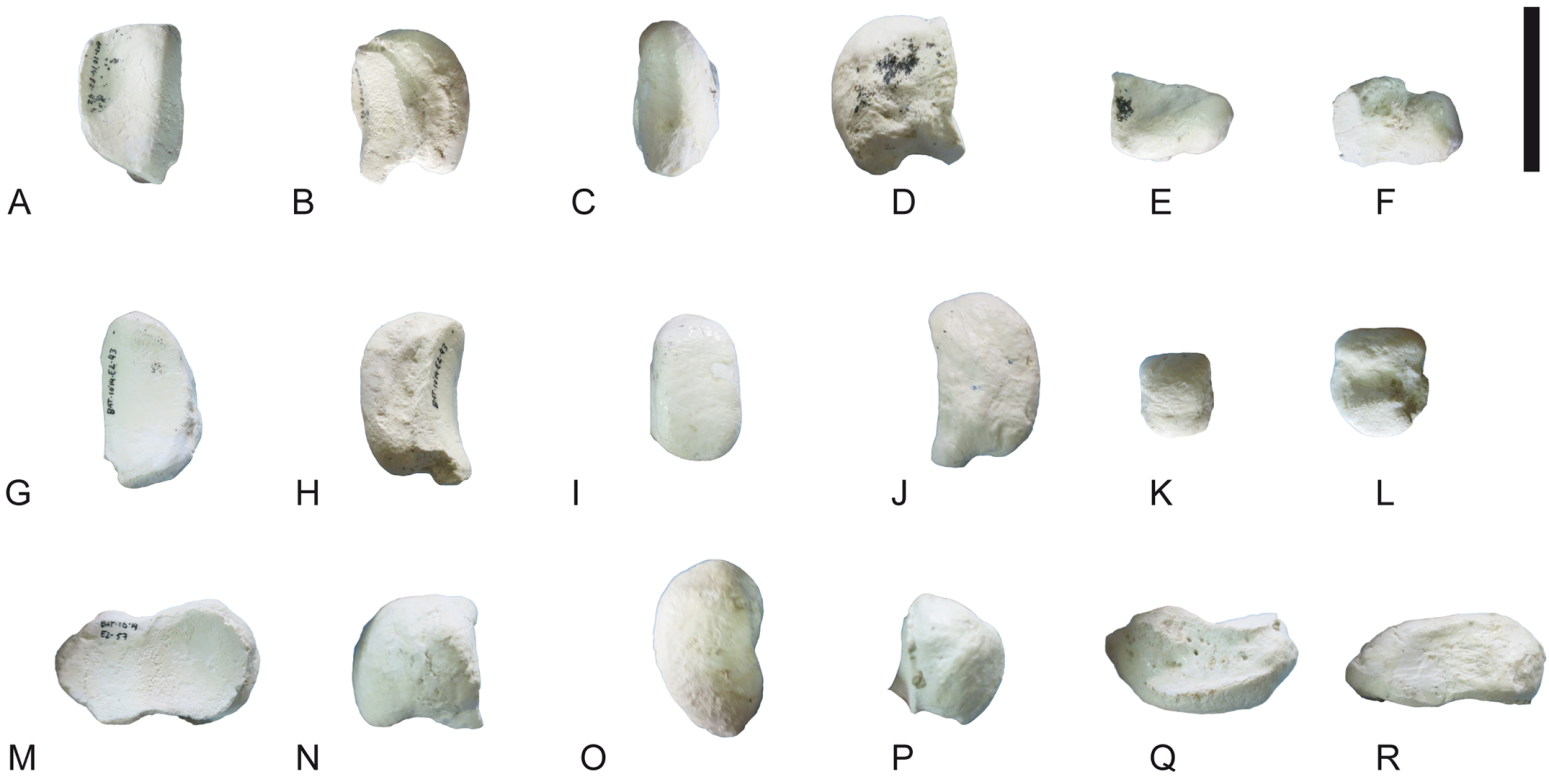
Sesamoids of *Decennatherium rex* sp. nov. from BAT10. A-F, BAT10’14.E2-42, proximal exterior sesamoid in A, cranial view; B, lateral view; C, caudal view; D, medial view; E, dorsal view; F, palmar/plantar view; G-L; BAT10’14.E2-43, proximal interdigital sesamoid in G, cranial view; H, lateral view; I, caudal view; J, medial view; K, dorsal view; L, palmar/plantar view; M-R; BAT10’14.E2-57, distal sesamoid in M, cranial view; N, lateral view; O, caudal view; P, medial view; Q, dorsal view; R, palmar/plantar view. Scale bar equals 5 cm.

**Fig 31:**
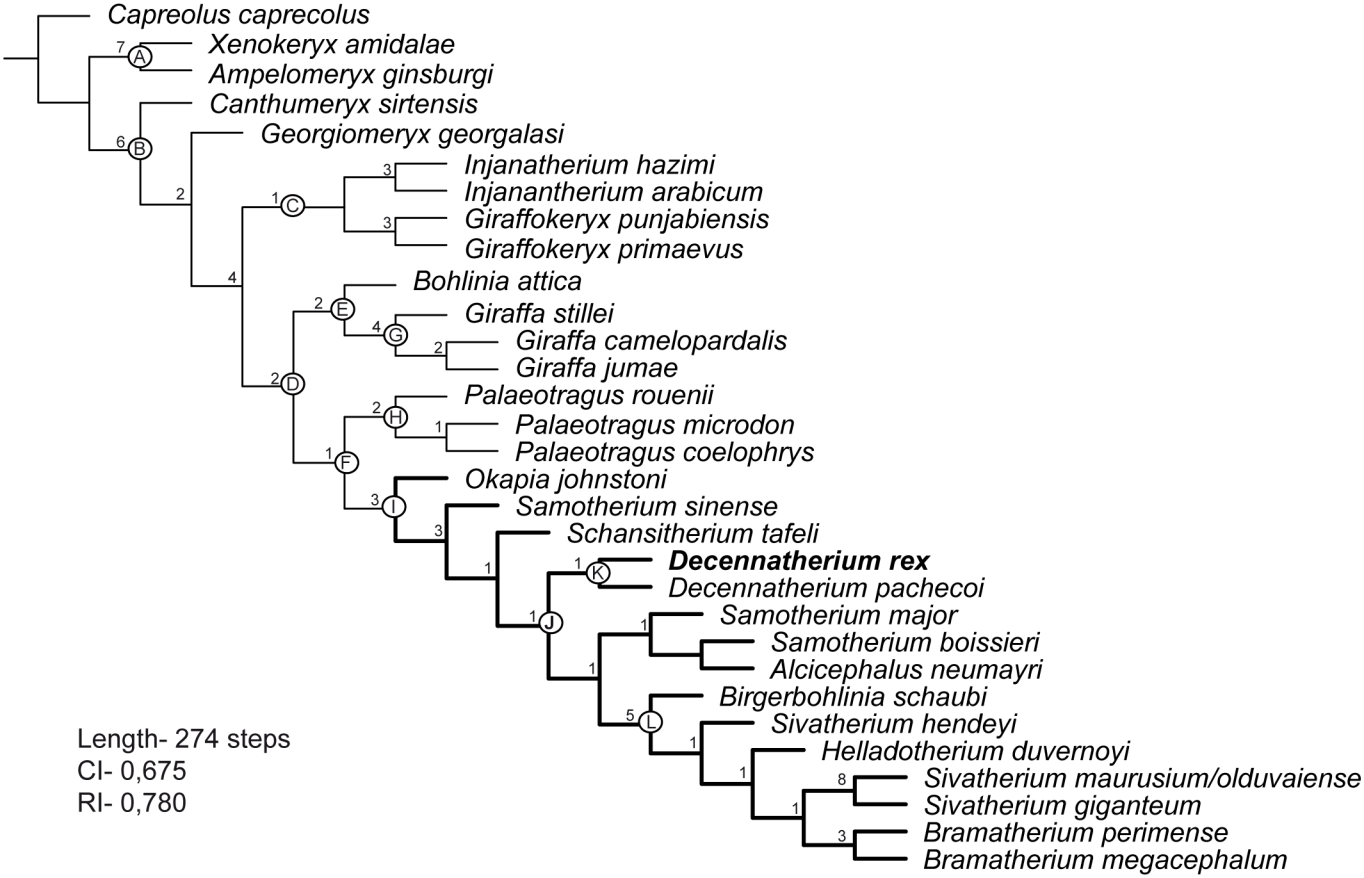
Phylogenetic relationships among giraffids. MPT of 277 steps (CI = 0,671; RI = 0,780). Bremer values over each node.

**Fig 32:**
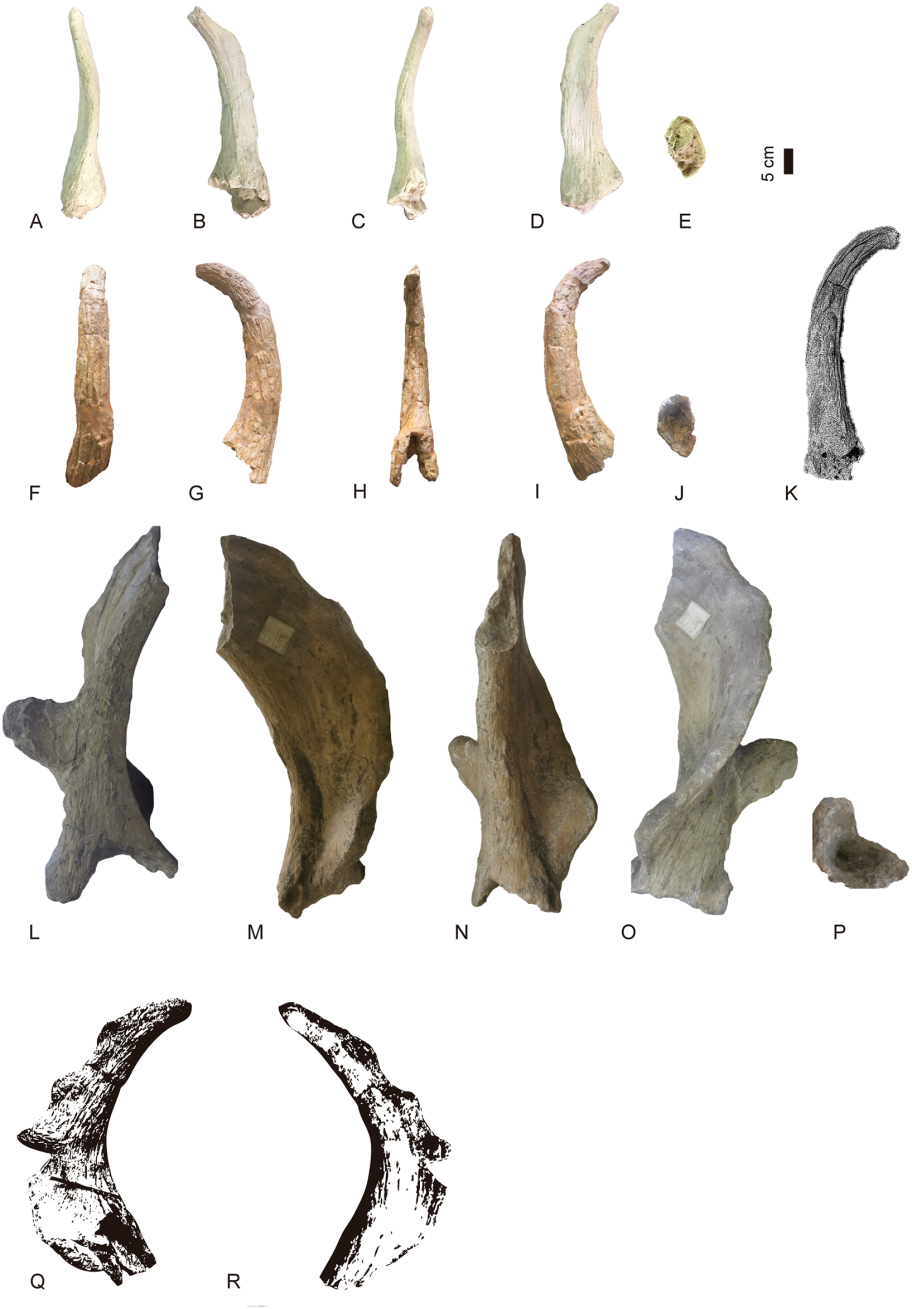
Ossicones of several giraffid species. A-E, BAT10’16.D4-20, right posterior ossicone of *Decennatherium rex* in A, cranial view; B, lateral view; C, caudal view; D, medial view; E, proximal view; F-J, MGUV-7816,right posterior ossicone of *Birgerbohlinia schaubi* in F, cranial view; G, lateral view; H, caudal view; I, medial view; J, proximal view; K, SAM-PQ-L12730, left posterior ossicone of *Sivatherium hendeyi* in lateral view ([63]: Fig 2A, holotype); L-P, BM-39525 left posterior ossicone of *Sivatherium giganteum* in L, cranial view; M, medial view; N, caudal view; O, lateral view; P, proximal view; Q-R, M14955, illustration of the left posterior ossicone of *Sivatherium olduvaiense* in Q, posterolateral view; R, anteromedial view ([62]: Plate IIIa, b.). Scale bar equals 5 cm.

**Fig 33:**
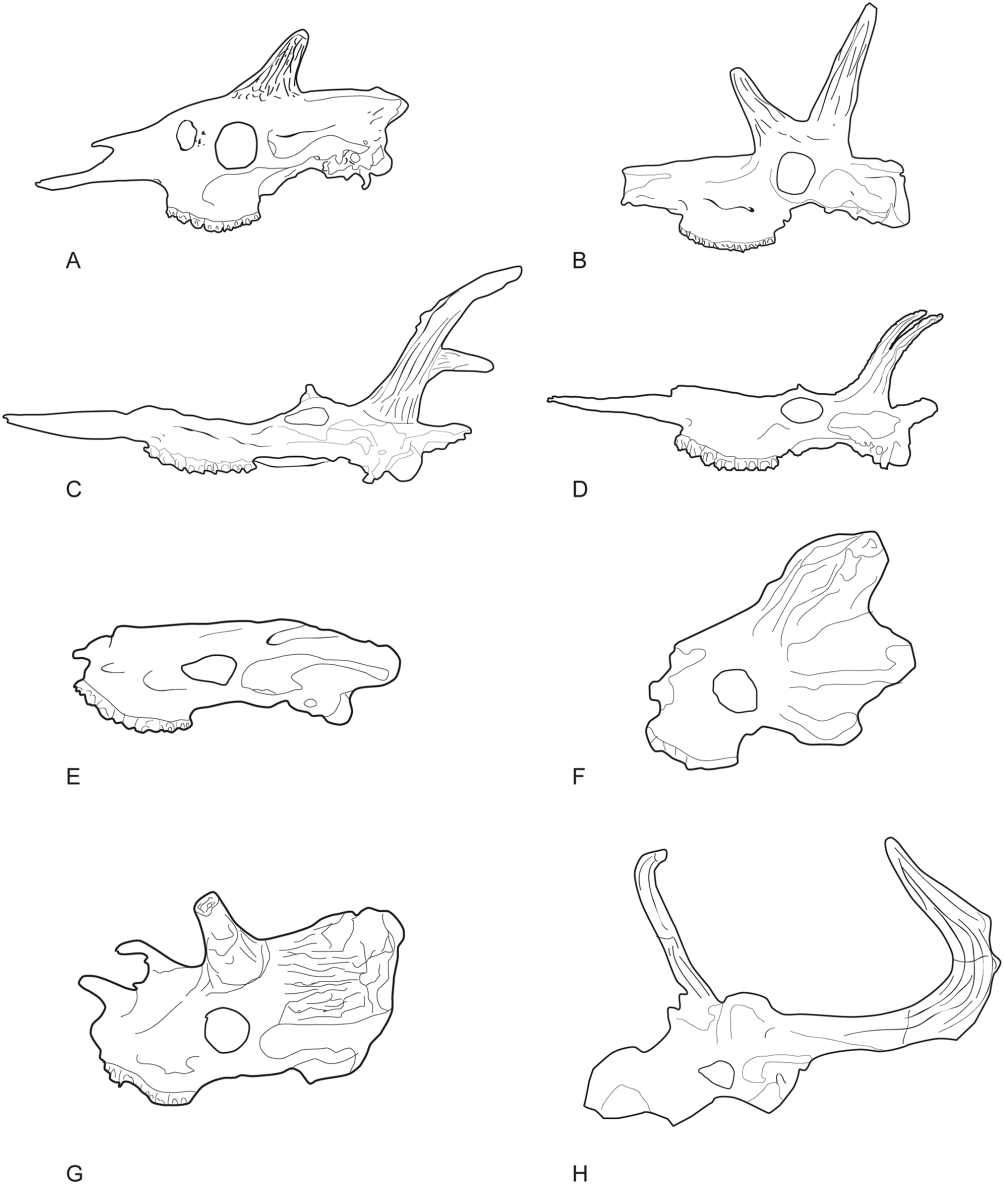
Lateral views of the skulls of the giraffid species included in the analysis. A, MNCN-5226, *Okapia johnstoni*; B, AMNH/WN, *Schansitherium tafeli*; C, BAT10’13.E2-69, *Decennatherium rex*; D, BAT10’08.G3-91, *Decennatherium rex*; E, PIK-1500, *Helladotherium duvernoyi*; F, AM-27016, *Bramatherium megacephalum* M26675-P80, G, NHM-15283, *Sivatherium giganteum*; H, ANK-II-127 ([91]: Fig 1), *Sivatherium maurusium*. Images not to scale.

**Fig 34:**
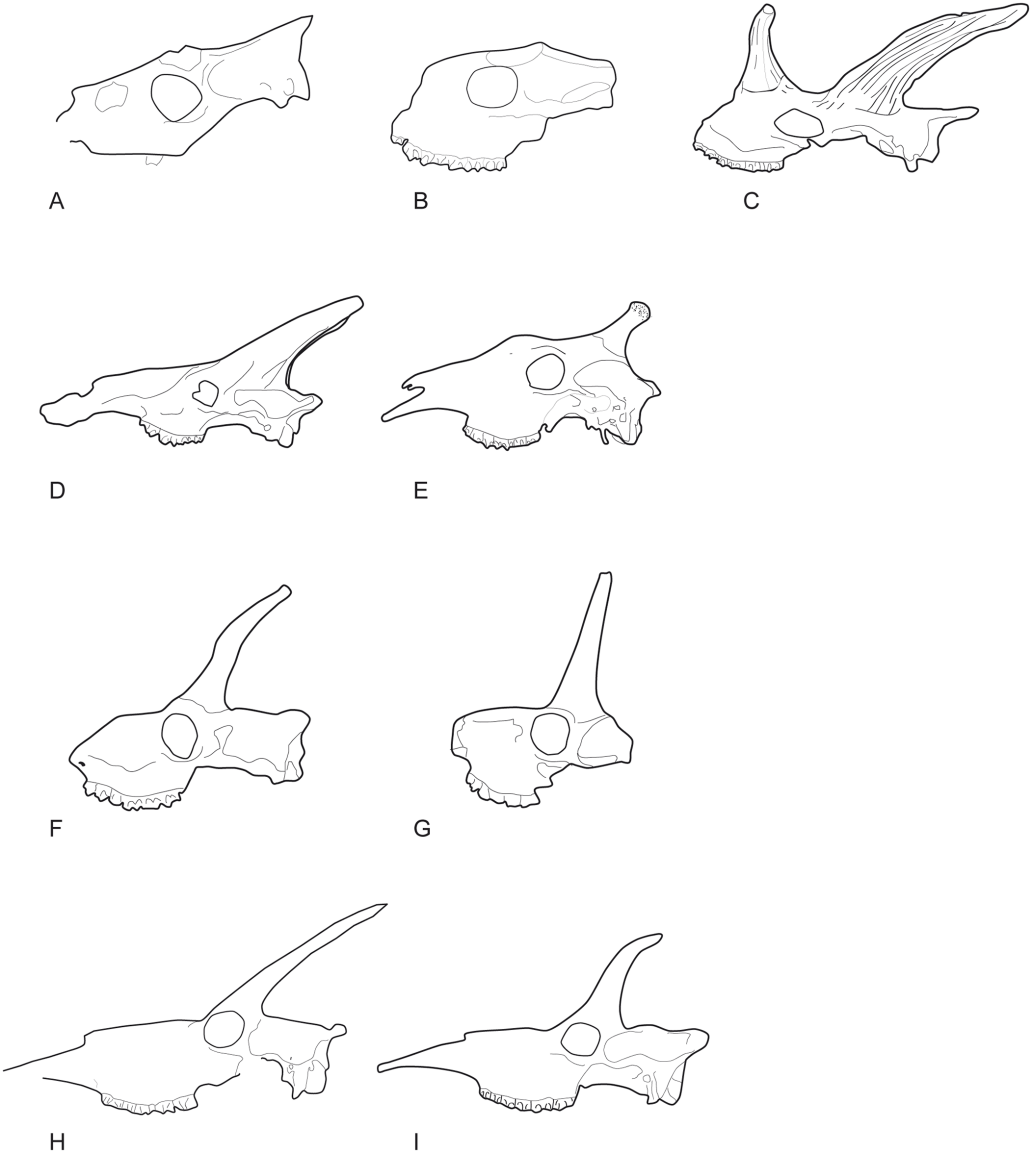
Lateral views of the skulls of the giraffid species included in the analysis. A, M 26670, *Canthumeryx sirtensis*; B, THB-30 ([12]: Textfig. 1), *Gerogiomeryx georgalasi*; C, AMNH-19475, *Giraffokeryx punjabiensis*; D, OLD 63 EF HR, *Giraffa jumae* [92]; E, MNCN-3439, *Giraffa camelopardalis*; F, AMNH-10452, *Palaeotragus rouenii*; G, M1124, *Palaeotragus microdon*; H, *Samotherium major* ([1]: Fig 21.5b); I, *Samotherium boissieri* ([1]: Fig 21.5c). Images not to scale.

**Fig 35:**
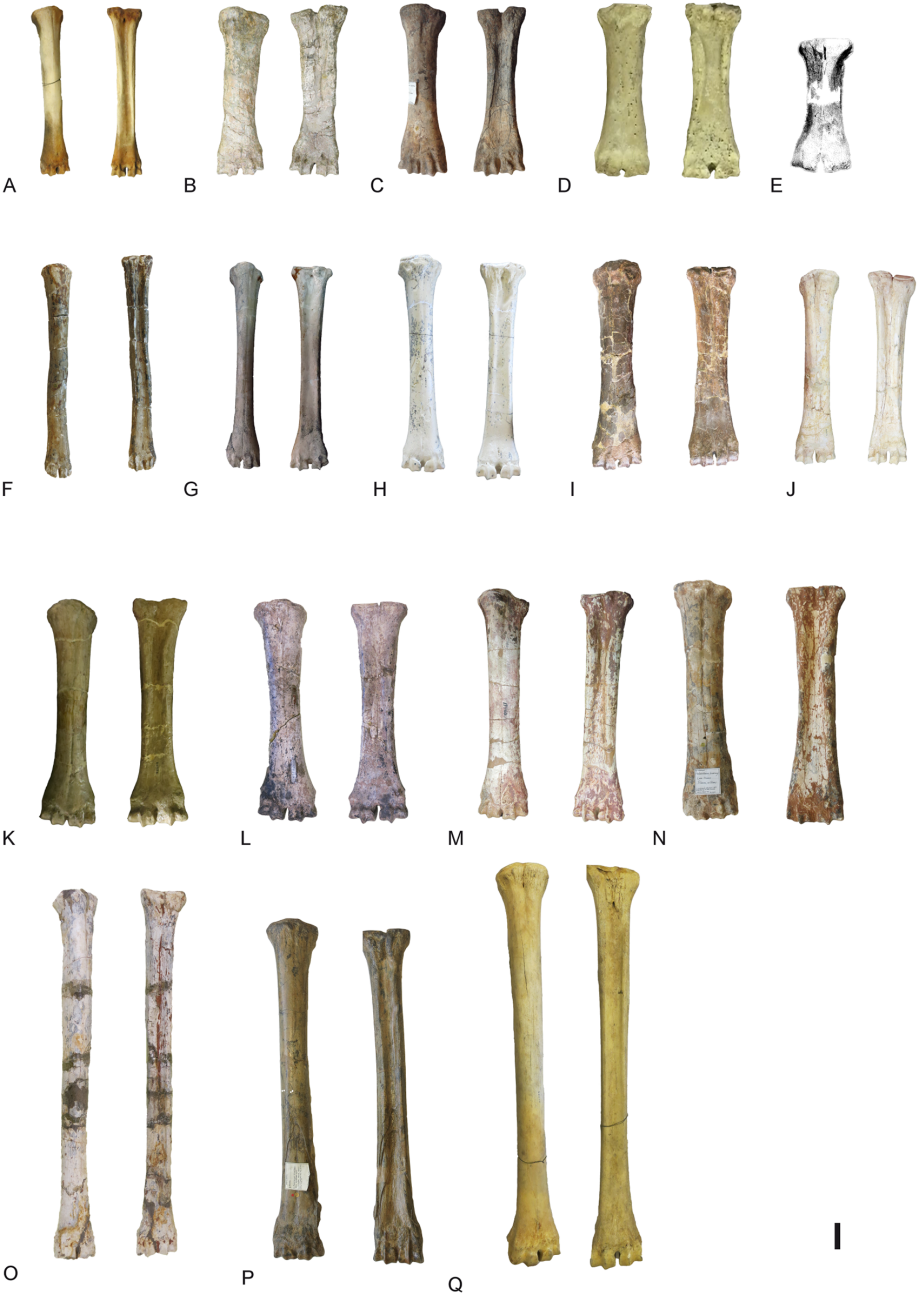
Metacarpals of the giraffid species included in the analysis. A, AMNH-51198, left and right metacarpals of *Okapia johnstoni* in dorsal and palmar views respectively; B, AM-948, left metacarpal of *Sivatherium giganteum* in dorsal and palmar views; C, BM-17102a, right metacarpal of *Sivatherium giganteum* in dorsal and palmar views; D, PQ-L45066, right metacarpal of *Siatherium hendeyi* in dorsal and palmar views; E, right metacarpal of *Sivatherium maurusium* in dorsal view ([64]: Fig. 7c); F, PIK-2756, left metacarpal of *Palaeotragus rouenii* in dorsal and palmar views; G, MNCN-42769, left metacarpal of *Decennatherium pachecoi* in dorsal and palmar views; H, BAT10’07.G2-28, left metacarpal of *Decennatherium rex* in dorsal and palmar views; I, MGUV-7806, right metacarpal of *Birgerbohlinia schaubi* in dorsal and palmar views; J, AM-26349, left metacarpal of *S*. *sinense* in dorsal and palmar views; K, AMNH-20758, left metacarpal of *Samotherium boissieri* in dorsal and palmar views; L, left metacarpal of *Alcicephalus neumayri* in dorsal and palmar views; M, AM-19460, left metacarpal of *Bramatherium megacephalum* in dorsal and palmar views; N, M11377, left metacarpal of *Helladotherium duvernoyi* in dorsal and palmar views; O, PIK-2756, left metacarpal of *Bohlinia attica* in dorsal and palmar views; P, M14957, left metacarpal of *Giraffa jumae* in dorsal and palmar views; Q, AMNH-53543, right and left metacarpals of *Giraffa camelopardalis* in dorsal and palmar views respectively. Scale bar equals 5 cm.

**Fig 36:**
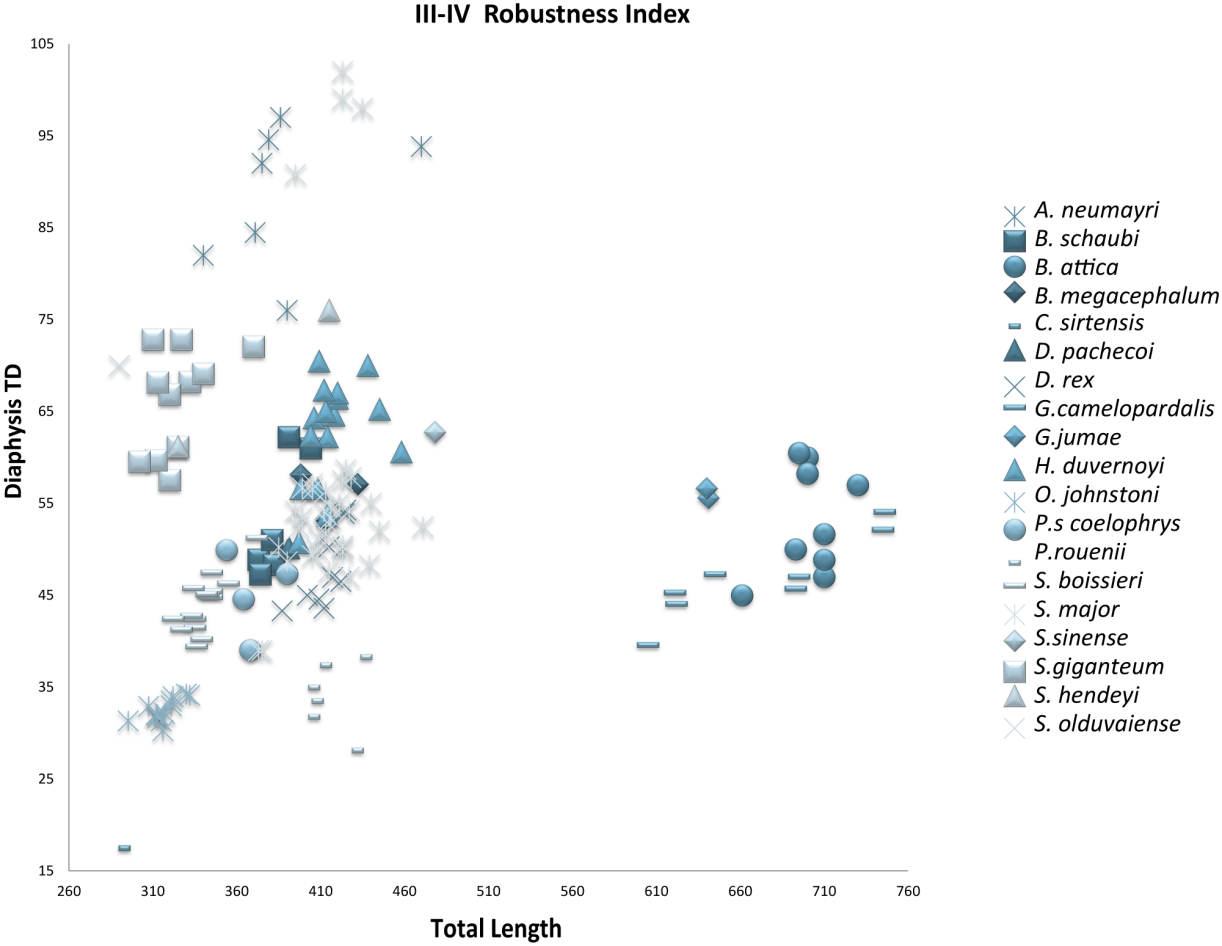
Robustness of the metacarpal III-IV of the giraffids analyzed. Metacarpal III-IV Robustness Index (RI) Diaphysis TD/Total Length*100. TD, transverse diameter. Measurements given in mm.

**Fig 37:**
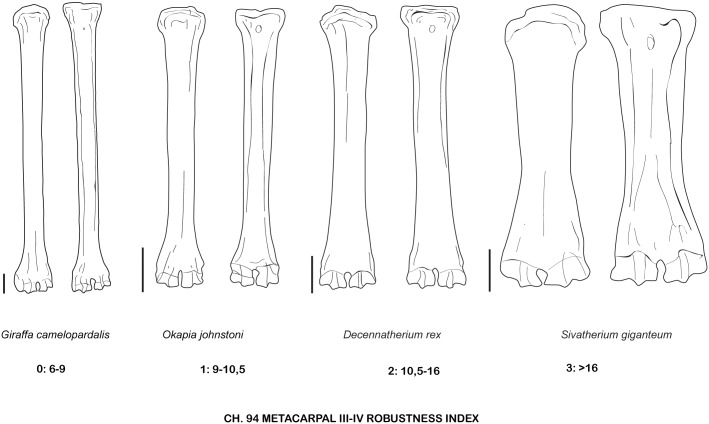
Character 94. Metacarpal III-IV Robustness Index (RI). RI = Diaphysis TD/Total Length*100. Scale bar equals 5 cm.

**Fig 38:**
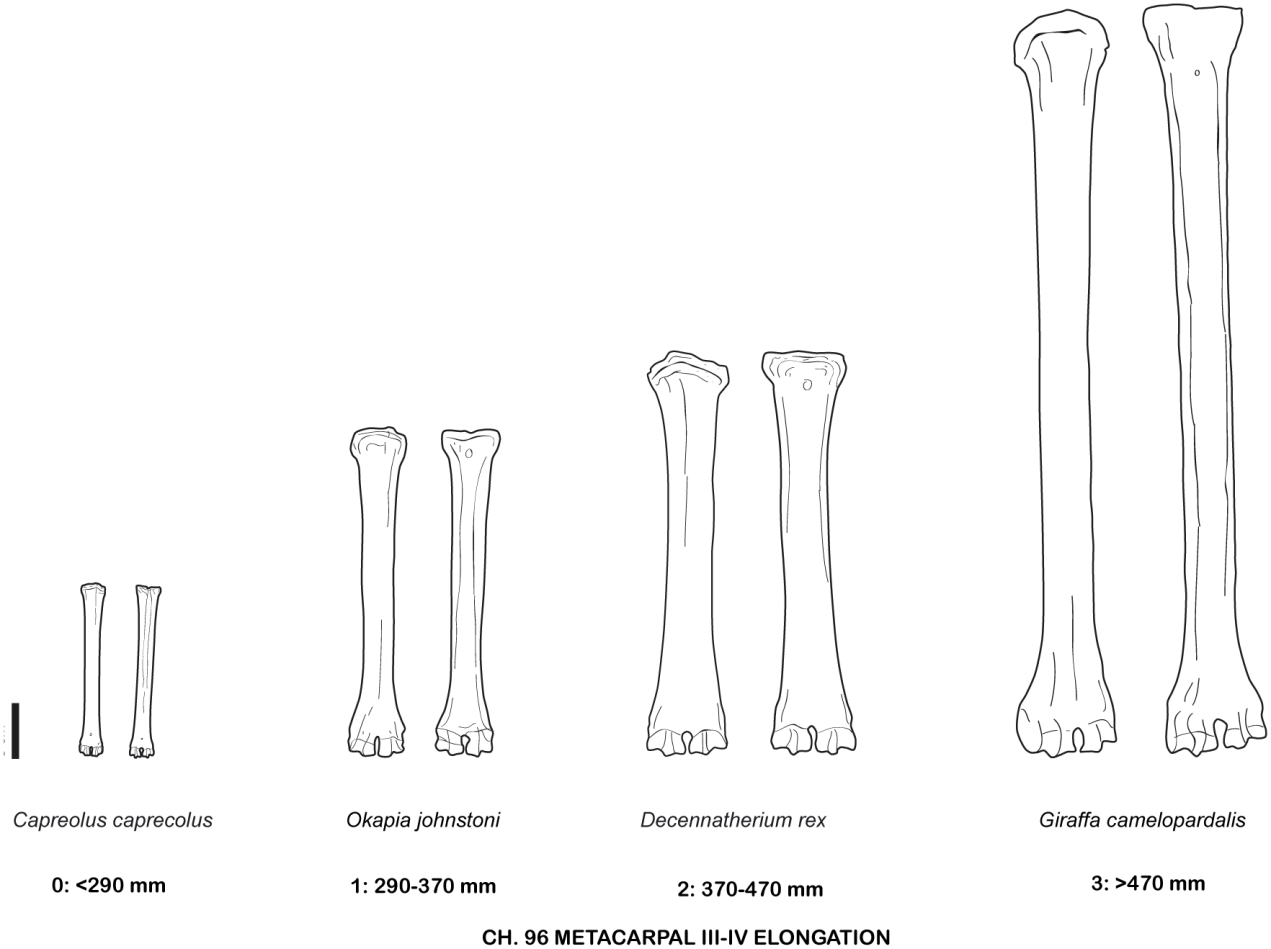
Character 96. Metacarpal III-IV elongation. Scale bar equals 5 cm.

**Fig 39:**
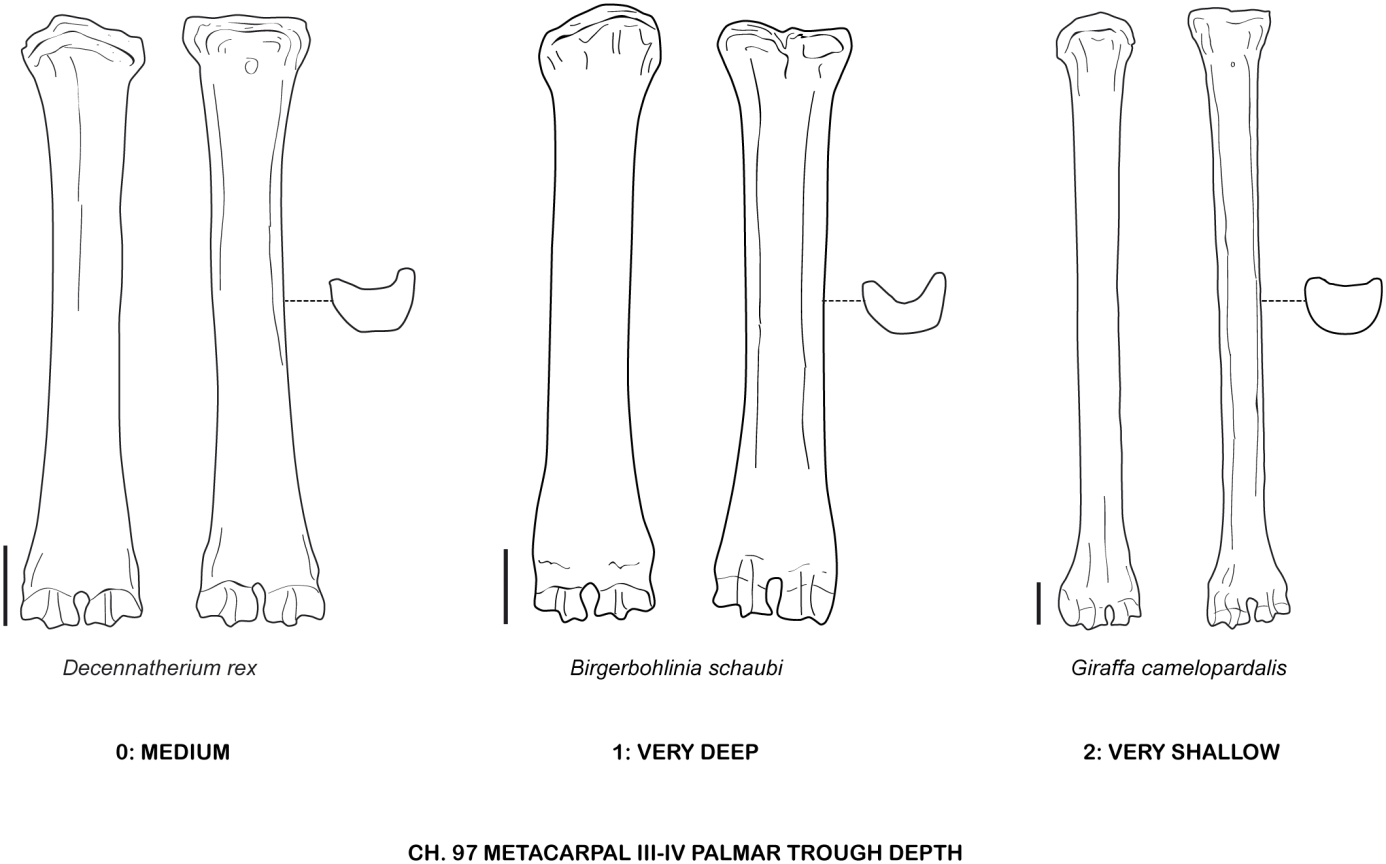
Character 97. Metacarpal III-IV palmar trough depth. Scale bar equals 5 cm.

**Fig 40:**
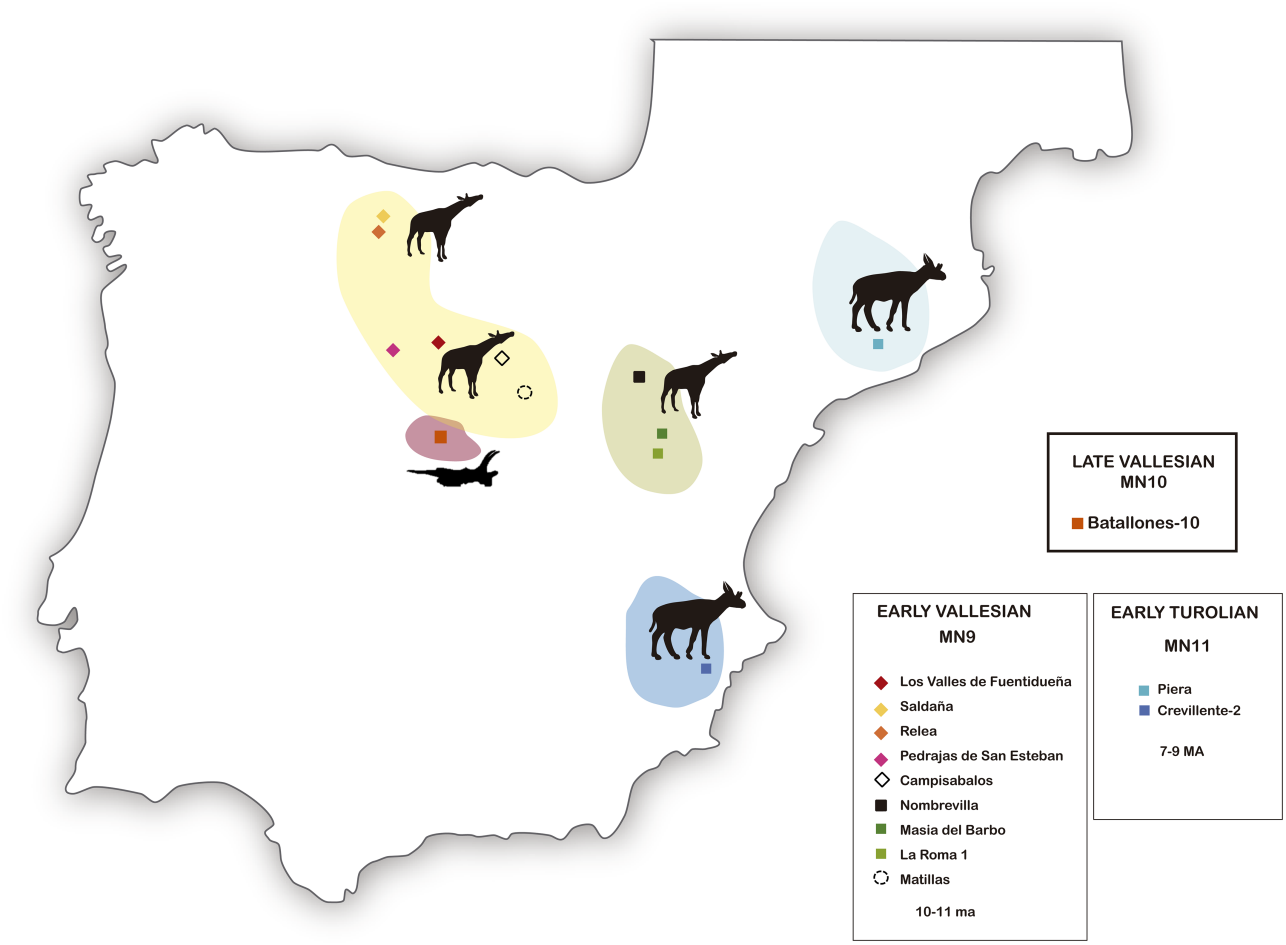
Distribution of the Iberian late Miocene giraffids.

**Fig 41:**
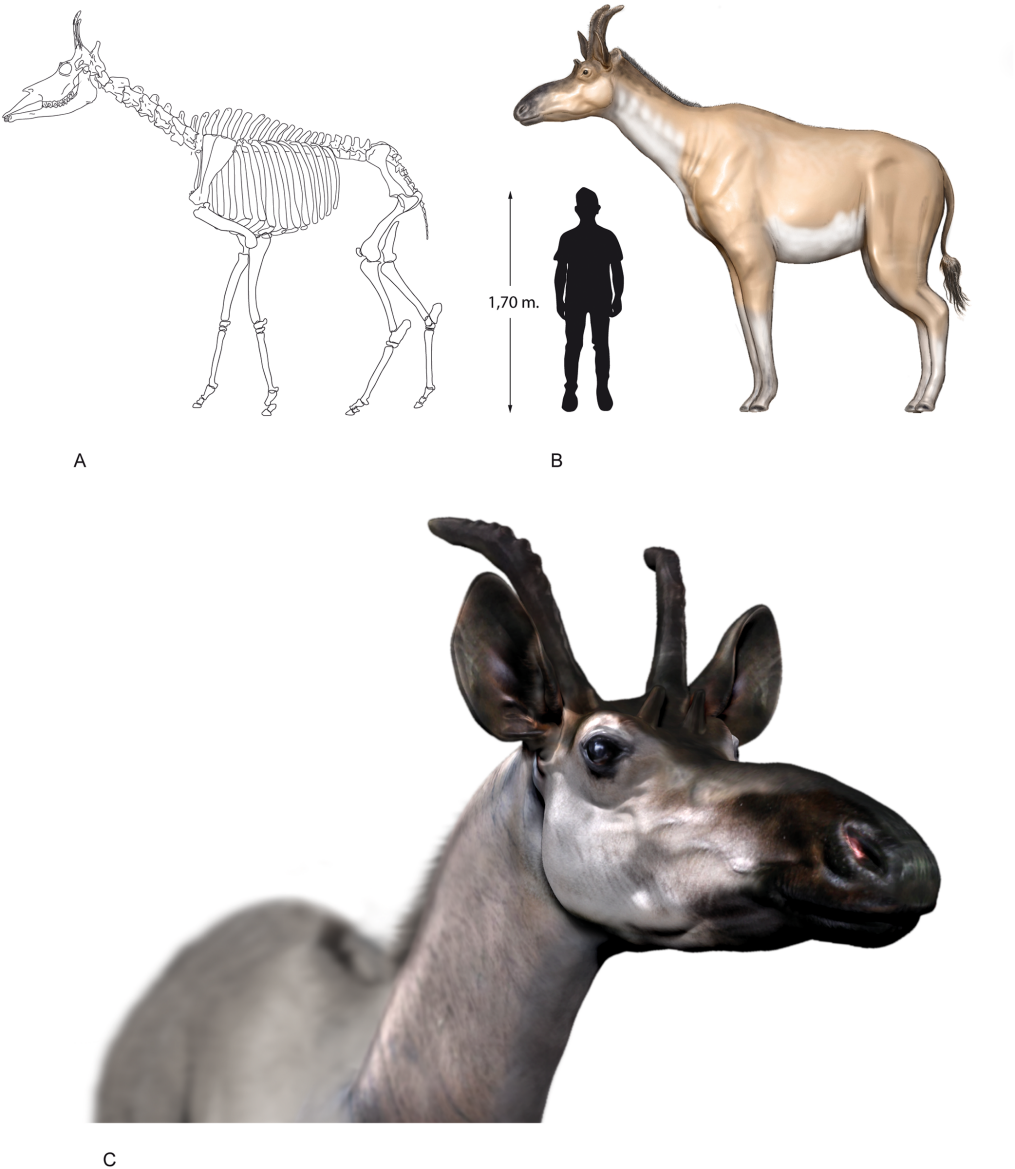
Reconstruction of *Decennatherium rex* sp. nov. from BAT10. A, skeletal reconstruction; B, life reconstruction of an adult female; C, life reconstruction of the head of an adult female. Illustration by Oscar Sanisidro.

LSID urn:lsid:zoobank.org:act:C5E9530A-6C05-4979-9DE7-FC9533CC22E4

#### Etymology

*Rex*, latin for king. Referring to the exceptional fossil remains of this species, that turn it into the king of the Miocene Iberian giraffids.

#### Diagnosis

A large giraffid with two pairs of ossicones. Smaller anterior pair located above the orbits and anteriorly oriented. Larger posterior pair located posterior to the orbits and posteriorly oriented. Posterior ossicones curved, showing a high number of ridges on their surface. Middle indentation of the hard palate slightly posterior to the M3. Broad occipital. Long premaxillae. Elongated diastema. p3 lacking mesolingual conid. Slightly molarized p4. Cervical vertebrae of medium length. Scapula with acromion. Robust postcranial skeleton. Metacarpals of medium length showing a medium depth palmar trough. Differs from *Decennatherium pachecoi* in: p3 with an isolated wall-like mesolingual conid structure; anterior stylid and conid of the p3 always present; and more robust metapodials.

#### Holotype

BAT10’13.E2-69, skull with ossicones and mandible (Fig 3).

#### Paratypes

The remaining referred material from BAT10.

#### Locality, age and horizon

BAT10, Cerro de los Batallones, late Vallesian, MN 10, local zone J, Madrid province, Spain. (ca 9 Ma) (Fig 1).

#### Material

BAT10’13.E2-69 (skull), BAT10’08.G3-91(skull), BAT10'09 F3-102 (skull), BAT10’07.G4-127 (calvaria with ossicones), BAT10'08 F1-112 (calvaria with ossicones), BAT10’13.D5-16 (right posterior ossicone), BAT10’13.D5-16 (left posterior ossicone), BAT10’09.F2-97 (left posterior ossicone), BAT10’07.G3-9 (right posterior ossicone), BAT10’10.F3-22 (posterior ossicone fragment), BAT10'07.S1 (posterior ossicone fragment), BAT10'08.F4-49 (right maxillar with P2-M3), BAT10’10.F3-64(right maxillar with P2-M3), BAT10’08.G1-33 (right maxillar with P2-M3), BAT10’13.D5-81(right maxillar with P2-M3), BAT10’08.F3-30(left maxillar with P2-M3), BAT10'07.H4-39 (left maxillar with P2-M3), BAT10’08.G1-45 (left maxillar with P2-M3), BAT10’13.E4-74 (left maxillar with P2-M3), BAT10'09.G2-177 (left maxillar with DP2-M2), BAT10’09.G2-123 (left maxillar with DP2-M2), BAT10'07 I4-1 (left hemimandible with p2-m3), BAT10'07 D5-44 (left hemimandible with p2-m3), BAT10'07.D5-22 (right hemimandible with p2-m3),BAT10'11.E3-21 (right hemimandible with p4-m3), BAT10'08.G2-51(left hemimandible with p2-m3), BAT10'10.F2-18 (left hemimandible with p2-m3), BAT10'08.G2-175 (left hemimandible with p2-m3), BAT10'08 G2-183 (left hemimandible with p2-m3), BAT10'13.E4-68 (left hemimandible with p2-m3), BAT10'11.C5-6 (left hemimandible with p2-m3), BAT10'13.E4-80 (left hemimandible with p2-m3), BAT10'13.D4-80 (right hemimandible with p2-m3), BAT10'07.G3-79 (left hemimandible with p2-m3), BAT10'11.D5-40 (left hemimandible with p2-m3),.BAT10'10.F5-9 (right hemimandible with p2-m3), BAT10'12.D6-85 (left hemimandible with p2-m3), BAT10'10.F2-18 (left hemimandible with p2-m3), BAT10 w/n (right hemimandible with p2-m3), BAT10'07.superficie (left hemimandible with p2-m3), BAT10'08.G2-167-168 (right hemimandible with p2-m3), BAT10’13.E2-69 (right and left hemimandibles with p2-m3), BAT10'09.F4-59 (right hemimandible with dp2-m2), BAT10'08.H4-13-14 (left hemimandible with dp2-m2), BAT10'09.F4-22 (left hemimandible with dp2-m1). The rest of the abundant cranial and postcranial material is listed in the S1 and S2 Tables.

## Description

### Cranial skeleton

The cranial remains (Fig 3) consist of four complete skulls and several maxillary fragments with part of the supraorbital area, the posterior appendage and part of the posterior skull attached, plus a number of unattached ossicones and hemimandibles.

#### Ossicones

The skulls have two pairs of ossicones (Figs 3 and 4). The smaller anterior pair is located above the orbits on the frontals. It is anteriorly oriented forming an angle with the skull roof of approximately 60°. Its surface has no ridges. The larger posterior pair is located posterior to the orbits over the parietals and is oriented posteriorly and slightly outwards, with and angle to the skull roof between 48–58°. The posterior ossicones are curved, ornamented by a high number of ridges on their surface, which run longitudinally from the base to the tip of the ossicone. The tips are blunt. We found detached complete ossicones and all show a hollow base. We find two distinct morphotypes depending on the size and development of the ossicones. Morphotype I (BAT10’08.G3-91, BAT10'09.F3-102, BAT10'08.F1-112, BAT10'07.S1) (Fig 4) shows a lower development of the ossicones, with the anterior ossicones reduced to unattached little circular disks with a pointy projection in the middle and slenderer posterior ossicones covered in ridges. They loosely resemble the bony spurs found in some *Hyemoschus* individuals [1], though in the case of the giraffids from Batallones these frontal ossicones are always present, in the same location and with the same structure and morphology. Posterior ossicones in morphotype I range from 225 mm to 270 mm All of the morphotype I ossicones that are attached to a skull are accompanied by adult dentition. Morphotype II (BAT10’13.E2-69, BAT10’07.G4-127, BAT10'16.D4-53BAT10’16.D4-20, BAT10´13.D5-16, BAT10'07.G3-9, BAT10’09.F2-97) (Figs 4 and 5) shows a higher development in both anterior and posterior ossicones. The distance between the bases of the anterior and posterior ossicones in morphotype II doubles that of morphotype I (~80mm vs ~40mm). The anterior ossicones reach 65 mm of length and the posterior 390 mm. The posterior ossicones are more robust and show very deep ridges on their surface. Some specimens have bumps, mainly located over the dorsal surface, although they can also appear laterally (Fig 6) (See S1 Table from measurements). The largest bump is always located on the middle of the dorsal surface, at a similar height on the right and left ossicones (observed in the same individual). This agrees with Solounias [80] who stated that this small irregular protuberances have a somewhat fixed position, suggesting a genetic basis. There is variability between individuals, with some specimens showing a larger number of bumps. According to Harris [81] the disparity in size and development of the bumps may represent individual variability, but it is also possible that the amount of secondary bone apposition, in the form of ornamentation, may be a function of the age of the individual. The surface of the ossicones is very porous, similarly to the surface of the ossicones of extant *Giraffa*. This may indicate that even with their large size they were covered in hair, irrigated by the cornual artery [82].

### Skull

In the BAT10 sample there are four skulls, two complete (one of each morphotype), and two almost complete (Figs 3 and 4).

In dorsal view the two supraorbital foramens are placed behind the anterior ossicones on the frontal bone. The occipital is wide and the superior occipital edge extends backwards and upwards, being much more prominent and extended than in *Giraffa* and *Okapia*. There is no development of the frontal sinus to hollow the ossicones. In lateral view the facial crest is deep. The lacrimal canal foramen is closed and the caudal lachrymal process is barely developed. The supraorbital canal orifices are rounded and wide. The orbit is rounded with the zygomatic process of the frontal bone very developed and rugose. The brow arch is very prominent above the orbit and the zygomatic arch is prominent under the orbit. The ethmoidal fissure is open and the temporal fossa is deep and subrectangular.The masseteric is large, with α values that range from 6,275° to 8,15° (n = 2) and β values that range from 81,03° to 109,37° with an average of 98,25°(n = 4) (Fig 7) [1]. The premaxilla is very elongated and straight and the nasal bone is prominent. The premaxillar shape is intermediate between the pointy browser shape and the squarer grazer shape [83]. The mandibular symphysis and lower incisors are horizontal in relation to the basipalatal axis. The maxillar tuberosity is very developed. In ventral view the foramen magum is rounded and deep and the intercondylar groove is wide. The jugular process is more developed than in *Okapia* and less than in *Giraffa*. The retroarticular process expansion is not fused to the bullae, which are not very developed, being similar to those of *Giraffa* rather than the large bullae of *Okapia*. The position of the sheath of the eardrum varies little relative to the bullae. The auditory meatus is rounded and oriented laterally. The basioccipital muscular tubercules of the occipital are very prominent and rounded and are much more developed than in *Giraffa* and *Okapia*. The foramen ovale are rounded and deep, similar to those on *Okapia* rather than the more subtles ones of *Giraffa*. The palatine choane do not expand as anteriorly as in *Giraffa*, reaching only the posterior part of the M3, similarly to *Okapia*. The grand palate foramina are located next to the M3 while in *Okapia* and *Giraffa* are next to the M1. The mastoid is reduced.

The skulls were extensively measured (S1 Table) and show variability, both metrical and morphological. These differences appear to be related mainly to the difference in preservation states, as most suffer some degree of dorsoventral and mediolateral compression (e.g. BAT10’08.G3-91, BAT10’13.E2-69), mostly on the frontal and facial area. There is also variability that actually reflects the intraspecific differences between the two morphotypes found in the Batallones sample as well as the ontogenetic development of this giraffid (e.g. S1 Text. Measurement 20: skull with at the ossicone basis).

### Mandible

Most of the hemimandibles (n = 25) (Fig 8) correspond to adult individuals, with only 12% of the sample being juveniles in different states of development. One specimen corresponds to a specially young individual (according to [62] in extant giraffes BAT10’09.F4-22 would not be older than 4 months). In dorsal view the coronoid process is well developed and its uppermost part is laterally oriented. The condylar process is oval and extends medially (see Fig 4D). Both the mandibular and the mentonian foramina are large. The mandibular foramen appears in the caudoventral side of a very shallow pterygoid fossa. The masseteric fossa is slightly ventrocaudally expanded and its surface is slightly rugous except at the vascular incision where there is a more rugous and bulgy area. This configuration indicates that the deepest part of the masseteric muscle is more developed than the superficial part [84]. The diastema is very elongated. There are slight variations in the ramus width with some thinner specimens (BAT10 w/n, BAT10'10,F5-9) and others wider than the average (BAT10'13.D4-80). Medially the surface for the medial pterygoid muscle is rough.

### Hyoid

In ventral view the skull BAT10’13.E2-69 shows a portion of the hyoid bone (Fig 3C), particularly a very elongated stylohyoideum portion. A slight curved portion of the epihyoideum can also be seen.

### Dentition

#### Upper dentition

The DP2 (Fig 9A) is longer than wide, and shows a strong buccal cone. The only two recovered belong to the same individual and are highly worn out (Fig 9A). The DP3 (Fig 9A) is bi-lobed, slightly molarized, and also longer than wide. The anterior lobe is longer and the posterior lobe is shorter and wider. It has a highly developed mesostyle and the parastyle is weak. There is a lingual cingulum. The internal wall is complete. The DP4 (Fig 9A) is molariform with strong paracone and metacone. The meso and parastyles are equally well developed. Both the protocone and metaconule have a rounded lingual border.

The P2 (Figs 3, 4 and 8A) is longer than wide. The buccal cone is strong and closer to the anterior style. The anterior style is more developed than the posterior style, which is barely developed in most specimens. However, this is not the case of BAT10’10.F3-64 where the posterior style is very developed and projects anterobuccally. The anterior style is straight and also very strong, but less than the anterior style. The P3 (Figs 3, 4 and 8A) is longer than wider. It has a strong and pointy buccal cone, closer to the anterior style, and a moderate anterior style. In most specimens the posterior style is barely developed except in BAT10’10.F3-64 where the posterior style is thicker than the anterior style. It is shorter in height than the anterior style and projects anterobuccally. On the posterior part of the medial notch there is a spur protruding from the posterobuccal crista to the posterolingual crista that shows different degrees of wearing. In the most worn out specimens it connects the two posterior cristas (BAT10’10.F3-64, BAT10’08.G1-45). The P4 (Figs 3, 4 and 8A) is wider than long. The buccal cone is moderate and closer to the anterior style. The anterior style is moderate, thicker than the posterior style. The lingual face is simple, with a half-moon shape and in some specimens there is a weak posterolingual spur and sometimes also a weak posterobuccal spur (BAT10’13.E4-74). The M1 (Figs 3, 4 and 8A) has a buccal wall with stronger parastyle and mesostyle and a much weaker metastyle. The paracone is very strong, and the protocone and metaconule are lingually V-shaped and are well separated, with the posterior lobe slightly wider than the anterior one. The enamel is very rugous. There is metaconule-fold. The M2 (Figs 3, 4 and 8A) is similar to the M1, but larger. The M3 (Figs 3, 4 and 8A) has a hypsodoncy index (height/length in an unworn specimen) between 0,78–0,80 (N = 3). It is very similar to M1 and M2 but it shows a very pointy metaconule (BAT10’13.E4-74) and the metacone in unworn specimens is rounder than the paracone (BAT10’08.F4-49). Also buccally there is a small basal pillar next to the mesostyle in two of the specimens (BAT10’13.E4-74, BAT10’10.F3-64). Finally in BAT10’13.E4-74 there is a spur at the pre-protocrista, similar to the metaconule fold. The length/width index (L/W) mean values of the upper molars are 1.12 (n = 34), similar to those of most of giraffids (~ 1.024, n = 810).

#### Lower dentition

The bi-lobed lower canine (Fig 10) has a large rounded anterior lobe larger than the posterior one. The larger anterior lobe is rounded and has a thin depression along the midline. The smaller lobe is more pointed and has a posterior small crest that slightly projects buccally. The deciduous canine (Fig 10) has the anterior lobe only slightly larger than the second lobe. The anterior lobe is rounded, whereas in *Giraffa camelopardalis* and *Okapia johnstoni* is pointed. The posterior lobe is pointed and triangular in shape. The incisors (Fig 10) are wide and have a straight crown, and are morphologically similar to those of *Giraffa camelopardalis* and *Okapia johnstoni*. The deciduous incisors are smaller versions of the adult ones.

The dp2 (Fig 9B) is a simple tooth with no morphological variability. It is smaller than the p2 but morphologically very similar to it. It has a strong mesobuccal conid, separated from the posterolingual and posterobuccal conids. The dp3 (Fig 9B) is elongated, with a larger anterior lobe and a smaller posterior lobe. The anterior stylid is present. The mesobuccal conid is strong and coniform. The posterolingual conid joins the posterobuccal conid, creating a posterior notch. There is an antero-buccal cingulid. The dp4 (Fig 9B) has three lobes, being the most anterior one smaller than the posterior. There is small basal pillar between each lobe. The lingual wall has a moderately developed metastylid. The similarly sized metaconid and entoconid are aligned. The buccal wall has a well-developed protoconid and hypoconid.

The p2 (Fig 8B–8D) is a simple tooth with no morphological variability (N = 21). The anterior conid is strong and there is no anterior stylid. The strong mesobuccal conid is separated from the posterolingual and posterobuccal conids. The p3 (Fig 8B–8D) always has an anterior stylid. The posterolingual and posterobuccal conids are separated. They run parallel in the buccal side and connect lingually. The mesolingual conid is absent and the mesobuccal conid is strong and coniform. There are four distinct p3 morphotypes in *D*. *rex*: morphotype I has a slightly bifurcated posterolingual conid (morphotype I, 47,61% n = 10, N = 21); morphotype II shows a simple posterolingual conid (morphotype II, 33,33% n = 7, N = 21); morphotype III has a posterior lingual conid just next to the posterolingual conid but more lingually positioned and about the same height (morphotype III, 14,28%, n = 3, N = 21); finally morphotype IV has an almost complete lingual wall (morphotype IV, 4,76%, n = 1, N = 21). Most specimens belong to morphotypes I and II. Fig 11 shows these morphotypes as well as the occlusal views of the p3 of the rest of the giraffids in this work (Fig 11N). The p4 (Fig 8B–8D) is molarized, with a large anterior lobe that represents two thirds of the tooth length. The mesobuccal and mesolingual conids are strong and coniform. The posterolingual conid is diagonal or aligned to the mesolingual conid, and is attached to the posterobuccal conid. The posterobuccal conid is slightly bifurcated. There is a strong incision on the buccomedial wall between the posterobuccal and mesobuccal conids. There is a small medial valley. In the anterior side of the lingual wall there is a fold that starts on the anterior side of the preprotocristid and reaches the lower part of the lingual wall at the metaconid level. The second lobe is expanded (posterior length/ total length*100) with an average of 36,62% (N = 16) [10].

The lower molars (Fig 8B–8D) have a lingual wall with a moderate metastylid. The metaconid and entoconid are aligned and similar in size. The buccal wall has a well-developed protoconid and hypoconid. In the anterior side of the lingual wall there is fold that starts on the anterior side of the preprotocristid and reaches the lower part of the lingual wall at metaconid level. There is a buccal basal pillar between the anterior and posterior lobes with a variable degree of development, but always larger in the m1. The m3 has a large third lobe, formed by a strong semicircular hypoconulid in lingual position. The semicircular hypoconulid is attached to the posterior side of the hypoconid and entoconid. In most specimens there is a lingual pillar between the hypoconulid and the entoconid, next to the postentocristid and the posterior notch (e.g. BAT10'10.F5-9), although it is not present in others (e.g. BAT10'12.D6-85).

### Axial skeleton

#### Atlas

The atlas (Fig 12) has a cranio-centrally positioned and poorly developed dorsal tubercle on the dorsal arch. The cranial margin of the dorsal and ventral arches is C-shaped. The interarticular indentation is thin. The margin of the dorsal arch terminates on the same plane as the margin of the ventral arch. In lateral view, the alar wing is thin and slightly sigmoid shaped. The ventral tubercle is a posteriorly positioned triangular bulge that protrudes caudally.

#### Axis

The spinous process is well-developed, often massive and triangular in shape (Fig 13). In dorsal view, the spinous process is caudally thicker than cranially, slightly arched and inclined cranially. The cranial edge of the spinous process is caudally positioned to the cranial edge of the dens. The dens is dorsally concave and has a rounded cranial end. In its most cranial part there are two rounded lateral concave depressions separated by a bony bulge. The cranial opening of the foramen transversarium is very wide. The caudal articular facets are oval shaped and protrude laterally. The transverse process protrudes laterally and the caudal indentation is semicircular. The ventral ridge is thin and continuous throughout the length of the vertebral body, thickening caudally and protruding more caudally than the caudal vertebral fossa.

#### Cervical vertebrae

In dorsal view, the spinous process is situated between the base of the cranial and caudal articular processes, in the middle of the dorsal vertebral body (Fig 14). It is elongated and bulgy and is directed dorsally. The spinous process extends by the majority of the length of the dorsal vertebral body. One distinct ridge radiates from the caudal aspect of the spinous process onto the dorsal lamina. The cranial articular facets are oriented dorsomedially and are rounded. The cranial bulge is convex and oval shaped. The tip of the cranial bulge protrudes over the cranial articular processes. The cranial articular process and facet extend cranially to the same level as the tip of the cranial bulge. In lateral view, the ventral tubercle of the transverse process extends cranially, extending further than the cranial bulge tip. The dorsal tubercle of the transverse process forms a wing that protrudes laterally and connects to the caudal part of the ventral tubercle of the transverse process. The caudal aspect of the vertebra is about the same size of the cranial part. The caudal articular facets are rounded. In ventral view, the cranial end of the vertebra is narrower than the caudal end. The ventral ridge is prominent and continuous longitudinally on the vertebral body. C3s are slightly shorter than the axis and more elongated than the rest of the cervical vertebrae (C4 to C7) that gradually become shorter and wider, while the spinous processes become shorter and higher.

#### Thoracic vertebrae

In cranial view, the cranial bulge is triangular (Fig 15). The cranial articular processes are oval shaped and are located slightly anteriorly than the tip of the cranial bulge. In dorsal view, the spinous process is located posterior to the cranial articular processes and is oriented dorsocaudally. It is thicker at the base, becoming thinner towards the top. The spinous processes width is constant along their length. Their height increases suddenly from T1 to T2, reaches a maximum at T2—T4, and then decreases like in the giraffe [85]. The first spinous processes are almost vertical, the T4—T7 segment presents a slight caudoversion, which disappears at the last vertebrae, which are vertical[85]. The transverse processes are thick and protrude laterally and are located exterior to the cranial articular processes. The caudal articular processes are oval and oriented ventrocaudally. In ventral view the ventral crest of the body is cranially bulgy. There is variability in the orientation of the caudal articular processes. In BAT10'08.D5-10 they are ventro-caudally oriented while in BAT10’07.superficie they are completely ventrally oriented. This also indicates a change in the angle of the spinous process, more vertical in BAT10'08.D5-10 and more caudal in BAT10’07.superficie. This is probably due to the more anterior position of BAT10'08.D5-10 and hence the inclination of the process augments proportionally with the relative position of each thoracic vertebra. This is also reflected in the orientation of the cranial articular processes, that is more craniodorsally-oriented in BAT10'08.D5-10 and more dorsally-oriented in BAT10’07.superficie.

#### Lumbar vertebrae

The cranial bulge is heart-shaped (Fig 16). The spinous process is located posterior to the cranial articular processes and the mammillary processes. The shaft of the spinous process is dorsally oriented and shows a slightly rugged surface towards the upper part indicating the insertion for the multifidus muscle. The top part of the spinous process projects cranially. It is bulgy, thick and rugous indicating a strong insertion for the wide dorsal muscle and the longissimus and spinous lumbar muscles. The medially oriented cranial articular processes are rounded and curved, and protrude further than the tip of the cranial bulge. The mammillary process is bulgy and has a very rugous surface indicating a strong insertion for the multifidus, longissimus and spinous muscles. The transverse processes are thin and protrude craniolaterally showing in some cases a sigmoidal curvature. They are located exterior to the cranial articular processes. The caudal articular processes are rounded. Some caudal articular processes show half of the circular facet oriented ventrolaterally and the other half oriented cranially (e.g.BAT10'13.D4-40, BAT10. SN). Other specimens show half of the circular facet oriented ventrolaterally and the other half oriented laterally, with a concave surface (BAT10'08.G4-119, BAT10'09.G5-25). In ventral view the ventral crest of the body bulges cranially and the surface of the vertebral body is rugous showing a strong insertion for the diaphragm and the psoas minor muscle. Regarding variability, the L3 BAT10’14.E1-2 shows a spinous process with a curved posterior outline for ¾ of the shaft and a slightly longer spinous process than the rest of the lumbars. Compared to the L3 the L4 BAT10’14.E1-3 has a straighter outline of the posterior spinous process, and the L5 BAT10’14.E1-4 shows a stronger and wider dorsal part of the spinous process. Also its spinous process is wider and a shorter than those of the rest of the lumbars. The transverse process is longer and distally thinner than the transverse process of the other lumbars.

#### Caudal vertebrae

We recovered eleven coccygeal vertebrae from the same individual (Fig 17) (BAT10’14.E2-28, BAT10’14.E2-29, BAT10’14.E2-30, BAT10’14.E2-31, BAT10’14.E2-39, BAT10’14.E2-40, BAT10’14.E2-47, BAT10’14.E2-48, BAT10’14.E2-s/n a, BAT10’14.E2-s/n b, BAT10’14.E2-s/n). The first three coccygeal vertebrae are more robust and larger (BAT10’14.E2-28, BAT10’14.E2-29, BAT10’14.E2-30). The vertebral head is pentagonal and the vertebral body is elongated. The hemal process is only slightly developed. The mammillary processes are thin and latero-ventrally oriented. The transverse processes are thin and protrude laterally. There is a gradual size reduction in the absolute length of the vertebral body from the first to the last coccygeal vertebrae. The transverse processes also get reduced, totally disappearing at the fourth vertebra. The mammillary processes also get smaller, disappearing in the last three vertebrae.

#### Ribs

The tubercle border has a tear-drop shape. The neck is short and concave. The head has two rounded facets on the medial and lateral sides. There is a tuberosity for the longissimus muscle. On the cranial border there is a rugous surface for the iliocostal muscle. The costocondral articulation is wider than the ventral extremity of the rib.

### Appendicular skeleton

#### Scapula

In lateral view the glenoid cavity is rounded with a thick border (Fig 18). The supraglenoid tubercle is very developed and rugous indicating strong biceps brachii muscle as well as supraspinous muscle. The acromion is developed and rugous. The scapular spine is developed and rugous, disappearing towards the end of the scapula, indicating a strong trapezius and deltoid muscles. The infraspinous fossa and the supraspinous fossa are triangular. The infraspinous fossa has a strong rugous border, especially on the caudal angle indicating strong insertions for the triceps brachii long head muscles.

#### Humerus

The apex of the tuberculus major is caudally massive and curves medially indicating a strong supraspinatous muscle (Fig 19A–19F). The tuberculus major is large and very developed with a very rugged surface for the insertion of the deep infraspinatous muscle. The tuberosity for the teres minor is also developed and has a rugous surface. The deltoid tuberosity for the deltoid muscle is moderately developed. The apex of the tuberculus minor is developed and rugous, indicating the insertion for the subscapularis muscle. The convexity of the minor tubercle is semicircular. The humeral head is rounded and has a smooth surface. The area below it is slightly rugous, indicating the insertion of the brachialis muscle. The insertion for the teres major is present as a depression on the medial side. The olecranon fossa is triangular and deep. The lateral and medial epicondyles have very rugous areas caudally and there are also some small tuberosities on the lateral side of the lateral epicondyle for the insertion of both the extensor digitorum communis muscle and the extensor carpi radialis muscle. On cranial view there is a very rugous area right above the capitulum indicating a very strong extensor indicis musle and the extensor digitorum communis. There is also a bulgy rugous area in the diaphysis for the pectoralis descendens muscle.

#### Radius / Ulna

In dorsal view the dorsoproximal coronoid process is pointed (Fig 19G–19L). The proximal facets for the ulna are well preserved. The lateral one, smooth and semicircular, is located in a moderately developed pit. The smaller and triangular medial facet does not touch the lateral one. In proximal view, the medial half of the humeral articulation facet is dorsopalmarly and lateromedially wider than the lateral half. The lateral border of the capitulum facet is more or less straight. The groove for the lateral lip of the distal trochlea of the humerus is wide and moderately deep. There is a soft step between the capitulum facet and the rest of the proximal joint. The lateral tuberosity for the insertion of both the flexor digitorum superficialis muscle and the lateral extensor digitorum communis muscle is highly developed. The dorsal groove for the extensor carpi radialis muscle is wide and very shallow, curving towards its distal part. The crests that limit the dorsal groove show a similar development. On the lateral surface the groove for the extensor digitorum lateralis muscle is clearly marked but shallow. Distally, the scaphoid facet is palmarly convex and dorsally concave, and almost oval in shape. Its dorsal contour is more irregular than that of the semilunate facet, which is completely semicircular. The semilunate facet is palmarly convex but flattens dorsally, and its lateral and medial crests reach the palmar side of the radius forming a groove. The triquetrum facet is flatter and its dorsal border is semicircular. The dorsal border of the olecranon is convex and the palmar border is slightly concave. The olecranon tuberosity is strong. The styloid process is well developed.

#### Scaphoid

In medial view the dorsal border is straight (Fig 20A–20F). The proximo-palmar process is wide and blunt and it is placed almost at the same level than the dorsal process. The palmar outline shows a proximal bulge. On the disto-palmar area there is a small projection with no articulation facet that in bovids and cervids sports another facet for the semilunate. The proximal side is wide, with a sinuous surface, being dorsally convex and turning palmarly concave. The distal articulation for the magnotrapezoid is wide.

#### Semilunate

The proximal facet is concave, with dorsal and palmar bulges (Fig 20G–20L). The dorsal facet is dorsally wider than the distal facet, while the distal facet is palmarly wider than the proximal facet. The proximomedial facet for the scaphoid is complete and straight while the distomedial facet is rectangular and is only dorsally present. In lateral view the dorsoproximal facet for the triquetrum is triangular and wide, and the distopalmar facet for the triquetrum is semicircular. The distal facet for the hamatum is narrower than the magnotrapezoid facet. The keel between these two facets is blunt and the posteropalmar area shows an acute concavity.

#### Triquetrum (Os carpi ulnare)

In lateral view the dorsal profile is slightly concave (Fig 20M–20R). The distopalmar process is thick and well developed. The profile of the distal facet is concave. The pisiform facet is subrectangular with soft rounded corners (almost oval). In dorsal view, the proximal facet for the semilunate is developed and rounded. On the medial surface the central facet is large and subtriangular. The well-developed central knob placed behind this facet is slightly connected to it. The internal facet of the distal process is semicircular in shape.

#### Pisiform

In lateromedial view the pisiform has an asymmetric contour (Fig 20S–20W). The dorsal border is rounded and convex, and the palmar area is thick. The dorsal facet for the triquetrum is pear- shaped and transversely thick.

#### Magnotrapezoid

In proximal view the magnotrapezoid has an almost square shape (Fig 21A–21F). The edge between the proximal facets for the scaphoid and the semilunate shows an inflection of ~130°. The lateral facet for the semilunate is narrower than the facet for the scaphoid. The outline of the palmar border is nearly straight. The wide facet for the hamatum reaches the distal surface, extending proximally towards its palmar side. There is a small groove for ligament attachment in the distopalmar end of the medial surface. In palmar view the border for the scaphoid facet shows a medial elongation towards the distal face.

#### Hamatum

The proximal surface is semicircular and shows two distinct facets (Fig 21G–21L). The proximal facet for the triquetrum is broad, lateromedially concave and wider than the facet for the semilunate. Medially there is only one scythe-shaped continuous facet for the magnotrapezoid, that extends palmarly. The lateropalmar process is well developed, slightly surpassing the distal edge of the distal facet.

#### Metacarpal III-IV

The metacarpals are relatively robust compared to *Decennatherium pachecoi*, with a robustness index (RI) that ranges from 10,58 to 12,76 [86]. The proximal articular surface is semicircular, with the sub-quadrangular facet for the magnotrapezoid occupying more than 2/3 of the available area (Fig 22). The hamatum facet is triangular in shape. The keel between both facets is well marked and thin. Both the hamatum facet and most of the lateral part of the magnotrapezoid facet extend palmarly. The oval synovial fossa is well developed and closed. The diaphysis widens distally. The central groove on the diaphysis dorsal surface is very shallow, almost imperceptible. The dorso-proximal tuberosity where the tendon of the extensor carpi radialis muscle is inserted is well developed. In lateral position the sulcus for the lateral extensor tendon is short. On the proximal end of the palmar surface there is a rugous area where the interosseous muscles and the flexor carpi radialis muscle insert. The proximal areas of the palmar ridges are rugous. There is a central palmar concavity on the diaphysis called posterior trough [1]. This trough is shallow, extending approximately two-thirds down the shaft length. The distal joints are proportionally short and wide, and the keels are dorsally poorly developed, turning slightly sharper palmarly. The inter-trochlear incision is wide but its shape is not discernible due to the presence of sediment between the two trochleas. In lateral view the dorsal part of the distal articular surface is rounded (not flattened). The internal articular surfaces of the pulleys are wider and more rectangular than the external ones. In the specimen BAT10’09.G2-81 there is a noticeable difference between the two distal trochleas. The lateral one extends further distally than the medial trochlea, placing the distal articulation surface at two different levels, the lateral lower and the medial higher. This disposition may be interpreted as an osteological pathology, possibly being caused by a fracture during this individual’s life.

#### Pelvis

The ischiatic tuberosity is very developed and bulgy, especially in the attachment area for the semitendinous muscles (Fig 23). The region of attachment for the gluteo-bideps/femoral biceps mucle is rugous. The ischium body is wide and the sciatic spine is not very developed. The branch of the ischium is thin but has a strong pubian spine for the pectine muscle. The obturator foramen is rounded. The acetabulum is rounded and deep, with a thick border. The acetabulum fossa is rounded and the acetabular incision is very thin. The semi-lunate fossa of the acetabulum is soft and C-shaped. The insertion surface for the right muscle of the cuisse is rugous. The ilium neck is wide but short. The ilium is large and highly expanded with a developed dorsocranial spine and an even larger ventrocranial spine.

#### Femur

Proximally, the apex of the great trochanter is moderately developed, with a rugous surface for the gluteus medium muscle (Fig 24A–24F). The crest of the great trochanter, where the broad lateral muscle inserts, is very developed and highly rugous. The femur head is rounded. The neck of the femur is concave and rugous. The small trochanter is also very developed and rugous, showing a strong insertion for the broad medial muscle. Caudally, the intertrochanteric crest is very thick and strong, and the trochanteric fossa is oval and deep. The medial and lateral condyles are asymmetrical, being the lateral condyle larger than the medial. The intercondylar fossa is deep. Medially, the trochlear tubercle and the medial lip of the trochlea are large and bulgy. Medially, the medial epicondyle has a rugous surface. There is a rugous tuberosity for the gastrocnemius and semimembranosus muscles above the medial epicondyle. There are two lateral fossetes, one for the popliteal muscle and the larger more ventrally positioned for ligament insertion.

#### Patella

The patella is triangular and cranially broad (Fig 24G–24L). On the lateral side it has a crest for the fascia lata and a very rugous and bulgy area for the insertion of both the gluteofemoral muscle and the broad lateral and broad intermediary muscles. On its dorsal side there is a rugous area for the articular knee muscle. Caudally, the articular surface for the femur is subquadrangular in shape.

#### Tibia

In dorsal view the tibial tuberosity is very developed and rugous in the insertion area for the gluteo-biceps muscle (biceps femoral and fascia lata), and the semi-tendinous muscle (Fig 24M–24R). The intercondylar eminences are well developed. The lateral condyle has an almost straight lateral profile, curving plantarly. The medial condyle is almost semicircular, with a rounded medial outline and a rugous area indicating the insertion of the semi-membranous muscle. The popliteal fossa forms an acute concavity. The diaphysis is robust, with a flat dorso-distal surface that confers a quadrangular shape to the cross section of this area. Dorsally the tibia lacks a medio-distal crest. The distal epiphysis is better preserved. In dorsal view the central protuberance is relatively wide and well developed. The dorsal border of the protuberance is straight and placed at a lower level than the medial malleolus. In dorsal view the facet for the fibula is located on the lateral side of the tibia distal border. The medial malleolus is strong, narrowing distally. On the plantar side the groove for the tendon of the flexor digitorum longus muscle and the tibialis caudalis muscle is wide and shallow. The distal plantar surface is moderately convex. In distal view the astragalus articulation (distal cochlea) shows an asymmetric contour, being more developed medially than laterally. The medial half of the distal cochlea is narrower and deeper than the lateral half. The plantar facet for the fibula is concave and bigger than the dorsal facet.

#### Fibula

The outline in lateral view is rectangular or subtrapezoidal, with the dorsal border flatter than the plantar border (Fig 24S–24X). The proximal spine is strong. It is slightly displaced plantarly, and well separated from the dorso-proximal angle. The dorso-proximal angle is located at the same high or slightly below the proximo-plantar angle.

#### Calcaneum

The dorsal outline of the calcaneum body is straight or slightly convex (Fig 25A–25F). The plantar border is somewhat concave, widening and forming a bump just above the distal end. The plantar surface of the calcaneum body is thick and rough. The proximal and lateral prominences for tendinous insertion of the tuber calcanei are strong. In dorsal view they can reach the same height or the lateral prominence may be more developed than the lateral one. In proximal view the tuber calcanei contour is typically heart-shaped clearly showing the plantar groove and the transversal rim of the insertion area for the gastrocnemius muscle. The sustentaculum tali has a convex proximal border and a rounded and concave distal border. In the distal area of the lateral surface there is a very thickened and bulky area that occupies most of the dorsal part. Farther back of this protuberance a groove-like depressed area can be appreciated. The prominent fibula facet is divided into a larger proximal convex facet and a distal one shorter and slightly concave. The distal facet for the astragalus is long and its dorsal border is inclined.

#### Astragalus

In dorsal view the proximal trochlea is longer than the distal one, which is typical of giraffids and can be appreciated in *Giraffa camelopardalis* and *Okapia johnstoni*. (Fig 25G–25L). Their medial and lateral borders are straight and parallel, aligned with the borders of the distal trochlea. The lateral lip of the proximal trochlea is thicker than the medial lip. The gorge of the proximal trochlea is wide and the central fossa is very deep. The central projection of the medial border is well developed and blunt. The gorge of the distal trochlea is shallow. The medial lip of the trochlea is shorter than the lateral one and has a more rounded distal border. The proximoplantar prominence is thick and more or less projected in all the specimens. The fossa located in the distal area of the medial surface is relatively deep. In lateral view the central surface of the bone is convex, with an ample fossa on the distal side that has a rough surface. The distal facet for the calcaneus is centered or only slightly displaced relative to the proximodistal edge of the astragalus, and shows a semicircular or kidney-shaped profile. The plantar facet for the calcaneus has a small central concavity, and its medial border is slightly curved.

#### Navicular-cuboid

The proximal articulation for the astragalus is subrectangular (Fig 25M–25R), with a shallow plantar fossa between its two halfs. The lateral facet for the calcaneus is long and wide, almost reaching the dorsal border of the bone. In medial view the plantar border is irregular. There is a distal step between the dorsal facet for the ectomesocuneiform and the facet for the entocuneiform. In lateral view there is a distoplantar tuberosity and an insertion groove for the peroneus longus muscle that is moderately wide and well-marked in all the specimens. The laterodistal tuberosity is moderately developed. The dorsal facet for the metatarsal is oval and has a small medial inbound. The medial facet for the ectomesocuneiform is kidney-shaped. The facet for the entocuneiform is convex an oval. On the plantar side there is well-developed proximo-plantodistal process. On this process there is an inclined crest that that extends through all the plantar surface. Lateral to this crest there is a well-developed semicircular canal.

#### Ectomesocuneiform

All the ectomesocuneiforms are similar in size, proportions and morphology, and resemble those of extant giraffids. However, in *Okapia* all the cuneiforms are fused to the navicular-cuboids, and in *Giraffa* the ectomesocuneiforms and the entocuneiforms are also fused forming a single piece. In proximal view the general contour of the ectomesocuneiform is sub-elliptical (Fig 26A–26F). The surface of the proximal articulation is concave. In the lateral surface the dorso-proximal facet is elongated, semicircular and variable, being bigger in BAT10’12.D5-53 and BAT10’09.F2-40 and smaller in BAT10’12.D6-130. The distal facet for the metatarsal is kidney-shaped, slightly dorsally convex and plantarly flat.

#### Entocuneiform

This bone is proximodistally as tall as the ectomesocuneiform (Fig 26G–26L). In dorsal view they show a protuberance on the plantodistal margin that is stronger in BAT10’12.D6-130. In proximal view the articulation facet for the navicular-cuboid is flat and shows a teardrop-shape. The medial surface is flatter than the lateral one. This surface sports a depression. In distal view the articular facet with the metatarsal is wide and oval-shaped.

#### Metatarsal III-IV

The metatarsals are medium in robustness relatively to other giraffids, with a robustness index (RI) that ranges from 8,69 to 10,33. They are more slender than the metacarpals. In dorsal view there is a small step between the medial and lateral proximal facets, located at slightly different levels (Fig 27). The common digital artery runs superficially on the dorsal surface of the metatarsal III-IV within a canal (the metatarsal sulcus) that is distally open. This condition of the common digital artery is classified as “moschid-type”[87], meaning that it is superficial but not as superficial as in derived bovids. The metatarsal sulcus is proximally shallower and distally deeper. The proximal canal for the extensor digitorum lateralis is broad. On the plantar side there is a proximocentral fossa that appears to be deeper in BAT10’09.D5-53. In the three specimens a plantar concavity can be observed on the proximal part of the diaphysis. This concavity is shallow and disappears distally, where the plantar surface is flat. In proximal view there is an asymmetry between the lateral and the medial profile. The kidney-shaped articulation facet for the ectomesocuneiform is similar in size to the navicular-cuboid facet, which is semicircular. The entocuneiform facet is relatively big and teardrop-shaped and it stands out over the plantar profile. The metatarsal broadens distally on the last fifth of the bone. The distal trochleas are wide and low and the keels are plantarly narrower and thinner. The dorsal profile of the distal end of the diaphysis is slightly convex. The keels do not extend plantarly into the shaft.

#### Lateral metapodials II and V

Metapodials II and V are very reduced (Fig 28). Located proximomedially, next to the metapodial III-IV but not attached to it, they can be recognized as a thin and elongated tubercle between the medial cuneiform facets. There are specimens that only preserve the proximal thicker part (e.g. BAT10’12.D6-97) while others are composed of a proximal part and a more distal part (e.g. BAT10’12.D6-108). The proximal part is rugous and thicker than the thin and stylet-shaped distal part. The complete lateral metapodials do not represent more than 1/8 of the total metatarsal III-IV length.

#### Metatarsal sesamoid

The only one preserved attaches to the dorsoventral surface of the metatarsal in a central position. It is rugous and ovoid shape (Fig 27).

#### First phalanx

In dorsal view the first phalanges resemble a cone, wider in the proximal part, and narrower distally (Fig 29A–29E). In both lateral and dorsal view, the first phalanges are transversally wide, with strong proximal and distal ends, resembling type A of Köhler in having athe facet of the distal articulation being visible from the dorsal side, and type C in having a stronger proximal end [88].The proximal articular surface is transversally wider than the distal joint. In proximal view proximal facet is heart-shaped and asymmetric, with the inter-digital half shorter and with a rounded profile and he lateral half longer and with a straight border. The central groove is not too deep and it is relatively wide. On the palmar/plantar surface there are two long and rugous bulges with a sulcus between them, more or less deep depending on the specimen. These bulges represent proximal areas for ligament insertion, where the collateral and the sesamoid ligaments, as well as the interosseous muscle tendon attach. The interdigital surface also shows some rugous areas for the interdigital ligament, although this surface is almost even, suggesting than it was not very strong. The outline of the dorsal surface is flat or slightly concave. The distal articulation is well rounded from its lateral aspect and its facet is not visible form the dorsal view. The concave palmar/plantar curvature begins from the middle of the shaft. The distal joint is trapezoidal and asymmetric with the lateral half almost twice wider than the inter-digital one. Two groups of phalanges can be differentiated according to their general dimensions. We analyzed the autopodium of a complete connected specimen and it appears that longer phalanges may be interpreted as anterior phalanges, and the shorter as posterior phalanges but there are no critical morphological or metrical differences between them. This homogeneity agrees with that observed in the related measurements of the distal epiphyses of both metapodials in particular those of the pulleys.

#### Second phalanx

In both lateral and dorsal view, the second phalanges are very robust and transversally wide, with strong proximal and distal ends (Fig 29F–29J). The proximal articular surface is transversally wider than the distal joint. In proximal view the joint for the first phalanx is sub-quadrangular in shape. The proximal articular surface is slightly concave and the crest that divides the two articulation sides is soft. The post-articular platform is proximally elongated and the dorsal extensor process is prominent and rugous. Proximally, the impressions for the origin of the interdigital (or cruciate) ligaments and the insertion of the flexor tendon are weak. The dorsal extensor process is high and rugous. The distal articular surface is triangular and asymmetric, with a reduced inter-digital part.

#### Third phalanx

In both lateral and dorsal view, the third phalanges are long and transversally narrow (Fig 29K–29S). From a lateral view they show a triangular outline. There is a well-developed dorsal process for the insertion of the extensor digitorum (longus or comunis depending if it is the anterior or the posterior phalanx) that is even more prominent than in *Giraffa*. The articular facet is rounded, with the exterior part larger than the inter-digital part. Both are concave and are inclined towards the inter-digital side of the phalanx. There is a moderately strong plantar process for the insertion of the deep flexor tendon. There is a small rhomboidal facet for the distal sesamoid. The plantar surface of the phalanges has the shape of an isosceles triangle.

#### Sesamoids

The proximal exterior and larger sesamoids show facets for the metapodial semicircular and proximodistally shorter (Fig 30A–30F), while the interdigital ones are elongated and oval (Fig 30G–30L). They also differ in the size, being the interdigital sesamoids dorso-palmarly/plantarly taller. The distal sesamoids show two facets (Fig 30M–30R), both oval in shape. The interdigital one is larger than the lateral.

## Results of the phylogenetic analysis

Our data matrix (S1 File, S4 Table) includes 31 taxa and 111 characters (cranial, dental, mandibular, and postcranial; S2 Text). The Maximum Parsimony (MP) search produced a single Most Parsimonious Tree (MPT) of 274 steps (CI = 0.675; RI = 0.780; Fig 31). The bremer support for most of the branches is low. This tree recovers a monophyletic Giraffidae, the most basal off-shoots of which are the stratigraphically older *Canthumeryx sirtensis* and *Georgiomeryx georgalasi*. The remaining taxa are grouped into two clades: a group of stem-giraffids including *Injanatherium hazimi*, *Injanatherium arabicum*, *Giraffokeryx punjabiensis* and *Giraffokeryx primaevus*, and the large group of crown-giraffids (node D) in which the forms found in Spain *Decennatherium pachecoi*, *Decennatherium rex* (urn:lsid:zoobank.org:pub:68375E3F-6C6F-45F9-9F89-8B10D3608C6A) and *Birgerbohlinia schaubi* are included. The crown-Giraffidae split into two groups, one of them containing *Giraffa* + *Bohlinia attica* (node E) and the other one (node F) including a *Palaeotragus*-clade (node H) basal to a large group (node I) that clusters *Okapia johnstoni*, ‘*Samotherium’ sinense* and *Schansitherium tafeli* as successive sister groups to a *Sivatherium* / *Samotherium*-clade (node J, sivathere-clade hereafter) that is composed of *Decennatherium*, *Birgerbohlinia*, samotheres and sivatheres. Here, *Decennatherium*, including *D*. *rex* + *D*. *pachecoi* (node K), branches off as the sister group of the clade that includes the late Miocene and Plio-Pleistocene *Samotherium major*, *Samotherium boissieri*, *Alcicephalus neumayri*, *Birgerbohlinia schaubi*, *‘Sivatherium’ hendeyi*, *Helladotherium duvernoyi*, *Sivatherium maurusium/olduvaiense*, *Sivatherium giganteum*, *Bramatherium perimense* and *Bramatherium megacephalum*. The distribution of character states for the internal nodes and the autapomorphies of the terminals are presented in Table 1.

**Table 1:** Distribution of autapomorphic character states for the internal nodes. Node K includes *Decennatherium*.

Node / Taxon	Character (State)
**Node A**	2(1); 3(1); 7(1); 8(1); 9(1); 10(1); 64(0)
**Node B**	1(1); 62(1); 65(1); 72(1); 96(1); 109(1)
**Node C**	70(0)
**Node D**	59(2); 77(1); 84(2); 93(1); 109(2)
**Node E**	76(1); 87(3); 92(2); 101(4); 105(1)
**Node F**	52(1); 70(2)
**Node G**	20(2); 34(2); 49(1); 81(2)
**Node H**	15(1); 20(3); 21(1); 25(1); 81(2)
**Node I**	78(0); 89(2); 99(1); 100(1)
**Node J**	13(1); 16(1); 26(1); 28(2); 49(1)
**Node K**	79(1); 106(2)
**Node L**	97(1); 98(1); 102(1); 103(1); 105(1)

## Discussion

### Phylogenetic position of *Decennatherium* within the Giraffidae

The Giraffidae is the least inclusive clade of giraffomorphs containing *Canthumeryx*, *Giraffa* and *Okapia*. Among giraffids, the Sivathere-clade (node J) is diagnosed in our MPTs by five synapomorphies (Table 1) that include mainly cranial features related to the ossicones, as the development of the ridges over the surface of the ossicone (ch. 16) and the position of the anterior and posterior ossicones (ch. 26, 28). We define the *Sivatherium*-clade as the least inclusive clade of crown-giraffids that contains *Decennatherium* and *Sivatherium*. Our MPT shows a close relationship between samotheres and sivatheres, with *Birgerbohlinia schaubi* appearing as a basal branch of the derived *Sivatherium* + *Bramatherium* clade (node L), a strongly supported position (Fig 31) that agrees with previously published cladistic works [15] and is similar to the group described by Hamilton (1978) [10]. This result confirms the close relationship of the Iberian giraffids to the African sivatheres, as previously proposed by Crusafont (1952) [17], Morales and Soria (1981) [89] and Montoya and Morales (1991) [90].

The Iberian *Decennatherium rex* is the earliest example of the basic *Sivatherium*-like ossicone plan (Figs 3–5, 32 and 33). This plan consists on a four-ossicone pattern with two small anteriorly-oriented frontal ossicones plus two much larger, caudally-oriented and curved, fronto-parietal ossicones covered by numerous longitudinal ridges (characters 1–47, Figs 3–5, 32 and 33). *Birgerbohlinia schaubi* shows larger versions of the *Decennatherium rex* ossicones, and the gigantic *Sivatherium giganteum* and *S*. *maurisium*/*olduvaiense* sport the most extreme versions of this plan (Figs 32 and 33). Basal to the *Sivatherium*-clade, *Schansitherium tafeli* also has four ossicones but contrary to the *Sivatherium* ossicone-plan its anterior ossicones are much larger and straight, and have their bases fused with the posterior ossicones. Also the posterior ossicones of *Schansitherium* are straight instead of curved (Fig 33B). The stem-giraffid *Giraffokeryx punjabiensis* from the Middle Miocene of India and Pakistan has posterior ossicones superficially similar to that of *Decennatherium rex*, but contrary to the Iberian form its anterior ossicones are postero-laterally oriented and slightly anteriorly placed (Fig 34C). Thus, a four-ossicone pattern of head gear has appeared more than once within the Giraffidae with at least four types of it, two of them characterizing forms outside the crown-Giraffidae (*Inajanatherium* and *Giraffokeryx*), a third one being apparently autapomorphic for *Schansitherium*, and finally the *Sivatherium*-plan itself, which became the most successful and long-lived one. Taking into account the phylogenetic position of *Schansitherium* as the latest sister-group to the *Sivatherium*-clade it could be hypothesized the presence of four ossicones in *Schansitherium* + node J as a basal homology between the two clades that derived for the one part in the *Schansitherium* morphology and for the other part in the *Sivatherium*-plan. The presence of ossicones with bumps in *Decennatherium rex* (ch. 17) further connects *Decennatherium* to the *Sivatherium* lineage as this bumps are very similar to those observed in the ossicones of *Sivatherium maurusium/olduvaiense* (Fig 32Q–32U) [62].

The postcranial skeleton of *Decennatherium rex* is more primitive than that of true sivatheres (node L) more similar to that of the samotheres, especially to *Alcicephalus neumayri* (Fig 35L) [15, 33, 89]. A samothere-like postcranial is less robust than that of sivatheres, the metapodials of which reach really high robustness indexes (Figs 36 and 37, ch. 94) [86]. Thus, samothere (and *Decennatherium*) skeletal pattern is primitive compared to the derived pattern of sivatheres. The sivathere robustness goes together with a shortening of the limbs [1]. The robustness and morphology of the limbs of *Decennatherium rex* are intermediate between that of the early Vallesian *Decennatherium pachecoi* and that of the Turolian *Birgerbohlinia schaubi*, a basal true sivathere in which the sivathere-like shortening and increased robusticity of the limbs can already be fully appreciated. The next step is represented by the metapodials of the Plio-Pleistocene ‘*Sivatherium’ hendeyi*, with *Sivatherium giganteum* and *Sivatherium maurusium/olduvaiense* showing the maximum example of this skeletal pattern (Fig 35).

*Decennatherium rex* and *D*. *pachecoi* are sister-taxa, with *Decennatherium pachecoi* stratigraphycally older. Even with no skull elements that can be reliably attributed to *D*. *pachecoi* [15, 89] they share dental and postcranial morphological features (ch. 79 (1); ch. 106(2)). Both species can be differentiated by morphological differences in the p3 and also by the higher overall robustness of *D*. *rex* skeleton (ch. 78, ch. 81–82, ch. 99) (Fig 11).

A *Palaeotragus*-clade (node H) appears as the sister group of the node I, in which the extant *Okapia johnstoni* branches-off as the most basal taxon (Fig 33A). Palaeotragines are characterized by ossicones in a more medial position (ch. 20–21, 25) with a very smooth surface and pointy tips that may show apical polish (Fig 34F and 34G)[93], as well as a molarized p3 with a complete lingual wall (ch. 81). Other studies differ from ours [15], linking *Okapia johnstoni* to the Palaeotraginae as a basal offshoot of the *Palaeotragus*-clade. The differences in the phylogenetic position of *Okapia* between the different maximum parsimony analyses is due to the differences in the number of characters and taxa used in the analysis, and should therefore be regarded as working hypotheses that will benefit from further phylogenetic tests. Clade F (palaeotragines + node I) is diagnosed by the elongation of the diastema (ch. 70) and a derived masseteric morphology (ch.52).

Extant and extinct *Giraffa* (Figs 33D, 33E, 35P and 35Q) plus *Bohlinia attica* (Fig 35O), cluster together in the sister clade to the rest of the crown-Giraffidae. All of these giraffine species show a characteristic elongation of the cervical vertebrae (ch. 87) [1, 71–73], an extreme limb elongation (Fig 38, ch. 96) and a reduction of the robustness of the radius (ch. 92). Thus, what we see in the extant Biosphere is, according with our MPT, a single remnant from each of the two main lineages of the crown-Giraffidae (nodes H and F).

Our results agree with other recent studies [1, 15] in considering the depth of the longitudinal palmar hollowing of the metacarpals containing the palmar interosseous muscles and the specialized tendinous derivatives of these muscles as an important set of characters for understanding giraffid evolution (ch. 97–98, 102–103, Fig 39). We can discriminate several groups of taxa with very shallow troughs (e.g. *Giraffa camelopardalis*), very deep troughs (e.g. *Bohlinia attica)*, and medium troughs (e.g. *Decennatherium rex*) (Fig 39). Solounias (2007) [1] linked *Birgerbohlinia schaubi* with *Bohlinia attica* according to their very deep troughs. However, in our MPT this is recovered as a parallelism between these two taxa. In our MPT the presence of very deep troughs links *Birgerbohlinia schaubi* to other species that also share this feature: *Sivatherium hendeyi* from Langebaanweg, (South Africa) [63] (Soria pers. com.), *Helladotherium duvernoyi* from Pikermi (Greece) and Cucuron (France), and *Bramatherium megacephalum* from Dhok Pathan (Pakistan).

The late Miocene was a time of extensive giraffid diversification, and representatives of this family are found in numerous late Miocene localities throughout all Eurasia and Africa. Some localities have at least eight different species appearing in a single site as is the case of Samos (Greece), were several giraffids coexisted with many bovids and other ‘ungulates’ [1]. In the Iberian Peninsula (Fig 40) the first record of the family is the early Vallesian *Decennatherium pachecoi* [15, 17, 89], chronostratigraphycally followed by the late Vallesian *Decennatherium rex* and the Turolian *Birgerbohlinia schaubi* [28, 90]. The youngest records of the Sivathere-clade are the Plio-Pleistocene ‘*Sivatherium’ hendeyi* from Langebaanweg (South Africa) [63], the Plio-Pleistocene *Sivatherium olduvaiense/maurusium* from Kenya, Tanzania and Ethiopia, and the Pleistocene *Sivatherium giganteum* from the Siwalik Hills (India and Pakistan).

The recovery of *Decennatherium* as the most basal member of this clade extends its paleobiogeographic range to the Iberian Peninsula, and its chronostratigraphical range to the beginning of the late Miocene. The recovery of *Decennatherium* in the Iberian Peninsula also points to the existence of migration / vicariance events of sivathere / samothere genetic pools between the northern and southern margins of the Mediterranean Sea that probably took place even before the beginning of the late Miocene.

### Sexual dimorphism of the cranial appendages of *Decennatherium rex*

There are six calvaria of *Decennatherium rex*, all with ossicones, in the BAT10 sample. Three of them are morphotype I and the other three are morphotype II. Similar states of dental wear do not correlate to ossicone morphotype, excluding ontogeny as the factor behind ossicone development and morphology. We suggest that each morphotype represents a different gender in *Decennatherium rex*. In this scenario, following the trend of putative smaller, lesser-developed female ossicones, morphotype I skulls correspond to female individuals and morphotype II correspond to males (Figs 3 and 4).

Sexual dimorphism of ossicones in the extinct Giraffoidea is typically difficult to identify since isolated ossicones, frontlets bearing more or less complete ossicones, and the occasional nearly complete skull usually do not indicate the sex of the individual [94–99]. However, among living Giraffidae, cranial appendages of female *Giraffa* are smaller than those of the males (Fig 34E).Thus, *Decennatherium rex* would be similar to extant *Giraffa* in the gender-related pattern of development of the cranial appendages. There are probably more examples of *Giraffa*-like dimorphism in the *Sivatherium*-clade. Harris [63] pointed that the recovery of gracile ossicones of *Sivatherium* spp. may indicate that some females may have carried cranial ornamentation. Singer and Boné [62] stated that sexual dimorphism exists in the ossicones of *Sivatherium* spp., with males possessing the larger and more twisted or complex head gear and females sporting lighter and simpler ossicones. Churcher [94] stated that it is likely that cranial ornaments were present in both sexes of primitive giraffids, and may have served in specific recognition or temperature regulation when originally developed with their use in intraspecific male sparring or combat developing later. Taking into account the phylogenetic structure of the Giraffoidea [1] it seems plausible to think that having armed females could be the primitive condition for this lineage of giraffomorphs, but this has to be more thoroughly studied.

### Dietary adaptations

Following Solounias [1] we have analyzed the shape and size of the masseteric fossa (ch. 52–53), the shape of the premaxillae, and other cranial and masticatory features related to feeding behavior. Overall grazers have a larger masseteric fossa than browsers [84, 100], and most giraffid species have angles that are similar to those of a mixed feeder / browsing bovid [1]. The largest masseteric fossae recorded were those of *Samotherium major* and *Samotherium sinense*, were the skull resembles Alcelaphini grazers in lateral view [1]. *Decennatherium rex* has α values (ch. 52) that range from 6,275° to 10,76° (Fig 7). These fall within the middle range of the analyzed giraffids, in the same category as *Schansitherium tafeli*, *Palaeotragus microdon* and *Samotherium boissieri* [1]. Only *Samotherium major* shows values over 10°, while *Canthumeryx sirtensis*, *Okapia johnstoni* and *Giraffa camelopardalis* show low angle α values (5°). Regarding β values (ch.53) *Decennatherium rex* ranges from 74,175° to 109,37° (Fig 7). These are close to those of *S*. *tafeli* (89,38°-92,27°) and *S*. *major* (95,49°-95,83°) while the values of the rest of giraffids in our analysis were lower. This indicates that *Decennatherium rex* has a relatively larger masseteric fossa than most giraffids and may indicate more grazing habits for this species. *Decennatherium rex* also shows other grazer masticatory features such as an enlarged maxilla and a large masseter profundus muscle attachment site. Also, in grazers this enlarged maxilla tends to move the orbit posteriorly, starting above the M3 position or further back as in *Decennatherium rex* [84]. However the shape of the premaxillae in *Decennatehrium rex* (Figs 3 and 4) is intermediate between the pointy browser shape of *Palaeotragus coelophrys* and the square grazer shape of *Samotherium major* [83, 93]. Research on fossil giraffid paleodiet using three independent methods including both tooth microwear and premaxillary analysis [100] concluded that *Bramatherium megacephalum*, *Sivatherium giganteum* and *Samotherium major* were grazers while *Giraffokeryx punjabiensis*, *Palaeotragus* spp. and *Samotherium boissieri* were seasonal mixed feeders, with *Alcicephalus neumayri* characterized as a seasonal mixed feeder and *Giraffokeryx primaevus*, *Bohlinia attica*, *Giraffa camelopardalis*, *Okapia johnstoni* and *Helladotherium duvernoyi* as browsers. This work suggested that there is a high dietary heterogeneity among the Giraffidae [100] and that the traditional characterization of giraffids as browsers needs to be re-evaluated. Other works using outer and inner mesowear analysis [101] resulted in most giraffids characterized as having a browsing and mixed feeding diet along the north Chinese and Greek localities of the Pikermian biome. Domingo et al. [102] analyzed the isotopic composition of the enamel of *Birgerbohlinia schaubi* from Puente Minero (MN11) and the results suggested that *Birgerbohlinia schaubi* was likely a browser with δ^13^C values indicative of woodland foraging. The very high δ^18^O_CO3_ and δ^18^O_PO4_ values in *Birgerbohlinia schaubi* relative to other mammals from Puente Minero may indicate that this sivatheriine obtained much of its water from highly evaporated leave water as suggested by Cerling et al. [103] for the extinct *Palaeotragus* and Levin et al. [102, 104] for modern giraffids. Thus, data suggest that while most primitive giraffid species were browsers and that grazing and mixed feeding took place in forest and woodlands during the Miocene before the expansion of savannas and grasslands [1], late Miocene giraffids had a wider range of dietary habits than the living *Giraffa* spp. and *Okapia johnstoni* [101]. In this context, further microwear, mesowear and isotopic analysis would be needed to establish the condition of *Decennatherium rex* as a grazer or a mixed feeder more accurately.

### Body mass

Estimates of body size are important biological descriptors, as they indicate important traits as growth rates, metabolism and behavior [105, 106]. We estimate a height to the cross of ~2 meters and ~2,8 m to the top of the ossicones, and a length of 2,9 m to the front of the premaxilla to the back of the pelvis (Fig 41). We follow Campione et al. [105] and use the minimum circumference (thinnest region along the diaphysis) of the humerus and femur to estimate the body mass of *Decennatherium rex*. The results ranged from 776,666 1 to 1367,1190 Kg (Table 2), very similar to the weight of an adult *Giraffa* and heavier than an adult *Okapia*.

**Table 2:** Body mass predictions for *Decennatherium rex* using a right humerus and femur and a left humerus and femur.

Average (Right-Left)	Lower	MR	Upper	PPE
Equation (1): BM = f (HCF) + no phylo correction	776,66	1044,32	1311,98	25,62
Equation (2): BM = f (HCF) + phylo correction	819,74	1093,43	1367,11	25,02
Equation (5): BM = f (HC, FC) + no phylo correction	799	1064,66	1330,11	24,93
Equation (6): BM = f (HC, FC) + phylo correction	817	1083,67	1350,51	24,62
**Model Average**	**803**	**1.072**	**1.340**	**25,04**

Columns indicating body mass lower and upper prediction intervals, and percentage prediction error (PPE) for each Campione et al., 2012[105] equation. MR, mid range; BM, body mass; H, humerus; F, femur; phylo, phylogenetic.

## Conclusions

The extraordinary collection of giraffid fossils from the late Miocene site of BAT10 (Madrid, Spain) allow us to describe the new species *Decennatherium rex* and carry out a phylogenetic analysis of the Giraffidae using the largest morphological data matrix ever compiled for this ruminant clade. The skull anatomy of *Decennatherium* was unknown so far, and through the description of its skeleton, *Decennatherium rex* sp. nov. helps to reassess the morphological evolution and phylogenetic relationships of both the genus *Decennatherium* and the clade of girafids in which it belongs. While several works linked *Decennatherium* to samotheres, our study reveals a more complex image where this Iberian genus appears as the basal off-shoot of a large sivathere-clade (also including samotheres). This group is diagnosed mainly by cranial features, and is defined as the least inclusive clade of crown-giraffids that contains *Decennatherium* and *Sivatherium*. The inclusion of *Decennatherium* as the most basal branch of this clade extends the chronostratigraphic and paleobiogeographic ranges of sivatheres / samotheres to the early late Miocene and to the Iberian Peninsula in Europe, respectively. Hence the Sivathere-clade is one of the most successful and long-lived of all the Giraffidae. Also, *Decennatherium* is the earliest known evidence of the *Sivatherium* ossicone-plan, a pattern of four ossicones that consists of two small, anteriorly-oriented, frontal ossicones and two much larger, caudally-oriented and curved, fronto-parietal ossicones that are covered by numerous longitudinal ridges. Through *Decennatherium* this plan can be traced back to the late Miocene in Western Europe, disappearing with the last gigantic sivatheres at the end of the Pleistocene. This large cranial headgear would be of high relevance in establishing and maintaining the dominance of the members of this clade from the Miocene to almost the verge of present times.

*Decennatherium rex* was probably sexually dimorphic. Females were horned, but their ossicones were smaller and lesser developed than those of males. In this regard, *D*. *rex* was similar to extant *Giraffa* and different from *Okapia*, the females of which are hornless.

The premaxilla shape of *Decennatherium rex* is intermediate between a pointy browser shape and a full square grazer morphology. Other characters (size of masseteric fossa and caudal displacement of the orbit) also point to mixed-feeding habits in *D*. *rex*, as was probably common among Miocene giraffids, which had a wider range of feeding strategies than those present in extant *Giraffa* and *Okapia*. Microwear, mesowear, and isotopic analyses will potentially help refine this analysis.

The estimated body mass for *Decennatherium rex* (slightly less than 1000 Kg) indicates that this giraffid was smaller than the extant *Giraffa*, but still was considerably larger than *Okapia*. It also indicates a size increase from the most basal members of the Sivathere-clade to the gigantic and derived Plio-Pleistocene representatives of the group.

## Supporting information

S1 File Matrix. (TNT)

S1 Table Measurements (cranial, dental) of *Decennatherium rex* sp. nov. from BAT10. Excel sheets in order: **s**kull, ossicones, maxillae, mandibles, canines, incisors, upper deciduous dentition, lower deciduous dentition, upper molars, lower molars, upper premolars, lower premolars.
(XLS)

S2 Table Measurements (postcranial) of *Decennatherium rex* sp. nov. from BAT10. Excel sheets in order: vertebrae, ribs, scapulae, humerus, radius-ulna, carpals, metacarpal III-IV, pelvis, femur, tibia, tarsals and patella, metatarsal III-IV, lateral metatarsals, phalanges, sesamoids.
(XLS)

S3 Table Descriptive parameters of the measurements of the Giraffidae analyzed (cranial, dental, postcranial). Excel sheets in order: skull, ossicones, juvenile mandible, adult mandible, p3, p4, m3, atlas, axis, radius metacarpal III-IV, tibia, phalanges, measurements references.
(XLSX)

S4 Table Matrix. (XLSX)

S1 Text Description of the measurements (cranial, dental, postcranial). Fig 1, Skull measurements; Fig 2. Ossicone measurements; Fig 3, Atlas measurements; Fig 4, Axis measurements; Fig 5, Cervical measurements; Fig 6, Thoracic and lumbar measurements; Fig 7, Rib measurements; Fig 8, Pelvis measurements; Fig 9, Patella measurements; Fig 10. Sesamoid measurements.
(PDF)

S2 Text Character list. (DOCX)
