# Supplementary material for: A new giraffid (Mammalia, Ruminantia, Pecora) from the late Miocene of Spain, and the evolution of the sivathere-samothere lineage
Source: PLoS One. 2017 Nov 1;12(11):e0185378. doi: 10.1371/journal.pone.0185378 (PMC5665556; doi:10.1371/journal.pone.0185378)
Supplement: S1 Text — Fig 1, Skull measurements; Fig 2. Ossicone measurements; Fig 3, Atlas measurements; Fig 4, Axis measurements; Fig 5, Cervical measurements; Fig 6, Thoracic and lumbar measurements; Fig 7, Rib measurements; Fig 8, Pelvis measurements; Fig 9, Patella measurements; Fig 10. Sesamoid measurements. (PDF) [file pone.0185378.s006.pdf]

S1 Text. Description of the measurements (cranial, dental, postcranial).

S1 Text Contents

S1 Text. Figure A. Skull measurements.

S1 Text. Figure B. Ossicone meassurements.

S1 Text. Figure C. Atlas measurements.

S1 Text. Figure D. Axis measurements.

S1 Text. Figure E. Cervical measurements.

S1 Text. Figure F. Thoracic and lumbar measurements.

S1 Text. Figure G. Rib measurements.

S1 Text. Figure H. Pelvis measurements.

S1 Text. Figure I. Patella measurements.

S1 Text. Figure J. Sesamoid measurements.

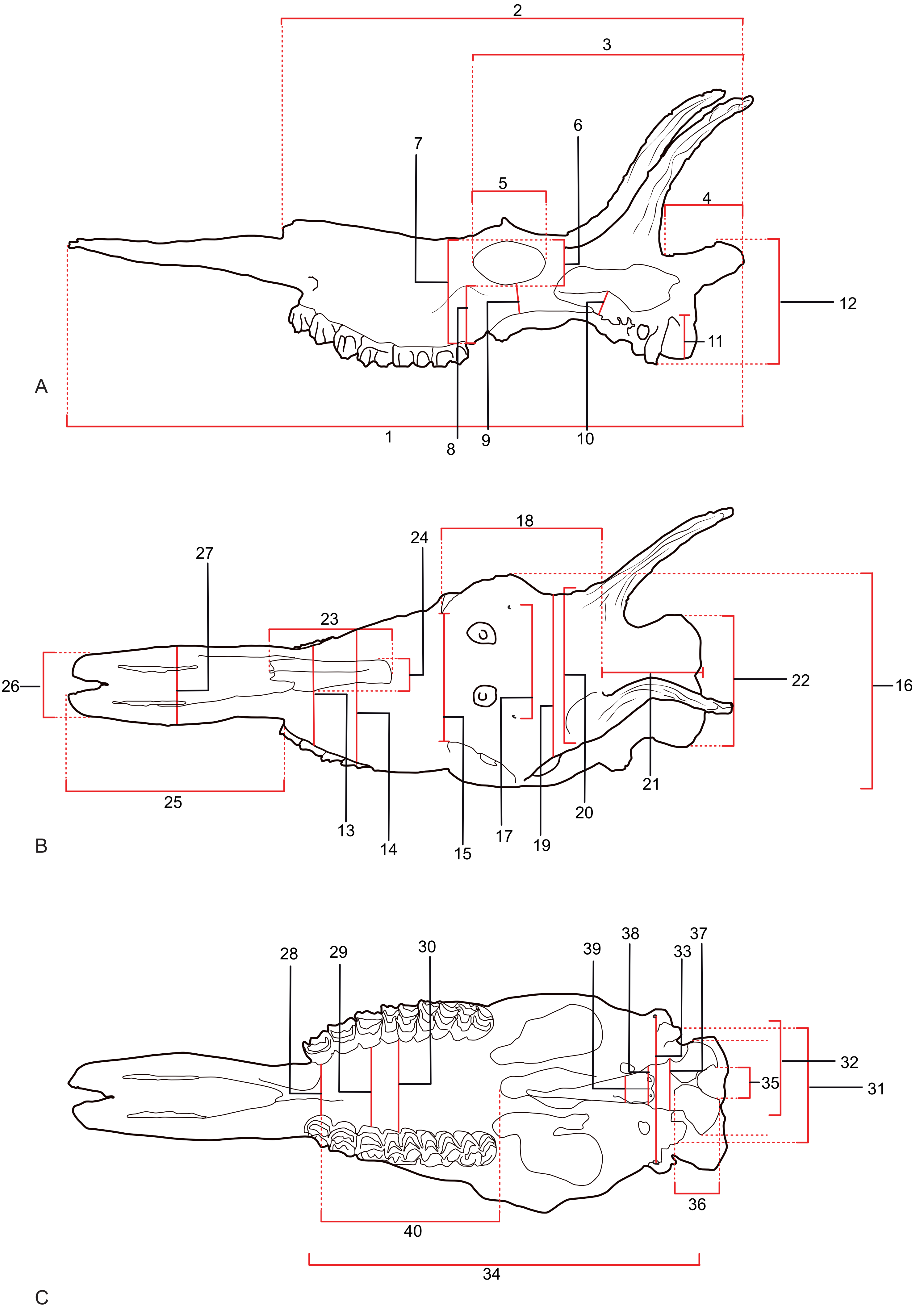
A
1
2
3
4
5
6
7
8
9
10
11
12
B
13
14
15
16
17
18
19
20
21
22
23
24
25
26
27
C
28
29
30
31
32
33
34
35
36
37
38
39
40

S1. Figure A. Skull measurements. 1, Total length of the skull; 2, Length fromnasals to the extremity of the occipital crest; 3, Length from the midpoint above anterior margin of the orbits to the extremity of the occipital crest; 4, Length from the posterior limit of the posteior ossicone base to the extremity of the occipital crest; 5, Orbital length; 6, Orbital height; 7, Height from the alveolar level of the M3 to the upper margin of the orbit; 8, Height from the alveolar level of the M3 to the lower margin of the orbit; 9, Zygomatic arch width; 10, Zygomatic process of the temporal bone width; 11, Occipital condyles height; 12, Height of occipital region from the lowest part of the jugular process to the top of the occipital crest; 13, Width of the maxillae above P2s; 14, Width of the maxillae above M1s; 15, Distance between the anterior orbital margins; 16, Maximum width of the cranial roof; 17, Distance between the supraorbital orifices; 18, Length from anterior orbital margin to a point midway between the ossicones; 19, Minimum distance between the temporal lines; 20, Skull width; outside of the ossicones basis; 21, Length from the point midway between the ossicones to the extremity of the occipital crest; 22, Maximum widt; of the occipital crest; 23, Nasals length; 24, Width of nasals; 25, Premaxillae length; 26, Premaxillae anterior width; 27, Premaxillae maximum width; 28, Palate width between the P2s; 29, Palate width between the M1s; 30, Palate width between the P4s; 31, Distance between the tips of jugular processes; 32, Width of occipital condyles; 33, Width of the skull at the two external acustic meatus; 34, Distance from the anterior border of the P2 to the front edge of the foramen magnum; 35, Width of foramen magnum; 36, Occipital condyles length; 37, Basioccipital maximum width; 38, Bassioccipital width at the level of the posterior tuberosities; 39, Basisphenoid width at the level of the formaen ovale; 40, Distance between the anterior border of the P2 to the nasal choane anterior border.

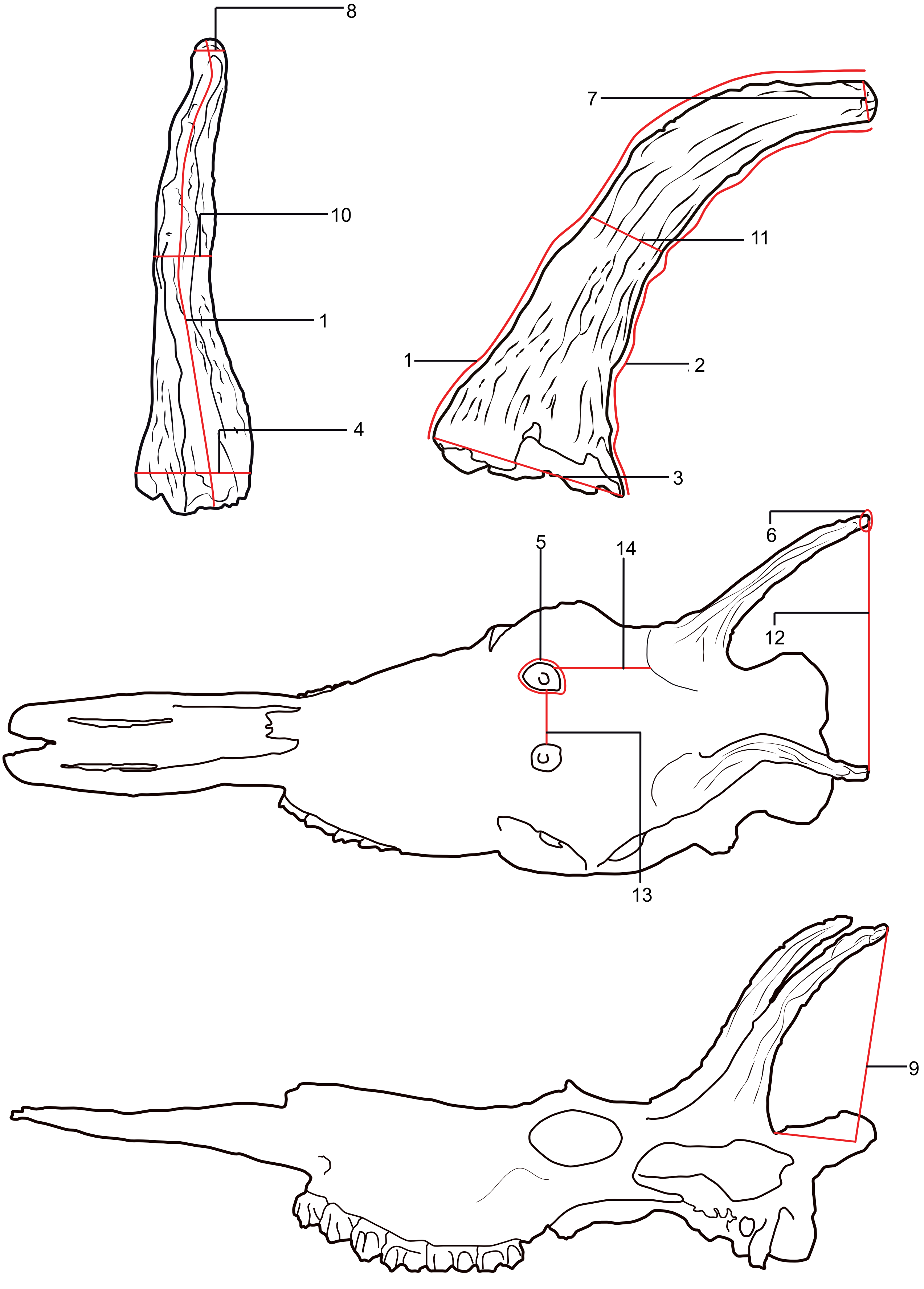

S1. Figure B. Ossicone meassurements. 1, Length of the ossicone (dorsal); 2, Length of the ossicone(ventral); 3, APD at the ossicone base; 4, TD at the ossicone base; 5, Circumference at the ossicone base; 6, Circumference at the ossicone tip;7, APD at the ossicone tip; 8, TD at the ossicone tip; 9, Perpendicular height; of the ossicone from skull roof to tip; 10, TD at the middle of the ossicone; 11, APD at the middle of the ossicone; 12, Distance between tips of of anterior ossicones / Distance between tips of of posterior ossicones; 13, Distance between bases of anterior ossicones / Distance between bases of posterior ossicones; 14, Distance between bases of anterior and posterior ossicones of the same side.

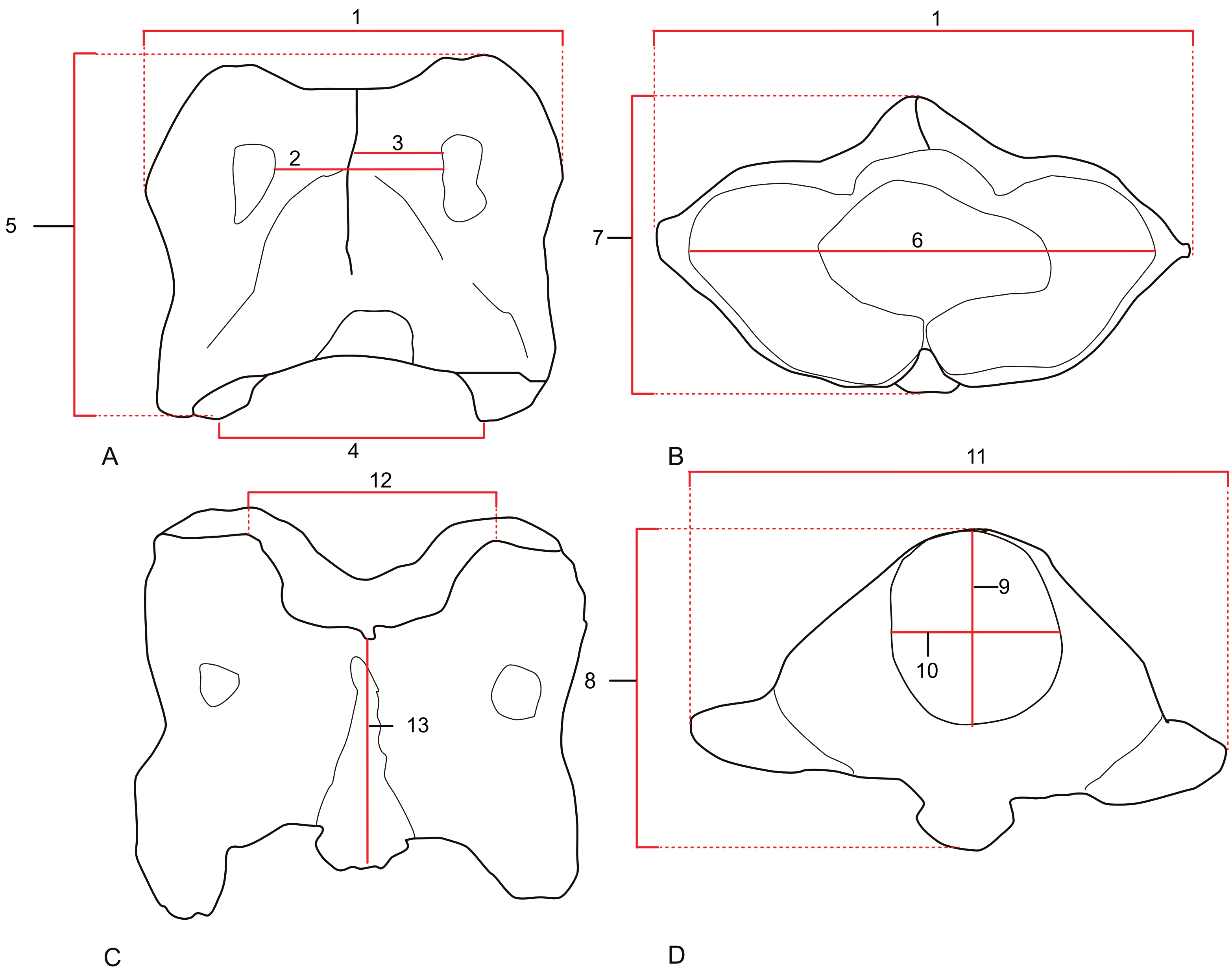

S1. Figure C. Atlas measurements. 1, Maximum width; 2, Distance between the two lateral vertebral foramens; 3, Distance from the lateral vertebral foramen to a point in the midline of the atlas; 4, Distance between the two caudal tubercles; 5, Maximum length; 6, Width of the anterior articular cavity for the occipital condyles; 7, Cranial height; 8, Caudal height; 9, Vertebral foramen height; 10, Vertebral foramen width; 11, Width of the caudal articular surface; 12, Distance between the cranial tubercles; 13, Ventral length from the interarticular indentation to the most caudal part of the ventral tubercle.

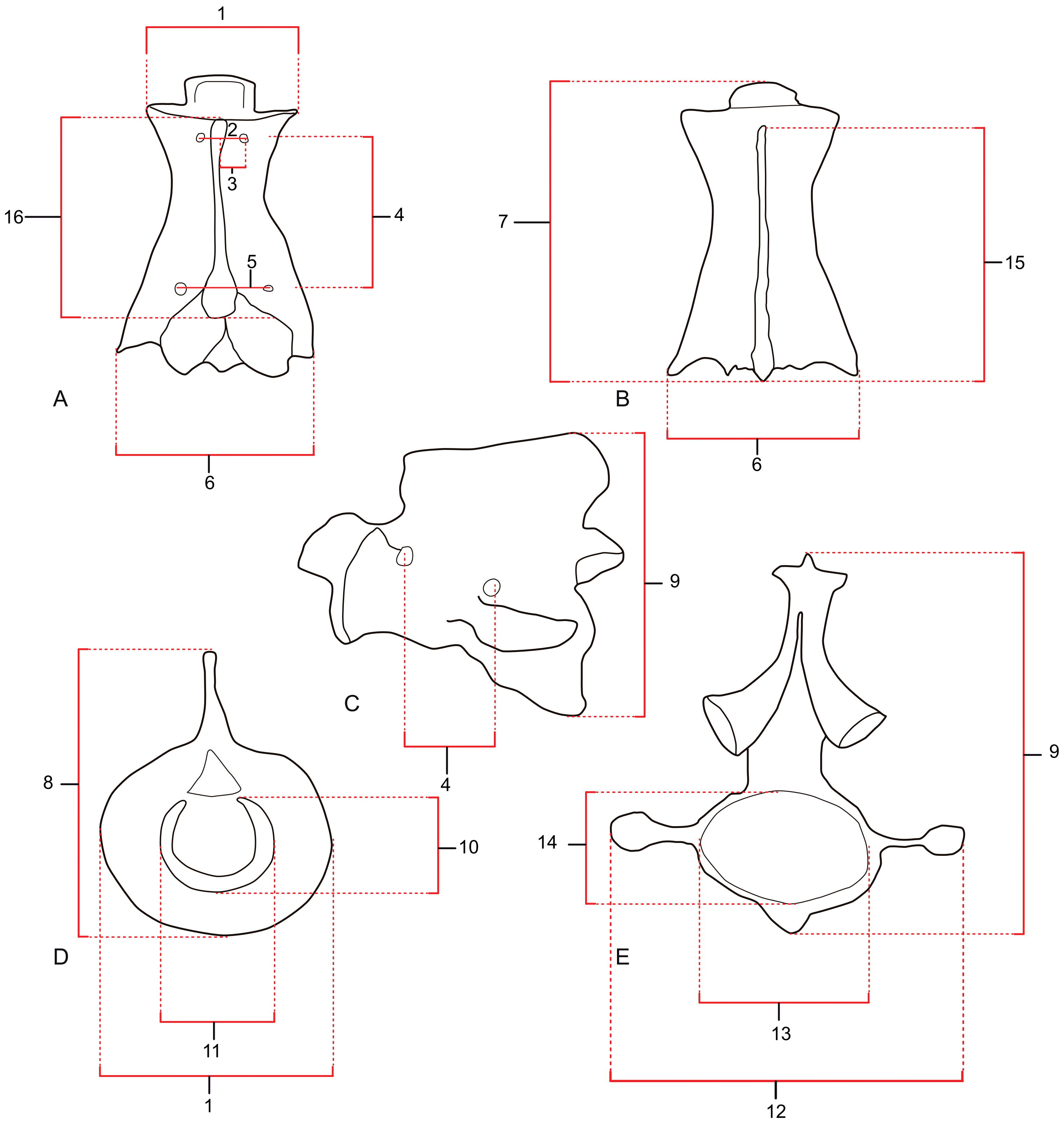

S1. Figure D. Axis measurements. 1, Cranial width; 2, Distance between the two anterior lateral vertebral foramina; 3, Distance from the lateral vertebral foramen to a point in the midline of the axis; 4, Distance between the anterior and posterior lateral vertebral foramina; 5, Distance between the posterior lateral vertebral foramina; 6, Caudal width; 7, Maximum length; 8, Cranial height; 9, Maximum height; 10, Cranial ledge of the vertebral foramen height; 11, Cranial ledge of the vertebral foramen width; 12, Caudal width; 13, Vertebral fossa width; 14, Vertebral fossa height; 15, Ventral crest length; 16, Spinous process length.

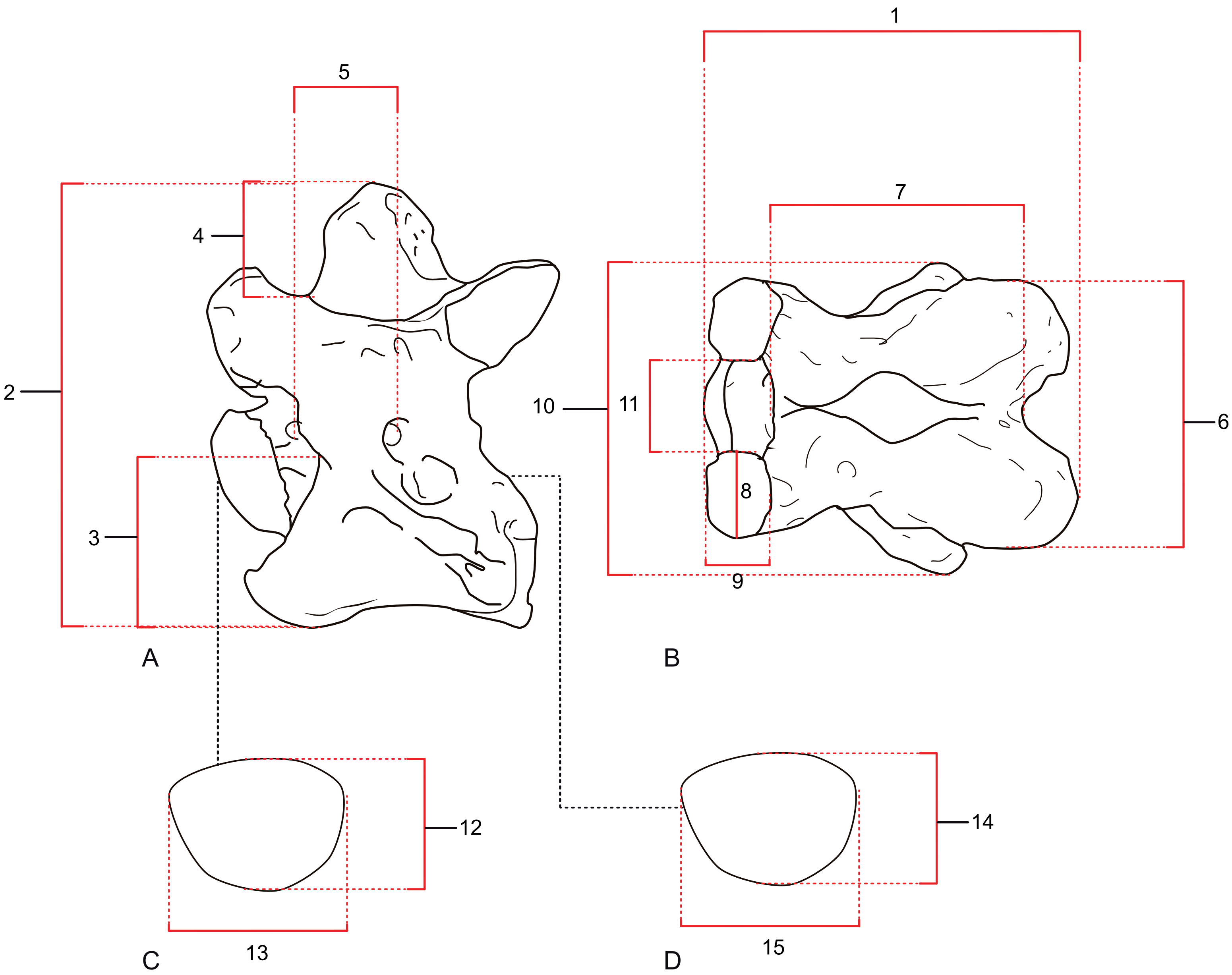

S1. Figure E. Cervical measurements. 1, Maximum length; 2, Maximum height; 3, Transverse process height; 4, Spinous process height; 5, Distance between the anterior and posterior lateral vertebral foramina 6, Distance from the exterior border of the articular processes 7, Dorsal length; along the midline of the cervical 8, Articular facet of the cranial articular process width; 9, Articular facet of the cranial articular process length; 10, Maximum width; 11, Distance between inner sides of the transverse processes 12, Cranial articular facet width; 13, Cranial articular facet height; 14, Caudal articular facet width; 15, Caudal articular facet height.

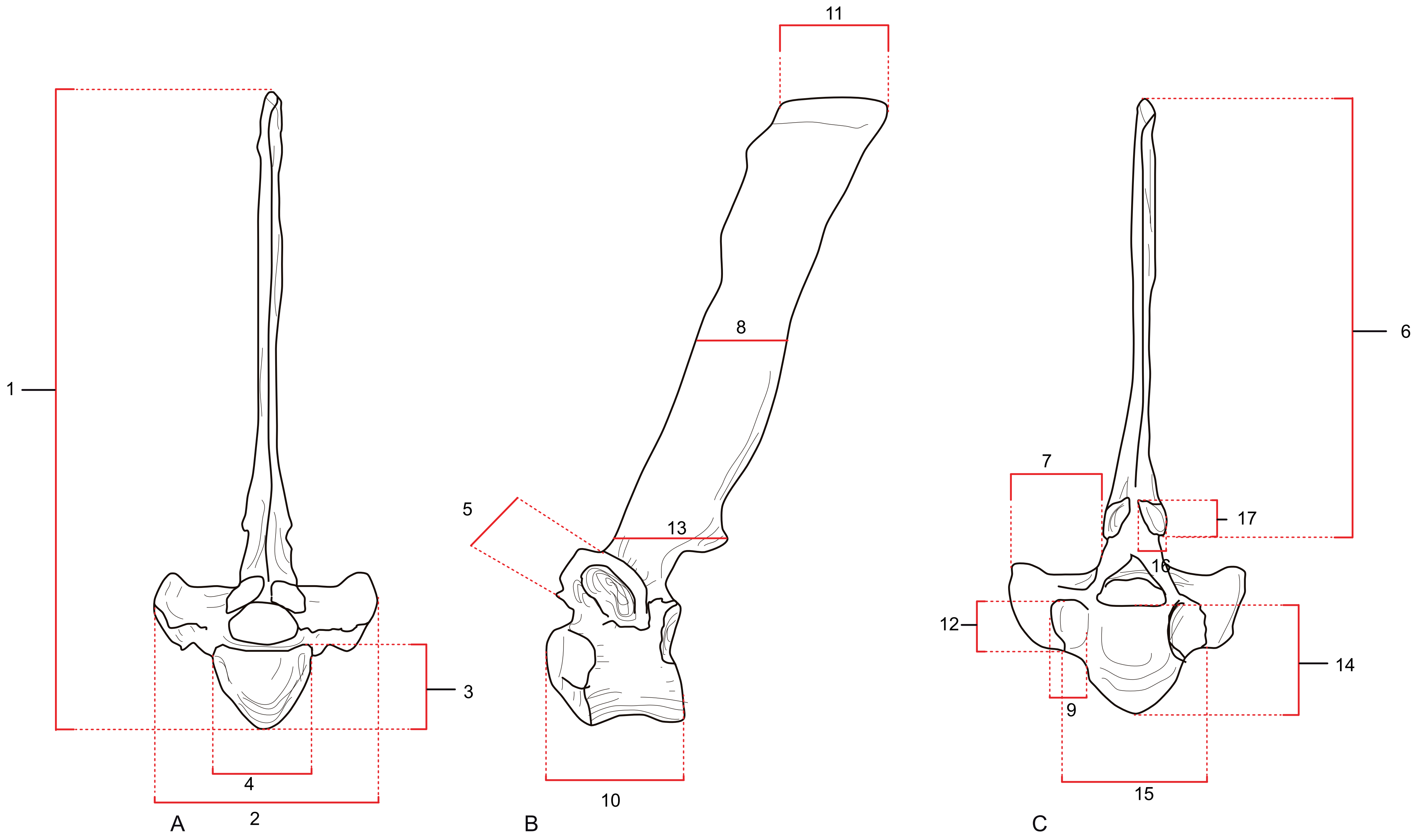

S1. Figure F. Thoracic and lumbar measurements. 1, Maximum height; 2, Maximum width; 3, Cranial articular facet height; 4, Cranial articular facet width; 5, Transverse process height; 6, Spinous process height; 7, Transverse process width; 8, Spinous process length at the middle; 9, Costal fovea width; 10, Vertebral body length; 11, Spinous process distal length; 12, Costal fovea height; 13, Spinous process length at the caudal articular process; 14, Caudal articular facet height; 15, Caudal articular facet width; 16, Caudal articular process width; 17, Caudal articular process height.

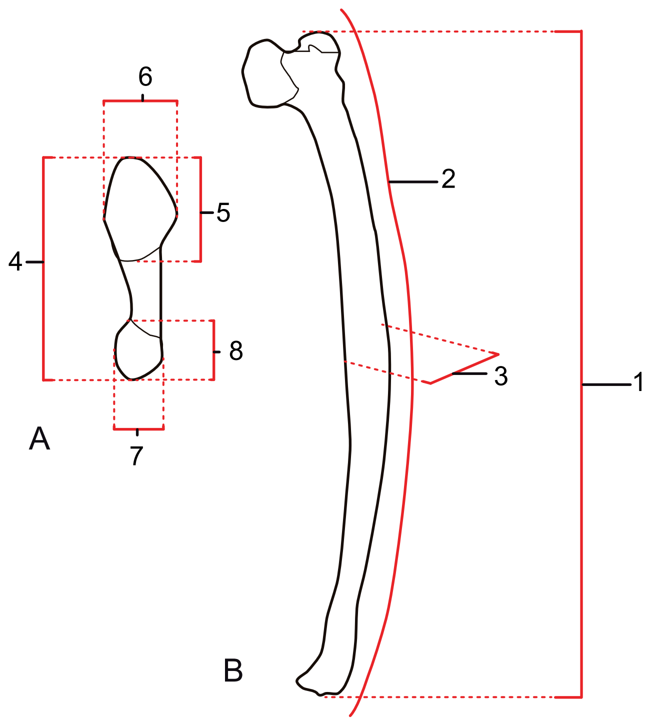

S1. Figure G. Rib measurements. 1, Length; 2, Length of the arch; 3, Craniolateral maximum diameter; 4, Maximum proximal diameter; 5, Head length; 6, Head width; 7, Rib tuberculum length; 8, Rib tuberculum width.

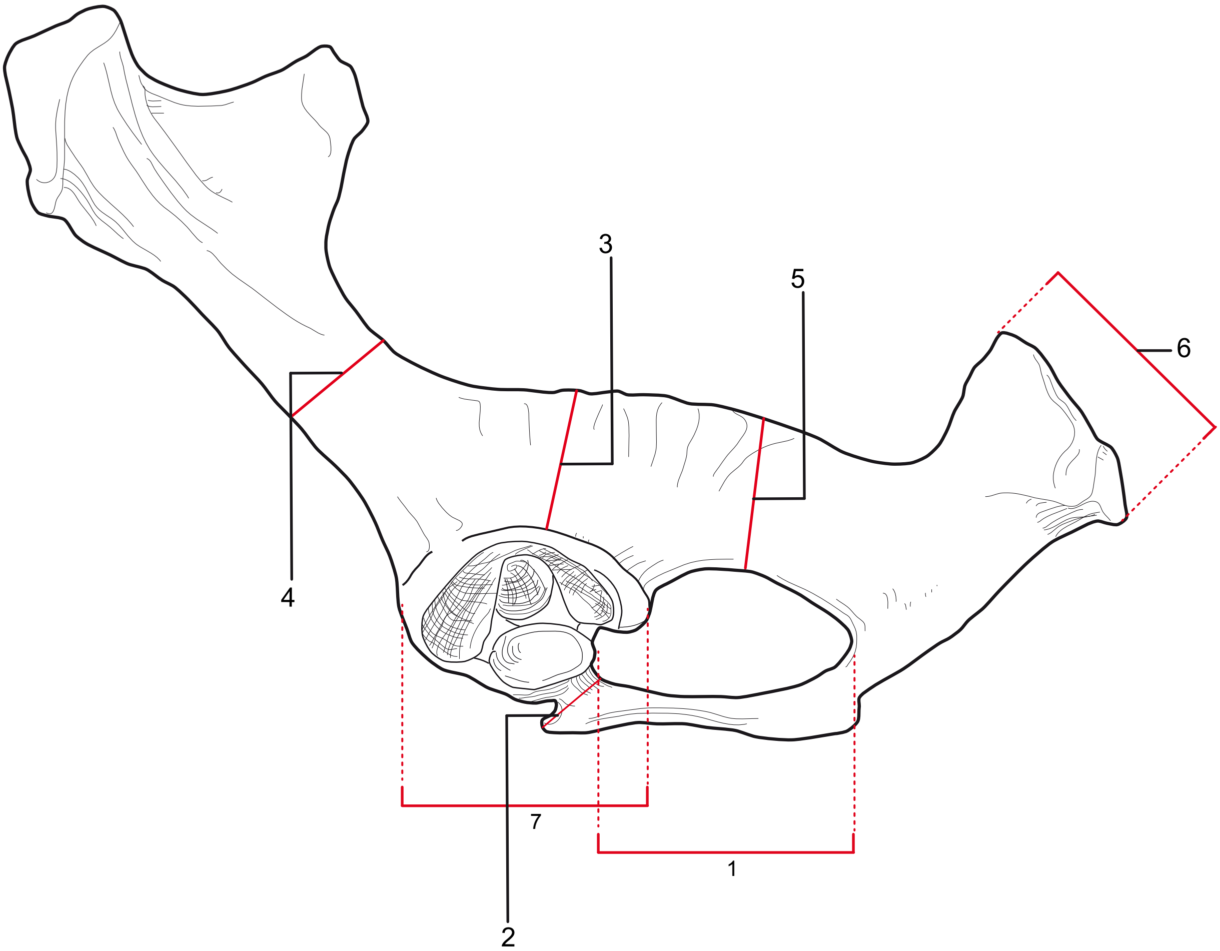

S1. Figure H. Pelvis measurements. 1, Obturator foramen craniocaudal diameter; 2, Pubic spine maximum diameter; 3, Distance from the dorsal side of the ischium to the border of the acetabulum; 4, Illium neck minimum dorsoventral diameter; 5, Ischium body minimum dorsoventral diameter; 6, Ischiatic tuberosity craniocaudal diameter; 7, Acetabulum craniocaudal diameter.

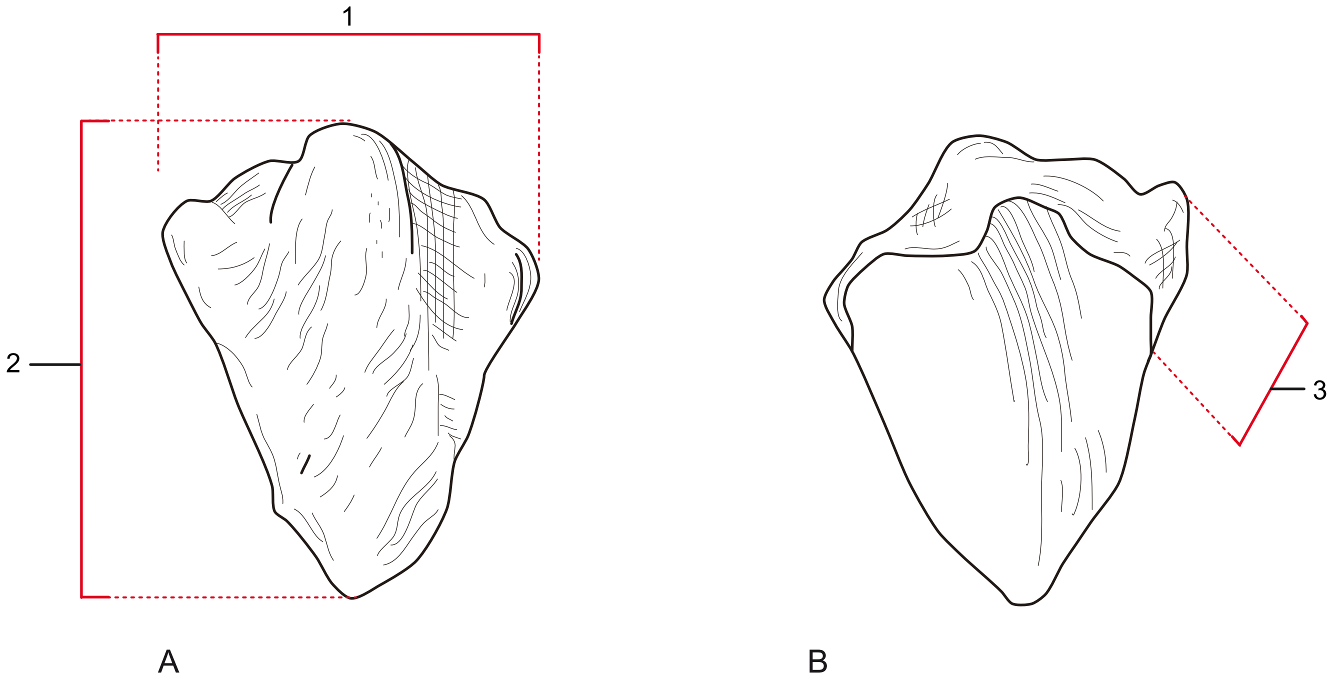

S1. Figure I. Patella measurements. 1, Maximum width; 2, Maximum height; 3, Maximum length.

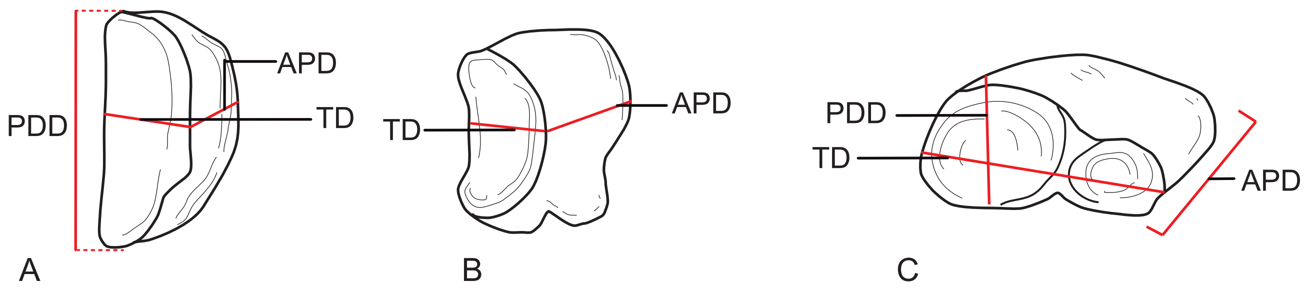

S1. Figure J. Sesamoid measurements. TD, transverse diameter; APD, anteroposterior diameter; PDD, proximodistal diameter.
