# Supplementary material for: A new giraffid (Mammalia, Ruminantia, Pecora) from the late Miocene of Spain, and the evolution of the sivathere-samothere lineage
Source: PLoS One. 2017 Nov 1;12(11):e0185378. doi: 10.1371/journal.pone.0185378 (PMC5665556; doi:10.1371/journal.pone.0185378)
Supplement: S2 Text — (DOCX) [file pone.0185378.s007.docx]

**S2 Text. Character list**

| **Nº.** | **CHARACTER/STATE** | **0** | **1** | **2** | **3** | **4** | **REF.** |
| --- | --- | --- | --- | --- | --- | --- | --- |
| **1** | Presence of ossicones | Absent | Present |  |  |  | [2] |
| **2** | Median occipital horns | Absent | Present |  |  |  | [2] |
| **3** | Bifurcated occipital appendage | Absent | Present |  |  |  |  |
| **4** | First ossification of horns | Frontal bone | Dermis/Subsutaneous loose conective tissue |  |  |  | [1] |
| **5** | Mature bony core is | live and coered, live and exposed, dead and exposed | live and covered/live and exposed |  |  |  | [1] |
| **6** | Branching horns | Present | Absent |  |  |  | [1] |
| **7** | Regeneration | Bone tissue and integument | No regeneration |  |  |  | [2] |
| **8** | Fosa nucal | Absent | Present |  |  |  | [3] |
| **9** | Laterally-oriented expansion of the nuchal crest | Absent | Present |  |  |  | [3] |
| **10** | Presence of a deep rectilinear groove between the caudal part of the temporal fossa and the nuchal plane | Absent | Present |  |  |  | [3] |
| **11** | Presence of | 2 ossicones | 4 ossicones |  |  |  |  |
| **12** | Giraffids with only one pair of ossicones section | Elliptical | Circular | Rhomboid |  |  | [2] [4] |
| **13** | Anterior ossciones section | Elliptical | Circular | Expanded-amorphous |  |  |  |
| **14** | Posterior ossciones section | Elliptical | Expanded-amorphous |  |  |  |  |
| **15** | Surface of the ossicone | With ridges | Smoother, without ridges |  |  |  |  |
| **16** | Surface of the ossicone: development of the ridges | Thin and shallow | Strong and deep |  |  |  | [2] |
| **17** | Surface of the ossicone with bumps and expansions | Absent | Present: bumps and deep ridges | Ossicones with large expansions |  |  |  |
| **18** | Ossicone base with large bumps, massive growth at its base like a flange | Absent | Present posteriorly | Present anteriorly |  |  | [2] |
| **19** | Ossicone base with a forward-oriented extension or  ‘wing’ | Absent | Present |  |  |  |  |
| **20** | Ossciones apices | Blunt tip of medium width | Blunt tip wide | Blunt tip very wide, expanded | Pointy tip |  |  |
| **21** | Ossicone polished apices: bare tip and with simple and beveled wear facets | Absent | Present |  |  |  | [2][5] |
| **22** | Presence of bare ossicone apices (porous tip, presence ofan apical constriction) | Absent | Present |  |  |  |  |
| **23** | Ossicone core *Decennatherium*-like | Absent | Present |  |  |  | [2][5] |
| **24** | Giraffids with only one pair of ossicones position regarding the orbit | Above the orbit | Slightly posterior to the orbit, dorsal side of the ossicone begings just above the caudal margin of the orbital rim | Posterior to the orbit |  |  |  |
| **25** | Giraffids with only one pair of ossicones located on the skull roof | Above the orbit, ossicones above orbirtal rims, at the veryedge of the rim | More medially positioned | Even more internially positioned | More medially and posteriorly |  | [5] |
| **26** | Anterior ossicones position | Anterior to the orbit | Above the orbit | Posterior to the orbit |  |  |  |
| **27** | Anterior ossicones located | Medially positioned | Even more internially positioned |  |  |  |  |
| **28** | Posterior ossicones position | Cover the orbital rim, including its most anterior aspect | Behind the posterior margin of the orbit | Posterior to the orbital rim |  |  | [2] |
| **29** | Anterior and posterior ossicones | Separated | Fused at their base, merge at the base |  |  |  |  |
| **30** | Anterior ossicones fused at their base forming a Y | Absent | Present |  |  |  | [2] |
| **31** | Giraffids with only one pair of ossicones oriented | Posterolaterally (like *Bubalus bubalis*) | Posteriorly, angle to the skull base smaller than 90º (like *Hippotragus niger*) | Only slightly posteriorly, almost straight ~90º | Posteromedially |  | [4] |
| **32** | Anterior ossicones oriented | Posterolaterally | Anteriorly | Straight |  |  |  |
| **33** | Posterior ossicones oriented | Laterally | Posterolaterally | Lateroanteriorly |  |  | [2] |
| **34** | Ossicones curvature | Curved | Only slightly curved | Straight |  |  |  |
| **35** | Presence of a median single ossicone | Absent | Present |  |  |  |  |
| **36** | Presence zygomatic ossicones | Absent | Present |  |  |  |  |
| **37** | Anterior ossicone size | Anterior pair smaller | Anterior pair larger |  |  |  |  |
| **38** | Ossicone length within *Samotherium* | Medium | Very long |  |  |  | [2] |
| **39** | Ossicones with internal canals, distal pits: broad boss and constrictions | Absent | Present |  |  |  | [2] |
| **40** | Hornless females/skulls with no ossicones | Absent | Present |  |  |  |  |
| **41** | Anterior Ossicone Index: TD at the Ossicone Base /Length of the Ossicone (Dorsal) (4/1) | 0,5 | < 0,5 | > 0,5 |  |  |  |
| **42** | Posterior Ossicone Index: TD at the Ossicone Base /Length of the Ossicone (Dorsal) (4/1) | 0,15-0,22 | >0,22 |  |  |  |  |
| **43** | Giraffids with only one pair of ossicones Ossicone Base Index: TD at the Ossicone Base/APD at the Ossicone Base (4/3) | < 0,8 | > 0,8 |  |  |  |  |
| **44** | Posterior Ossicone Base Index: TD at the Ossicone Base/APD at the Ossicone Base (4/3) | 0,5-0,7 | > 0,7 |  |  |  |  |
| **45** | Anterior Ossicone Shaft Index: TD at the Middle of the Ossicone/APD at the Middle of the Ossicone (10/11 ) | 1,85< | >1,85 |  |  |  |  |
| **46** | Posterior Ossicone Index/Anterior Ossicone Index*100 | 30-60 | <30 | >60 |  |  |  |
| **47** | Distance between the Ossicones Tips Index: Distance between the tips of the posterior ossicones/Distance between the tips of the anterior ossicones (12post/12ant) | <3 | >3 |  |  |  |  |
| **48** | Superior occipital edge | Extends backwards | Barely extends backwards or does not |  |  |  | [2] |
| **49** | Occipital expansion | Narrow (< 140 mm) | Medium (140-300mm) | Very wide (>300mm) |  |  | [2] [5] |
| **50** | Occipital shape | Narrowest | Very narrow | Typical hour-glass shaped occipitals | Broad occipitals |  | [2] [5] |
| **51** | Frontal sinuns and bosses which hollow the ossicones | Less developed | Developed, expanded above the orbits |  |  |  | [2] |
| **52** | Masseteric morphology angle α | <5 | **05/10/** | >10 |  |  | [2] |
| **53** | Masseteric morphology angle β | <80 | >80 |  |  |  | [2] |
| **54** | Postglenoid/retroarticular process morphology | *Canthumeryx*-like, not expanded | *Okapia*-like, continuous expansion on the articular facet | *Helladotherium*-like, there is only a slight separation of the distal part of the expansion of the articular facet | *Giraffa*-like, there is a groove separating the expansion of the articular facet |  |  |
| **55** | Bullae size | Medium | Large |  |  |  | [2] |
| **56** | Brachycephally | Absent | Present |  |  |  | [6] |
| **57** | Snout downturned | Absent | Present |  |  |  | [2] |
| **58** | Mastoid reduced | Absent | Present |  |  |  | [7] |
| **59** | Skull Width Index: Skull width outside of the Ossicones Basis /Maximum Width of the Cranial Roof | 0,85-1 | >1 | <0,85 |  |  |  |
| **60** | Position of the middle indentation of the hard palate | Very posterior to the M3s | Slightly posterior to the M3s | Between the M3s | Anteriorly positioned betwwen the M2s |  | [2] |
| **61** | Premaxillae elongation | Absent | Present, very elongated |  |  |  |  |
| **62** | Body size increase/gigantism | Absent | Relative increase in size | Notable increase in body size |  |  | [2], [8] |
| **63** | *Palaeotragus* size | Smaller | Larger |  |  |  | [2] |
| **64** | Upper canines | Present sabre-like upper canine in males | Absent |  |  |  | [2] |
| **65** | Bilobed lower canine with enlarged second lobe | Absent | Present |  |  |  |  |
| **66** | Bilobed lower canine morphology | Bigger lobe rounded | Bigger lobe pointed |  |  |  |  |
| **67** | Decidual mandible: d2-d4 length | Very short (˂50 mm) | Short (50-70 mm) | Medium (70-90 mm) | Long (˃90mm) |  |  |
| **68** | Adult mandible: m1-m3 length | Very short (˂40 mm) | Short (40-90 mm) | Medium (90-105 mm) | Medium-Long (105-140 mm ) | Long (˃140mm) |  |
| **69** | Adult mandible: p2-m3 length | Very short (˂100 mm) | Short (110-140 mm) | Medium (140-170 mm) | Medium-Long (170[2]40mm ) | Long (˃240mm) |  |
| **70** | Elongation of the diastema: p2-m3/Distance between p2 and c | 120-140 | >140 | <120 |  |  |  |
| **71** | Distance between p2 and c/Madible Total Length | 20-30 | >30 | <20 |  |  |  |
| **72** | Molarization of p2 and p3 | Absent | Present |  |  |  | [2] |
| **73** | p3 L/W Index | >2 | 1,2[2] | <1,2 |  |  |  |
| **74** | p4 L/W Index | >2 | 1,3[2] | <1,3 |  |  |  |
| **75** | Brachydoncy: m3 hipsodoncy | Less brachydont: 0,6 | More brachydont: <0,6 | Slightly mesodont >0,6 |  |  | [2] [9] |
| **76** | m3 length (mm.) | Low, ˂0,40 | Medium, 0,40-0,55 | High, ˃0,55 |  |  |  |
| **77** | p3 with | Tipical Mesolingual conid present | Absent |  |  |  |  |
| **78** | p3 with isolated conid | Isolated mesolingual conid-like structure | Isolated mesolingual conid wall-like structure |  |  |  |  |
| **79** | p3 with some conid or structure between the anterior and the posterolingual conid | Present | Absent |  |  |  |  |
| **80** | p3 morphology | Mesolingual conid does not project posteriorly | p3 with a bifurcated anterior wing and a strong mesolingual conid, which forming a crest, projects posteriorly until it reaches the lingual end of the entostylid, isolating the entoconid, which is clearly reduced (*Bramatherium*-like) |  |  |  |  |
| **81** | p3 molarized, complete lingual wall | Absent | Intermediate, almost complete | Present |  |  |  |
| **82** | p3 with anterior part bifurcated: anterior styid and conid present | Present | Absent |  |  |  |  |
| **83** | p4 complexity, molarization | Absent | Intermediate | Present, high degree of molarization with the two lobes well separated with a Very deep groove on the labial wall |  |  |  |
| **84** | p4 Second Lobe Index: p4 Posterior Length/ p4 Total Length*100 | <30 | 30-35 | >35 |  |  | [6] |
| **85** | Upper premolars with inward curving styles | Absent | Present |  |  |  | [2] |
| **86** | Upper Premolars round in occlusal view | Absent | Present |  |  |  | [2] |
| **87** | Neck elongation | Absent, short cervicals | Intermediate | Slight elongation | Elongation, highly elongated cervicals |  | [2] [10] |
| **88** | Atlas Index: Maximum Width/ Total Length | >90 | <90 |  |  |  |  |
| **89** | Axis Index: Maximum Width/ Total Length | 40-70 | 70-100 | >100 | <40 |  |  |
| **90** | Acromion in the scapula | Present | Absent |  |  |  | [11][12][13] |
| **91** | Development of the humerus major lateral tuberosity | Intermediate development | Highly developed | Absent |  |  |  |
| **92** | Radius Index: Diaphysis TD/Total Length*100 | Medium, 11-15 | Robust, >15 | Slender, <11 |  |  |  |
| **93** | Tibia Index: Diaphysis TD/Total Length*100 | Slender, <9 | Medium, 9-12 | Robust, >12 |  |  |  |
| **94** | Metacarpal III-IV Robustness Index: Diaphysis TD/Total Length*100 | Slender, <9 | Medium, 9-10,5 | Robust, 10,5-16 | Very robust, >16 |  |  |
| **95** | Metacarpal III-IV Proximal Index: Proximal TD/Total Length*100, indicates metacarpal proximal epiphysis widening | Very slender, ˂15 | Slender, 15-19 | Medium, 19[2]5 | Robust, 25-31 | Very robust, ˃31 |  |
| **96** | Metacarpal III-IV longation | Very Short ( ˂290 mm) | Short (290-370 mm) | Medium (370-470 mm) | Long (˃470) |  |  |
| **97** | Metacarpal III-IV palmar trough depth | Medium | Very deep | Very shallow |  |  |  |
| **98** | Metacarpal III-IV palmar trough curvature | Medium | Very high | Very low |  |  |  |
| **99** | Metatarsal III-IV Robustnesss Index: Diaphysis TD/Total Length*100 | Very slender (˂,8) | Slender (8-11) | Robust (˃11) |  |  |  |
| **100** | Metatarsal III-IV Proximal Index: Proximal TD/Total Length*100 | Slender (9-13) | Medium robustness (13-16) | Robust (16[2]2) | Very robust (˃22) |  |  |
| **101** | Metatarsal III-IV elongation | Very Short ( ˂250 mm) | Short (250-370 mm) | Medium (370-450 mm) | Long (450-600 mm) | Very Long (˃600) |  |
| **102** | Metatarsal III-IV palmar trough depth | Medium | Very deep | Very shallow |  |  |  |
| **103** | Metatarsal III-IV Palmar trough curvature | Medium | Very high | Very low |  |  |  |
| **104** | Metacarpal vs Metatarsal size | Same size | Metatarsal longer than the metacarpal |  |  |  | [2] |
| **105** | Astragalus morphology | Plesiomorphic | Apomorphic, wide |  |  |  | [2] |
| **106** | Cubonavicular distoplantar tuberosity and an insertion groove for the peroneus longus muscle | Barely developed | Very developed and straighter | Very developed and triangular | Very developed and curved |  |  |
| **107** | Fusion of cuneiform bones with cubonavicular | Entocuneiform separated from ectomesocuneiform (ectomesocuneiform + entocuneiform + cubonavicular) | Presence of entomesoectocuneiform (ectomesocuneiform-entocuneiform + cubonavicular) | Fusion of cuneiform bones with navicular-cuboid (ectomesocuneiform-entocuneiform-cubonavicular) |  |  |  |
| **108** | First phalanx Index: Diaphysis TD/Total Length*100 | Slender, <25 | Medium, 25-30 | Robust, >30 |  |  |  |
| **109** | First phalanx palmar/plantar proximal rugose areas for ligament insertion, rugose surfaces proximoplantarly/bulgy proximal sides | Absent | Present, onlyt slightly developed | Present, more developed, more rugose |  |  |  |
| **110** | Second phalanx Index: Diaphysis TD/Total Length*100 | Slender, <35 | Medium, 35-45 | Robust, 45-80 | Very Robust, ˃80 |  |  |
| **111** | Third phalanx dorsal process for the insertion of the extensor digitorum | Absent | Well-developed | Barely present |  |  |  |

**Character list: references**

1. Davis EB, Brakora KA, Lee AH. Evolution of ruminant headgear: a review. Proc R Soc B. 2011; 278-288.

2. Solounias N. Family Giraffidae. . In: In Prothero DR, Foss SE, editors. The Evolution of Artiodactyls. Baltimore: The Johns Hopkins University Press; 2007. pp. 257-277.

3. Sánchez I, Cantalapiedra J, Ríos M, Quiralte V, Morales J. Systematics and Evolution of the Miocene Three-Horned Palaeomerycid Ruminants (Mammalia, Cetartiodactyla). PLOS ONE. 2015;10(12):e0143034.

4. Kostopoulos DS, Koliadimou KK, Koufos GD. The giraffids (Mammalia, Artiodactyla) from the late Miocene mammalian localities of Nikiti (Macedonia, Greece). Palaeontogr Abt A. 1996; 239(1-3):61-88.

5. Hou S, Danowitz M, Sammis J, Solounias N. Dead ossicones, and other characters describing Palaeotraginae (Giraffidae; Mammalia) based on new material from Gansu, Central China. Zitteliana. 2014; B 32:1-8.

6. Hamilton WR. Fossil Giraffes from the Miocene of Africa and a Revision of the Phylogeny of the Giraffoidea.

. Philos Trans R Soc Lond B Biol Sci. 1978; 283(996):165-229.

7. Geraads D. Remarques sur la systématique et la phylogénie des Giraffidae (Artiodactyla, Mammalia). Geobios. 1986; 19(4):465-477.

8. Basu C, Falkingham PL, Hutchinson JR. The extinct, giant giraffid *Sivatherium giganteum*: skeletal reconstruction and body mass estimation. Biol Lett. 2016; 12(1). Available from: [10.1098/rsbl.2015.0940](https://dx.doi.org/10.1098%2Frsbl.2015.0940).

9. Samiullah K, Akhtar M, Ghaffar A, Khan MA. *Giraffokeryx punjabiensis* (Artiodactyla, Ruminantia, Giraffidae) from Lower Siwaliks (Chinji Formation) of Dhok Bun Ameer Khatoon, Pakistan. J Sci Technol MSU. 2012; 1(30): 1-24.

10. Badlangana NL, Adams JW, Manger PR. The giraffe (*Giraffa camelopardalis*) cervical vertebral column: a heuristic example in understanding evolutionary processes? Zool J Linn Soc. 2009; 155: 736-757.

11. Hamilton WR. The lower miocene ruminants of Gebel Zelten, Libya.. Bull Br Mus (Nat Hist) Geol. 1973; 21(3):73-150.

12. Bohlin B. Die Familie Giraffidae. . Palaeont Sin. 1926; C 4 (I):1-170.

13. Churcher C. Two new upper Miocene giraffids from Fort Ternan, Kenya, East Africa: *Palaeotragus primaevus* n. sp. and *Samotherium africanum* n. sp. Fossil vertebrates of Africa. 1970; 2:1-106.
